# Continuous electroproduction of formate via CO_2_ reduction on local symmetry-broken single-atom catalysts

**DOI:** 10.1038/s41467-023-42539-1

**Published:** 2023-10-27

**Authors:** Juncai Dong, Yangyang Liu, Jiajing Pei, Haijing Li, Shufang Ji, Lei Shi, Yaning Zhang, Can Li, Cheng Tang, Jiangwen Liao, Shiqing Xu, Huabin Zhang, Qi Li, Shenlong Zhao

**Affiliations:** 1 Beijing Synchrotron Radiation Facility, Institute of High Energy Physics, Chinese Academy of Sciences, Beijing, 100049 China; 2 CAS Key Laboratory of Nanosystem and Hierarchical Fabrication, CAS Center for Excellence in Nanoscience, National Center for Nanoscience and Technology, Beijing, 100190 China; 3 School of Chemical and Biomolecular Engineering, The University of Sydney, Sydney, NSW 2006 Australia; 4 Department of Chemistry, University of Toronto, Ontario, M5S3H6 Canada; 5 Key Laboratory of Rare Earth Optoelectronic Materials and Devices of Zhejiang Province, College of Optical and Electronic Technology, China Jiliang University, Hangzhou, 310018 China; 6 Beijing Key Laboratory of Green Chemical Reaction Engineering and Technology, Department of Chemical Engineering, Tsinghua University, Beijing, 100084 China; 7 KAUST Catalysis Center, King Abdullah University of Science and Technology, Thuwal, 23955-6900 Saudi Arabia

**Keywords:** Electrocatalysis, Electrocatalysis

## Abstract

Atomic-level coordination engineering is an efficient strategy for tuning the catalytic performance of single-atom catalysts (SACs). However, their rational design has so far been plagued by the lack of a universal correlation between the coordination symmetry and catalytic properties. Herein, we synthesised planar-symmetry-broken CuN_3_ (PSB-CuN_3_) SACs through microwave heating for electrocatalytic CO_2_ reduction. Remarkably, the as-prepared catalysts exhibited a selectivity of 94.3% towards formate at −0.73 V vs. RHE, surpassing the symmetrical CuN_4_ catalyst (72.4% at −0.93 V vs. RHE). In a flow cell equipped with a PSB-CuN_3_ electrode, over 90% formate selectivity was maintained at an average current density of 94.4 mA cm^−2^ during 100 h operation. By combining definitive structural identification with operando X-ray spectroscopy and theoretical calculations, we revealed that the intrinsic local symmetry breaking from planar *D*_4*h*_ configuration induces an unconventional *dsp* hybridisation, and thus a strong correlation between the catalytic activity and microenvironment of metal centre (i.e., coordination number and distortion), with high preference for formate production in CuN_3_ moiety. The finding opens an avenue for designing efficient SACs with specific local symmetries for selective electrocatalysis.

## Abstract (web summary)

Atomic-level coordination influences the properties of single-atom-catalysts but is difficult to precisely engineer. Here, authors study the role of local symmetry manipulation, finding planar-symmetry-broken CuN_3_ catalysts outperform highly symmetrical CuN_4_ for CO_2_ electroreduction to formic acid.

## Introduction

Formic acid has long been regarded as an important liquid feedstock for valuable chemicals synthesis and ideal hydrogen carrier material owing to its high volumetric (53.4 g L^−1^) and moderate gravimetric (4.4 wt%) hydrogen storage capacity in ambient conditions^1,2^. Producing formic acid via the electrochemical reduction of carbon dioxide (CO_2_) is therefore attracting widespread attention^3–5^. Considering both the thermodynamical and kinetic energy barriers, the energy consumption for electroreduction of CO_2_ to formic acid (${{{{{\rm{C}}}}}}{{{{{{\rm{O}}}}}}}_{2}+2{{{{{{\rm{H}}}}}}}^{+}+2{{{{{{\rm{e}}}}}}}^{-}\to {{{{{\rm{HCOOH}}}}}}$, −0.19 V vs. RHE) is relatively less compared with other products like CH_3_OH and CH_4_. In the past years, various metals (e.g., Cu, Sn, Bi) and their alloys have been prepared as electrocatalysts for electrocatalytic CO_2_ to formic acid^6–10^. Although significant progress has been made in reducing the overpotential for CO_2_ electroreduction to formic acid^11–14^_,_ achieving the necessary faradaic efficiency (FE) and current density for practical applications remains a challenge^15–17^. Hence, there is a pressing demand to develop highly selective and energy-efficient catalysts for the electroreduction of CO_2_ to formic acid.

Carbon-supported single-atom catalysts (SACs), most of which usually adopt a prototypical MN_4_ moiety with local planar-like *D*_4*h*_ symmetry, show great promise for accelerating the kinetics of heterogeneous catalysis owing to their unique physicochemical properties^18–20^. Though substantial progress has been made in activity improvement, recent studies reveal that the highly symmetrical MN_4_ architecture actually imposes serious limitations on the electronic configuration regulation of the active metal site^21–23^. To lift the confinement and better tune the electronic structures of the active sites, various coordination engineering strategies such as tuning the coordination species and the coordination number of the active centres, heteroatom substitution, neighbouring metal interactions are developed^24–27^. For example, the coordination-engineered M-N_*x*_/N_*x*_L_*y*_ (L=C, O, P, S) exhibits excellent performance in oxygen-involved electrocatalysis with a medium bond strength (494 kJ mol^−1^ for O=O bond)^23,28–33^, and great potentials for activating the molecules with high bond energy such as C=O in CO_2_ (750 kJ mol^−1^)^34–39^. Essentially, local symmetry breaking from the planar-like *D*_4*h*_ via coordination engineering can redistribute the electronic (anti-)bonding states on specific *d*/*s*/*p* orbitals of the metal sites along with the energy scale and thereby maximumly regulate their hybridisation interaction in a rationally pre-selected local symmetry. This local symmetry manipulation could thus facilitate the adsorption and activation of CO_2_ molecular as well as reactive intermediates and optimise the reaction pathways for CO_2_ reduction reaction (CO_2_RR) with high activity and selectivity^40^. Unfortunately, except for the extrinsic addition of various axial ligands, there is no report about the intrinsic local symmetry breaking in M-N_*x*_ SACs for electrocatalytic CO_2_ reduction to date. Moreover, despite the extensive endeavours, the underlying mechanism that governs the structure−functionality relationship and the universal design principle to manipulate the local coordination symmetry remains unclear.

Herein, the local planar-symmetry-broken CuN_3_ (PSB-CuN_3_) SACs were synthesised by a facile and simple microwave process for electroreduction of CO_2_. The as-prepared PSB-CuN_3_ delivers a selectivity of 94.3% for electroreduction of CO_2_ into formate at a potential of −0.73 V vs. RHE, which outperforms the CuN_4_ SACs with local planar *D*_4*h*_ symmetry (PS-CuN_4_) with a maximum selectivity of 72.4% at a given potential of −0.93 V vs. RHE. Impressively, the flow cell equipped with the PSB-CuN_3_ electrode exhibits over 90% formate selectivity at an average reduction current density of 94.4 mA cm^−2^ during 100 h continuous operation at an applied potential of −0.95 V vs. RHE. By combining definitive structural identification with ex-situ/operando X-ray spectroscopy characterisations and systematic theoretical calculations, we reveal that the intrinsic local symmetry breaking from the planar-like *D*_4*h*_ to *C*_2*v*_ configuration induces an unconventional *dsp* hybridisation. It causes the catalytic activity unconformable to the widely used *d*-band centre theory, but strongly correlated with the local environment of the metal centre (i.e., coordination number and geometric distortion), which can serve as a universal descriptor to predict the activities of H_2_/CO/HCOOH productions for graphene-based SACs, with a high preference for formate production in CuN_3_ moiety.

## Results and discussion

### Synthesis and structural characterisation of PSB-CuN_3_ catalyst

**Fig. 1:**
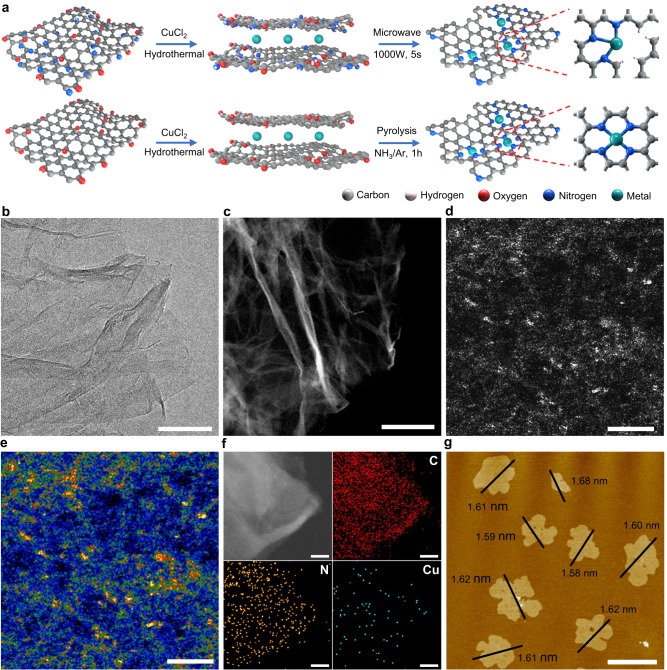
Synthesis and structural characterisations of PSB-CuN_3_ catalyst. **a** Schematic illustration of the preparation strategy for PSB-CuN_3_ and PS-CuN_4_. **b**–**e** TEM image (**b**; scale bar, 200 nm), HAADF-STEM image (**c** scale bar, 200 nm), AC-HAADF-STEM image (**d** scale bar, 1 nm) and corresponding intensity maps (**e**) of PSB-CuN_3_. **f** EDS mapping images of PSB-CuN_3_ (C, red; N, orange; Cu, blue; scale bar, 10 nm). Note that the molybdenum grid was used for all STEM measurements. **g** AFM image of as-prepared PSB-CuN_3_ (scale bar, 2 μm), showing measured dimensions of individual nanosheets.

PSB-CuN_3_ is synthesised via a microwave heating strategy, as schematically illustrated in Fig. 1a. Amine-functionalised graphene nanosheets (AGNs) are used to immobilise the Cu^2+^ ions through an electrostatic interaction. Then, PSB**-**CuN_3_ is obtained through a rapid microwave heating process within five seconds. In comparison to conventional thermal annealing, microwave-induced instant heating leads the local environment in the material to change dramatically, facilitating the formation of a local symmetry-breaking architecture. The ultrathin morphology of as-synthesised PSB-CuN_3_ can be discerned well by transmission electron microscopy (TEM) and high-angle annular dark-field scanning TEM (HAADF-STEM) images, in which the edges of the two dimentional (2D) nanosheets spontaneously curl upon microwave heating (Fig. 1b, c, Supplementary Fig. 1a). The aberration-corrected HAADF-STEM (AC-HAADF-STEM) image (Supplementary Fig. 1b) shows that no Cu-derived nanoparticles can be observed, which agrees with the X-ray diffraction (XRD) result (Supplementary Fig. 2). Meanwhile, the atomic dispersion of single Cu atoms can be directly identified as the obvious individual bright dots in the high-magnification AC-HAADF-STEM images (Fig. 1d, e). Energy-dispersive X-ray spectroscopy (EDS) mappings analysis shows C, N and Cu elements are homogeneously dispersed on the entire architecture of PSB-CuN_3_ (Fig. 1f) with a content of 96.7 at %, 2.82 at % and 0.48 at %, respectively (Supplementary Fig. 3). And, the atomic force microscopy (AFM) measurement (Fig. 1g) is performed to present the average thickness of the PSB-CuN_3_ (~1.61 nm). The N_2_ adsorption-desorption isotherms shows a high specific surface area of ~510 m^2^ g^−1^ with dominant pore sizes of ~4 nm (Supplementary Fig. 4). The PS-CuN_4_ catalyst prepared by conventional thermal annealing shows similar physiochemical information (Supplementary Figs. 2–6), demonstrating the high controllability for materials synthesis. Subsequently, chemical states of the elements in PSB-CuN_3_ and PS-CuN_4_ are investigated by X-ray photoelectron spectroscopy (XPS) analysis (Supplementary Figs. 7–9). While three types of graphitic (401.2 eV), pyrrolic (399.4 eV) and pyridinic (398.6 eV) N species for stabilising the Cu atoms exhibit no obvious difference, the Cu 2*p* spectrum of PSB**-**CuN_3_ possesses two dominant peaks at binding energies of 932.0 eV (2*p*_3/2_) and 951.8 eV (2*p*_1/2_) that are obviously lower than the characteristic binding energies of 934.3 and 954.1 eV for the Cu^2+^ in PS-CuN_4_, implying a lower valence state of Cu^1+^ in PSB**-**CuN_3_. Cu LMM spectra are obtained to further demonstrate the chemical valence of the two samples (Supplementary Fig. 10). While PSB-CuN_3_ is dominated by Cu^1+^ species located at 571.1 eV, PS-CuN_4_ is dominated by Cu^2+^ species located at 572.0 eV^41^. In addition to the chemical states, Raman spectra show there are a larger number of structural defects in PSB-CuN_3_ compared with PS-CuN_4_ (Supplementary Fig. 11). Thus, the above results suggest that the Cu species in PSB**-**CuN_3_ catalyst are likely to be dispersed as unusual low-valent mononuclear centres on largely defective graphene nanosheets.

### Atomic and electronic structure analysis of PSB-CuN_3_ catalyst by X-ray absorption fine structure spectroscopy

**Fig. 2:**
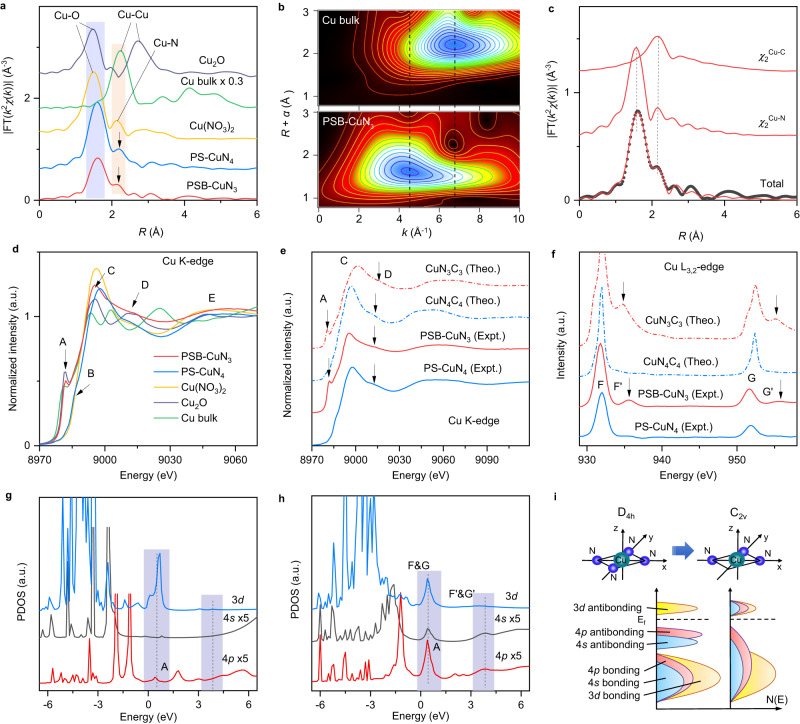
Atomic and electronic structure characterisation by XAFS spectroscopy. **a** Fourier-transformed magnitudes of the experimental Cu *K*-edge EXAFS signals of PSB-CuN_3_ and PS-CuN_4_ along with reference samples. The Fourier transforms are not corrected for phase shifts. **b** EXAFS wavelet transforms for the PSB-CuN_3_ and reference samples. The contour maxima at ~4.2 and 7.0 Å^−1^ (dashed lines) are associated with the Cu–N/C and Cu–Cu contributions, respectively. **c** Cu *K*-edge EXAFS analysis of PSB-CuN_3_ with the Cu–N and Cu–C two-body backscattering signals *χ*_2_ included in the fit. The calculated (red line) and measured (black dots) spectra show excellent agreement (Supplementary Table 1). **d** Experimental Cu *K*-edge XANES spectra of PSB-CuN_3_, PS-CuN_4_ and reference samples. **e**, **f** Comparison between the experimental Cu *K*-edge (**e**) and *L*_3,2_-edge (**f**) XANES spectra of PS-CuN_4_ and PSB-CuN_3_ and the theoretical spectra calculated for the depicted CuN_4_C_4_ and CuN_3_C_3_ structures (Supplementary Fig. 21). **g**, **h** The calculated 3*d*, 4 *s* and 4*p* projected densities of states on the Cu sites in CuN_4_C_4_ (**g**) and CuN_3_C_3_ (**h**) structures. **i** Schematic diagrams of band shifts and hybridisation for the planar CuN_4_ moiety with local *D*_4*h*_ symmetry and the defective CuN_3_ moiety with lower local C_2*v*_ symmetry. a.u. arbitrary units.

The coordination environment and chemical state of PSB-CuN_3_ are further explored by element-selective X-ray absorption fine structure (XAFS) spectroscopy analysis including extended-XAFS (EXAFS) and X-ray absorption near-edge structure (XANES). Figure 2a shows the Cu *K*-edge EXAFS Fourier-transformed (FT) magnitudes. Both PSB-CuN_3_ and PS-CuN_4_ exhibit a major peak at ~1.60 Å and a minor satellite peak at ~2.20 Å, which is similar to the archetypical profile of pyridinic-N-based MN_4_C_4_ motifs^21^. Concurrently, the EXAFS wavelet-transform analysis is performed to more clearly discriminate the coordination atoms^42^. Only one intensity maximum at ~4.2 Å^–1^ is detected in the two catalysts (Fig. 2b), which confirms the assignment of the major and minor peaks to the Cu–N/C bonding (Supplementary Fig. 12a). However, the two peaks intensities in PSB-CuN_3_ are obviously weaker than that in PS-CuN_4_ (Supplementary Fig. 12b), which implies a lower coordination configuration for Cu atoms in PSB-CuN_3_. Subsequently, quantitative EXAFS curve-fitting analysis is carried out to investigate the coordination configuration (Fig. 2c, Supplementary Figs. 13–17; Supplementary Table 1). Coordination numbers of the first N and second C coordination spheres in PS-CuN_4_ are estimated to be 4.0 and 4.1 at distances of 2.00 and 2.67 Å, respectively, supporting the adoption of a pyridinic-N-based CuN_4_C_4_ configuration (Supplementary Fig. 17c). By contrast, the best-fit for PSB-CuN_3_ demonstrates reduced coordination numbers (2.8 for Cu–N and 2.9 for Cu–C) with contracted interatomic distances (1.98 Å for the first Cu–N and 2.65 Å for Cu–C) for the first and second coordination spheres, suggesting a defective pyridinic-N-based CuN_3_C_3_ configuration (Fig. 2c).

Due to the higher sensitivity to the 3D arrangement of atoms around the photo-absorber, XANES spectroscopy is applied to better identify the atomic-site structures^43,44^. Figure 2d shows the hard Cu *K*-edge XANES spectra for the samples along with references. The PS-CuN_4_ exhibits a shoulder feature B at ~8987 eV, which is close to that of Cu(NO_3_)_2_. It arises from the 1*s*→4*p*_*z*_ transition of a planar-like Cu^2+^ configuration^45,46^. However, in the spectrum of PSB-CuN_3_, a pronounced peak A is observed at a much lower energy of ~8982 eV as in Cu_2_O, signifying an unusual Cu^1+^ configuration. Moreover, PSB-CuN_3_ shows a more flattened profile for the post-edge features C, D and E compared with PS-CuN_4_. This tendency is also corroborated by the soft XANES results (Supplementary Figs. 18–20). And, the Cu *L*_3,2_-edge spectra reveal an emergence of two extra post-edge resonant peaks from PS-CuN_4_ to PSB-CuN_3_ that coincides with the positions of Cu_2_O (Supplementary Fig. 20). Those hard and soft XANES differences indicate a peculiar Cu^1+^ species in PSB-CuN_3_ and a planar-like Cu^2+^ species in PS-CuN_4_, consistent with the XPS results (Supplementary Figs. 9 and 10). Then, as guided by the EXAFS results, we constructed various CuN_*x*_C_*y*_ structural models with different local symmetry (Supplementary Figs. 21 and 22) and resorted to XANES simulation to identify the specific configuration. Figure 2e, f shows that the CuN_4_C_4_ moiety with local *D*_4*h*_ symmetry (Note: The C_4_ symmetry axis is perpendicular to the plane) reproduces properly the experimental *K*- and *L*_3,2_-edge XANES profiles of PS-CuN_4_. In addition, when a N defect is introduced, the obtained CuN_3_C_3_ moiety shows a local symmetry breaking from *D*_4*h*_ to *C*_2*v*_ (Note: The remaining C_2_ symmetry axis is in the plane) and it produces an excellent agreement to the experimental profiles of PSB-CuN_3_, particularly for the characteristic peaks A and D at *K*-edge (Fig. 2e) and for the peaks F’ and G’ at *L*_3,2_-edge (Fig. 2f). By contrast, more N defects in CuN_2_C_2_ moiety lead to a dramatic discrepancy in the profiles (see Supplementary Fig. 23). The probability for the substitution of the N atoms in the first coordination sphere by C atoms can also be excluded (Supplementary Figs. 24 and 25, Supplementary Table 2 and Supplementary Note 1). Therefore, the combination of EXAFS and XANES analyses has unambiguously revealed a dominance of defective CuN_3_C_3_ moiety with lower local *C*_2*v*_ symmetry and mono-valence implanted in a graphene sheet for PSB-CuN_3_ catalyst.

Because the *K*-edge (1s→4*p* transition) and *L*_3,2_-edge (2*p*→3*d* transition) XANES can probe the unoccupied density of states on the 4*p* and 3*d* orbitals of the Cu sites, the emergent resonant features (A, F’ and G’) in PSB-CuN_3_ catalyst indicate a unique *p* and *d* electronic distribution that can be regulated by the local coordination symmetry. Detailed analysis on the density of states (Fig. 2g–i, Supplementary Figs. 26 and 27 and Supplementary Note 2) unveils that the local symmetry lowering from *D*_4*h*_ in CuN_4_C_4_ to *C*_2*v*_ in CuN_3_C_3_ can drive an obvious upshift of the anti-bonding 4 *s* and 4*p* state to above the Fermi energy and finally trigger a dramatic *dsp* hybridisation at the energy positions of peaks A/F/G and F’/G’, where the *p* orbital is dominated by the in-plane *p*_*x*_/*p*_*y*_ orbital. The local symmetry lowering to *C*_2*v*_ in CuN_2_C_2_ promotes a *dsp*^2^ hybridisation with the *p* orbital arising mainly from the in-plane *p*_*x*_ and *p*_*y*_ orbitals (Supplementary Fig. 28 and Supplementary Note 2). Accordingly, those anti-bonding state upshifts induced by local symmetry breaking in defective CuN_*x*_C_*y*_ moieties can be pivotal to modifying the adsorption strength of the reactive intermediates and thus optimise the reaction pathways for CO_2_RR with high activity and selectivity.

### Evaluation of CO_2_RR performance

**Fig. 3:**
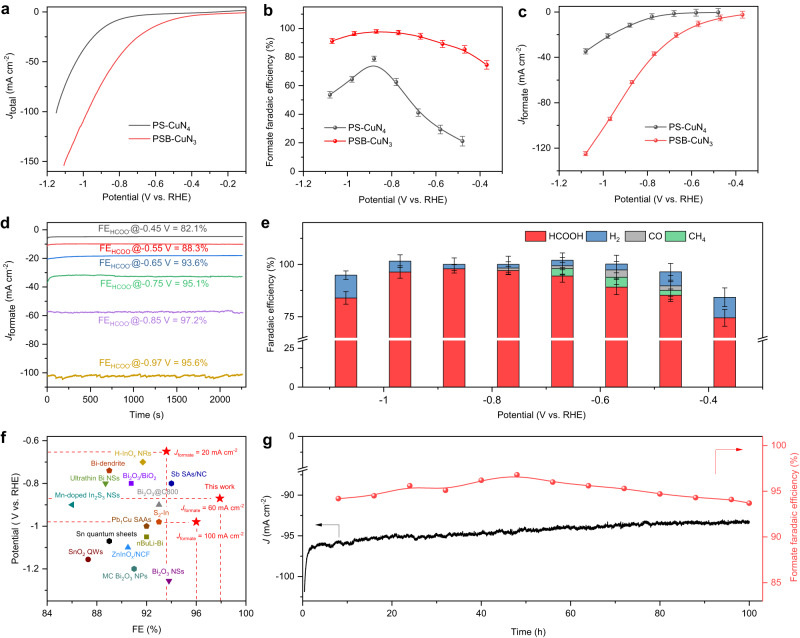
Evaluation of CO_2_RR catalytic activity by electrochemical measurements. **a** Linear sweep voltammetry curves of PSB-CuN_3_ and PS-CuN_4_ in a flow-cell setup using 0.5 M KHCO_3_ electrolyte at 25 °C. **b** The corresponding formate FEs under different potentials. The maximal formate FE of PSB-CuN_3_ is 97.9% at −0.87 V vs. RHE. **c** Comparison of partial current density for CO_2_-to-formate conversion between PSB-CuN_3_ and PS-CuN_4_. The maximal formate partial current on PSB-CuN_3_ reached 125 mA cm^−2^ at −1.1 V vs. RHE, which represents 3.6 folds higher than that of PS-CuN_4_. **d** Chronoamperometric responses of PSB-CuN_3_ in CO_2_-saturated electrolyte at different potentials. The potential was corrected with 100% *iR* compensation. **e** The corresponding FEs of different products under different potentials on PSB-CuN_3_. Note that the total FEs are not complete 100% due to systems errors and double layer current of catalysts. The presented values represent the mean, while the error bars indicate the standard deviation, based on three independent measurements. **f** Comparison of formate FEs and overpotentials for PSB-CuN_3_ and various catalysts reported at similar conditions (See the details in Supplementary Table 5). **g** Long-term stability test of PSB-CuN_3_ at a potential of −0.95 V vs. RHE.

The intrinsic CO_2_RR activity of the as-prepared samples was performed in a gas-tight H-type cell by coating a thin electrocatalyst layer on the carbon fibre paper with a catalyst loading of 0.5 mg cm^−2^. The electrolytes pH (pH = 7.3 for CO_2_ saturated 0.5 M KHCO_3_) and the potential of the reference electrode were checked before the test to ensure the reliability of the as-obtained electrochemical results (Supplementary Figs. 29 and 30). Linear sweep voltammetry (LSV) is employed to obtain polarisation curves of PSB-CuN_3_, PS-CuN_4_ and AGNs electrodes in the Ar and CO_2_ saturated 0.5 M KHCO_3_ aqueous solution. As shown in Supplementary Fig. 31, the reduction current increases greatly when the electrolyte solution is saturated with CO_2_. Supplementary Fig. 32 shows a sharp increased cathode current response started at an onset potential of −0.41 V vs. RHE observed from the PSB-CuN_3_ electrode. By contrast, PS-CuN_4_ exhibits an onset potential (−0.72 V vs. RHE) under identical conditions. Further, chronoamperometric measurements were carried out at different potentials to quantitively identify product distributions using online gas chromatography and nuclear magnetic resonance (NMR) spectroscopy (Supplementary Figs. 33 and 34). As shown in Supplementary Fig. 35 and Supplementary Table 3, formate is the only liquid product from the CO_2_RR, with a certain amount of gaseous products including methane, carbon monoxide and hydrogen. No other C_1_ and C_2_ liquids (i.e., methanol, ethanol, n-propanol and acetone) were detected (Supplementary Fig. 34). Impressive FEs (HCOO^−^) (>90%) were obtained with the PSB-CuN_3_ throughout the potential range (−0.75 to −1.0 V vs. RHE). On the contrary, the maximal FEs of formate for PS-CuN_4_ and AGNs electrodes are 72.4% and 9.5% under identical testing conditions. This suggests the PSB-CuN_3_ moiety should be responsible for the high selectivity for formate. Besides, chronoamperometric measurement was carried out to investigate the stability of the as-prepared PSB-CuN_3_. As shown in Supplementary Fig. 36, the FE of the PSB-CuN_3_ electrode is ~92% after 16 h electrocatalysis compared with PS-CuN_4_ (~27%). To investigate CO_2_ reduction performance under industrial level current of PSB-CuN_3_, a flow cell equipped with gas diffusion electrodes (GDEs) was fabricated (Supplementary Fig. 37). Figure 3a shows that the GDE with PSB-CuN_3_ could be operated at a current density of 150 mA cm^−2^, with modest overpotentials in 0.5 M KHCO_3_ solution, which is much higher than the PS-CuN_4_ under the same applied potential. The optimal FEs of PSB-CuN_3_ electrode is 97.9%, which is higher than the PS-CuN_4_ (78.8%) (Fig. 3b and Supplementary Table 4). With this FE, a CO_2_RR to formate partial current density of ~62 mA cm^−2^ was achieved (Fig. 3c). Moreover, the FE of PSB-CuN_3_ for HCOO^−^ can be maintained over 90% through a wide applied potential range from −0.65 to −0.97 V vs. RHE (Fig. 3d, e), exhibiting excellent selectivity for CO_2_RR-to-formate. It should be stressed that both the selectivity and activity of the PSB-CuN_3_ are comparable to the previously best-reported CO_2_RR-to-HCOO^−^ catalysts (Fig. 3f and Supplementary Table 5). Turnover frequency (TOF), as a key parameter for CO_2_RR efficiency assessment, is then calculated based on the standard equation. As shown in Supplementary Fig. 38, the maximum TOF value of PSB-CuN_3_ is 30825.3 h^−1^ at a given potential of −1.1 V vs. RHE, considerably higher than PS-CuN_4_ (8558.8 h^−1^) and is comparable to the recently best-reported CO_2_RR-to-HCOO^−^ catalysts (Supplementary Table 5). To show the scalability of the as-fabricated flow cell with PSB-CuN_3_ electrode, the long-term durability was tested under the large current density. As shown in Fig. 3g, the PSB-CuN_3_ electrode maintains an average current density of 94.4 mA cm^−2^ with over 90% FE_formate_ during 100 h testing. By contrast, the PS-CuN_4_ electrode shows 58.3% of the initial reduction current of 25 mA cm^−2^ with an average FE of 68% (Supplementary Fig. 39). Further structural stability characterisation by AC-HAADF-STEM and XAFS suggest that the Cu atom and its coordinating matrix in PSB-CuN_3_ are robust enough to withstand the CO_2_RR operation condition (see below detailed discussion). Thus, the PSB-CuN_3_ electrode could be readily incorporated into practical CO_2_RR electrolysers because of its high activity, selectivity and durability at large current densities.

### Mechanism of CO_2_ activation and reduction by first-principles calculation and operando spectroscopies

To understand the origin of high efficiency toward formate generation on PSB-CuN_3_ catalyst, first-principles calculations were carried out for the electroreduction process of CO_2_ on various graphene-based CuN_*x*_C_*y*_ structures. We considered the reaction pathways for CO, formate, methanol and methane productions as well as the competing hydrogen evolution reaction (HER). For the formation of intermediate *OCHO/*COOH, two mechanisms were considered, i.e., a reaction with *H via a CO_2_ insertion reaction into the metal–hydrogen bond and a direct protonation with H^+^ from solution^47,48^. The optimised structures of the intermediates and the Gibbs free-energy diagrams at zero electrode potential show remarkable response to the coordination environment (Supplementary Figs. 40–47, Supplementary Tables 6 and 7). On the CuN_4_C_4_ and CuN_3_C_3_ structures, the different adsorbed states of *COO^−^ and *OCO^−^ for CO_2_ via the first electron addition are suggested to be the potential limiting steps for CO and formate productions, respectively, and the *OCO^−^ formation is more energetically favoured than *COO^−^. By contrast, on the CuN_2_C_2_ structure, the potential limiting steps shift to the desorption of CO and formate. The pathways initiated by *H adsorption do not show any preference than the pathways initiated by *COO^−^/*OCO^−^ adsorption. It is well documented that the reduction of CO_2_ to CH_4_ and CH_3_OH shares similar pathways until the formation of *CO intermediate, and the potential limiting steps are shown to be ${}^{*}+{{{{{\rm{C}}}}}}{{{{{{\rm{O}}}}}}}_{2}\to {}^{*}{{{{{\rm{C}}}}}}{{{{{\rm{O}}}}}}{{{{{{\rm{O}}}}}}}^{-}$ for CuN_4_C_4_, ${}^{*}{{{{{\rm{CO}}}}}}\to {}^{*}{{{{{\rm{CHO}}}}}}$ for CuN_3_C_3_ and CuN_2_C_2_ (Supplementary Fig. 47). The magnitudes of the corresponding theoretical limiting free-energy differences (*ΔG*_L_) are summarised in Supplementary Fig. 47. While the three distinctly coordinated catalysts exhibit a flipped volcano relationship for CO and formate activities, the predicted HER and CH_4_/CH_3_OH activities are simultaneously enhanced by lowering the local symmetry via introducing N vacancy. In comparison to the highly symmetrical CuN_4_C_4_, it leads to a much lower *ΔG*_L_ (0.23 eV) for the formate pathway than the CO (0.68 eV), CH_4_/CH_3_OH (0.78 eV) and HER (1.01 eV) pathways in the local symmetry-broken CuN_3_C_3_. But, this tendency is reversed by more N vacancies in CuN_2_C_2_ which exhibits a preference to the HER pathway. The high selectivity for CO_2_ conversion to formate in CuN_3_C_3_ can be also appreciated from the large difference between the *ΔG*_L_ for CO_2_ reduction and HER. The probability for C_2_ products generation was also considered by examining the first C–C coupling of two *CO species that is crucial for the formation of C_2_ products (Supplementary Fig. 47). This step is found to have a large *ΔG*_L_ (1.79 eV on CuN_4_C_4_ and 1.67 eV on CuN_3_C_3_), indicating a formidable C–C coupling of *CO species for the generation of C_2_ products. Thus, the HCOOH production is thermodynamically preferred than the generation of other C_1_ and C_2_ products and H_2_ on the CuN_3_C_3_ structure at zero electrode potential.

**Fig. 4:**
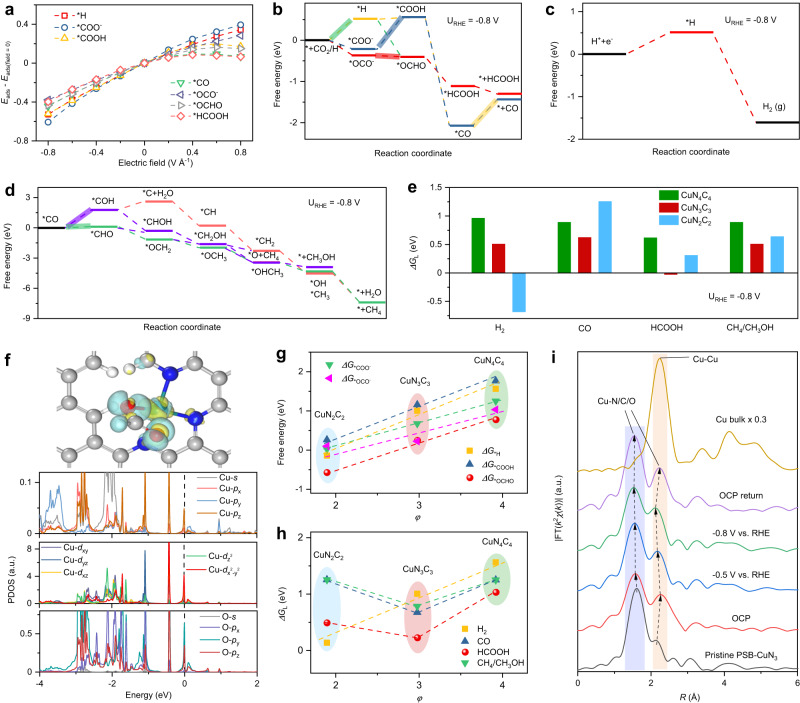
First-principles calculations and operando XAFS characterisation on PSB-CuN_3_ catalysing CO_2_RR. **a** Electric field effects on the adsorbate bindings on the CuN_3_C_3_ structure. **b**–**d** Gibbs free-energy diagrams for CO_2_ electroreduction to CO, HCOOH, H_2_, CH_4_ and CH_3_OH on CuN_3_C_3_ structure. The highlights indicate the potential limiting steps. **e** Magnitudes of the theoretical limiting free-energy differences (*ΔG*_L_) for CO, HCOOH, H_2_ and CH_4_/CH_3_OH productions. **f** Differential charge density and projected density of states for the Cu centre and the bound O atom in the adsorbed *OCHO intermediates supported on CuN_3_C_3_ structure. White, grey, blue, red and cyan balls represent H, C, N, O and Cu atoms, respectively. The black dashed line represents the Femi energy level. **g** Adsorption free energies of ΔG_*OCO_^−^, ΔG_*COO_^−^, ΔG_*OCHO_, ΔG_*COOH_ and ΔG_*H_ vs. the descriptor *φ*. **h** Thermodynamic relations (volcano) lines for the H_2_, CO and HCOOH productions as a function of the descriptor *φ*. **i** Fourier-transformed magnitudes of operando Cu *K*-edge EXAFS spectra for PSB-CuN_3_ recorded at various applied potentials vs. RHE in 0.5 M KHCO_3_ aqueous solution. a.u., arbitrary units.

Because the electric field can interact with intermediates with a substantial dipole moment and/or polarizability^49–51^, we further examined the effects of interfacial electric field and pH on the adsorbate bindings on different CuN_*x*_C_*y*_ structures (Supplementary Table 7). A significant response of the adsorbate bonding strength to the electric field effects is revealed in Fig. 4a and Supplementary Fig. 48. Then, an applied potential of −0.80 V vs. RHE and pH = 7.3 is considered and it leads to a remarkable decrease of the free energy for the adsorbates (Fig. 4b–e, Supplementary Figs. 49 and 50), which indicates more stabilised intermediates and decreased endothermicity for the reaction steps. On the CuN_3_C_3_ structure, the potential limiting steps for formate and CO productions are shifted to ${}^{*}{{{{{\rm{OC}}}}}}{{{{{\rm{O}}}}}}^{-} \to {}^{*}{{{{{\rm{OCHO}}}}}}$ and CO desorption, respectively (Fig. 4b), and it produces a considerably lower limiting free-energy difference (*ΔG*_L_) of −0.03 eV for the formate pathway (Fig. 4e), which is in contrast to the comparable *ΔG*_L_ between the HER (0.51 eV), CO (0.63 eV) and CH_4_/CH_3_OH (0.51 eV) pathways, indicating a strong preference to the formate production. By contrast, a much higher *ΔG*_L_ of 0.62 eV is shown for the formate pathway on the CuN_4_C_4_ structure. This prediction agrees well with the impressively higher FE for CO_2_RR-to-formate on PSB-CuN_3_ catalyst than on PS-CuN_4_ catalyst over similar potential range (see Fig. 3). CuN_2_C_2_ exhibits an extremely strong preference to the HER pathway with a *ΔG*_L_ of −0.68 eV. Similar potential dependence is obtained at an applied potential of −0.40 V vs. RHE (Supplementary Table 7). All in all, the adsorbate energies corrected for the electric field and pH effects strongly support the highest activity and selectivity toward formate in the local symmetry-broken CuN_3_C_3_ moiety.

To understand why the modified electronic structure in PSB-CuN_3_ catalyst is in favour of CO_2_ reduction toward formate, the projected density of states for the adsorbed intermediates of *COO^−^ and *OCO^−^ were obtained. Supplementary Fig. 51 shows the overlap degree between Cu-*d* and O-*p* orbitals is generally larger than that between Cu-*d* and C-*p* orbitals on each CuN_*x*_C_*y*_ structure, thus indicating a stronger binding of *OCO^−^ than *COO^−^. For *OCO^−^, the overlap degree between Cu-*d* and O-*p* orbitals in CuN_3_C_3_ and CuN_2_C_2_ is obviously increased as compared to CuN_4_C_4_. However, the binding of the intermediates in CuN_2_C_2_ is so strong that the potential limiting step shifts to the formate desorption. It suggests that the local symmetry-broken CuN_3_C_3_ can produce a suitable overlap degree between the Cu-*d* and O-*p* orbitals, which can result in a neither strong nor weak binding of the intermediates. Thus, those results unveil that the highest activity and selectivity toward formate in the local symmetry-broken CuN_3_C_3_ moiety is strongly correlated with its unusual coordination environment, in good agreement with the experimentally detected excellent activity of the PSB-CuN_3_ catalyst in electrochemical CO_2_ reduction (Fig. 3).

The intrinsic coordination characteristics of the active CuN_*x*_ centres that dominate the adsorbate binding strength and activity are explored. Breaking of the local *D*_4*h*_ symmetry to create planar-like CuN_3_ and CuN_2_ moieties with lower local *C*_2*v*_ symmetry leads to charge redistribution (Fig. 4f) and increased attractive interactions for the intermediates (Fig. 4b–e). The charge redistribution is first manifested as an obvious upshift of the *d*-band centre of Cu atom in energy relative to the Fermi level from CuN_4_ to CuN_3_ moiety, but this tendency is reversed by a dramatic downshift of the *d*-band centre in CuN_2_ moiety (Supplementary Fig. 52a). This anomaly leads to the widely used *d*-band centre model cannot correlate with the increased adsorbate binding strength and activity (Supplementary Fig. 52b, c), which stems from the enhanced *dsp* orbitals hybridisation near the Fermi level in local symmetry-broken CuN_*x*_ moieties (Supplementary Fig. 53) as also indicated by experimental XANES results. By contrast, the total electron transfer from Cu and the work function appear to be dominated by the coordination number of nearest-neighbour N atoms (denoted as *n*_N_, Supplementary Fig. 54a–c), which matches well with the valence state change obtained by XANES. In addition, approximate linear relationships are observed when *n*_N_ is plotted against adsorption energies and limiting free-energy differences for different intermediates (Supplementary Fig. 54d, e), which suggests that *n*_N_ can be a significant factor of the descriptor^52^. Besides, it is noted that angular distortion has been widely used to describe the stereochemical activity of ns^2^ electron pairs in metal halide perovskites ABX_3_^53^. It suggests that the angle distortion between neighbouring Cu–N bonds in the present work can further modify the charge redistribution and binding affinity, and we add it as an additional factor to modify the descriptor as follows:

1 $$\varphi={n}_{N}\times \left(1-\frac{1}{{n}_{a}}{\sum }_{\left\{i=1\right\}}^{{n}_{a}}\left|\frac{90-{\alpha }_{i}}{90}\right|\right)$$

where *n*_N_ is the number of nearest-neighbour N atoms, *α*_i_ is the N–Cu–N bond angle between neighbouring Cu–N bonds and *n*_*a*_ is the number of the N–Cu–N bond angles. As shown in Fig. 4g, Supplementary Fig. 55 and Supplementary Table 8, excellent linearity is obtained between *φ* and the adsorption energies for *COO^−^, *OCO^−^, *COOH, *OCHO and *H. It means that a decrease in *φ* corresponds to an enhancement in binding strength, which particularly causes a tighter binding of *COO^−^, *OCO^−^, *COOH, *OCHO and *H. This can be attributed to the adoption of a peculiar slant adsorption way (instead of the conventional top-site way, see Fig. 4f) of *OCHO intermediate on the CuN_*x*_ centres with the successive introduction of N vacancy. It leads to an unusual coupling between the in-plane and out-of-plane 3*d*, 4*s* and 4*p* orbitals of the Cu centre and the 2*s* and 2*p* orbitals of the bonded O atom of intermediates (Fig. 4f, Supplementary Figs. 56–58). It suggests that the orbital coupling degree and binding affinity between the Cu centre and the intermediates is directly correlated with the local geometric symmetry breaking of the surrounding N atoms (Supplementary Note 3). Figure 4h shows the theoretical limiting free-energy differences versus the descriptor *φ*. While a good linearity with different slopes is shown for H_2_ production, a flipped volcano relationship is revealed for CO, formate and CH_4_/CH_3_OH productions, with the CuN_3_ moiety lying at bottom of the volcano. The relative order of activity and selectivity from the theoretical prediction reproduces well the experiment data (Fig. 3), thus validating our descriptor *φ*. Hence, the local coordination symmetry breaking, which is quantified as a combination of nearest-neighbour coordination number and bond angle distortion, can serve as a universal descriptor to evaluate the activity of graphene-based single Cu catalysts towards CO_2_RR.

The dynamic processes for CO_2_ adsorption and activation towards highly selective production of formate on PSB-CuN_3_ were further investigated by experimental Cu *K*-edge XAFS spectroscopy under operando conditions (Fig. 4i, Supplementary Figs. 59–66, Supplementary Table 1). The XANES spectra display that the Cu *K*-edge of pristine PSB-CuN_3_ initially shifts to higher energy and the intensity of the shoulder peak D also increases when immersing in CO_2_-saturated 0.5 M KHCO_3_ solution under open-circuit potential (OCP) bias (Supplementary Fig. 59a). This indicates an increased oxidation state for the Cu sites, which is caused by the charge transferred from low-valent Cu^1+^ to CO_2_ molecule to form CO_2_^*δ*+^ species. This assumption is consistent with a slight contraction of the first major peak from 1.60 Å to 1.58 Å as well as an elongation of the second satellite peak from 2.20 Å to 2.26 Å in EXAFS-FT under OCP bias (Fig. 4i). It reflects an additional contribution from the Cu–O bond of the dynamically adsorbed CO_2_ molecular. During CO_2_ reduction at −0.5 V and −0.8 V vs. RHE, the Cu *K*-edge XANES shows a shift back to lower energy without obvious profile change, which indicates the adoption of a low oxidation state for the Cu sites during the catalytic stage, corroborating the high activation ability of CuN_3_C_3_ centres for CO_2_ reduction. Meanwhile, the first two peaks in EXAFS-FT, which now consists of the Cu–N bonds and the Cu–O/C bonds from the reaction intermediates (e.g., *OCO^−^, *OCHO), demonstrates a continuous shift to shorter length and an increase in intensity (Fig. 4i), corresponding to a contraction of Cu–N/O/C bonds with decreased bond disorder. When the applied potential returns to OCP, both XANES and EXAFS-FT reverse to the initial OCP state. Further quantitative EXAFS curve-fitting analysis reveals a decrease in Cu-N/O/C interatomic distances as well as an increase in coordination numbers during the operating process (Supplementary Fig. 63 and Supplementary Table 1). Similar evolution behaviours for the electronic and atomic structures of the metal centres under the catalytic conditions have also been observed in single-Ni-atom catalyst for CO_2_ reduction to CO^54^. Here, the above phenomena consolidate a tighter binding of the reaction intermediates (e.g., *OCO^−^, *OCHO) under the catalytic conditions, which is suggested to be responsible for the high catalytic activity and selectivity of the PSB-CuN_3_.

At the same time, attenuated total reflectance surface-enhanced infra-red absorption spectroscopy (ATR-SEIRAS) was used to probe the reaction intermediates on PSB-CuN_3_ (Supplementary Fig. 67). When the applied potential is more negative than −0.8 V vs. RHE, the obvious peak at ~1620 cm^–1^ exhibits a pronounced rise and red shift, which can be ascribed to the asymmetrical O–C–O stretching band of dissolved formate. Additionally, two broad bands appeared between 1450 and 1350 cm^–1^, and they can be assigned to the symmetrical O–C–O stretching mode of bridge-bonded formate and the C–OH vibration of desorbed formate molecules, respectively^55^. No signal of *COO^−^/*COOH was detected. These findings evidence the preferred formation of *OCO^−^/*OCHO intermediates bonded to the PSB-CuN_3_ catalyst via O.

The impact on the catalytic and structural stability from N coordination number variation is investigated (See detailed discussion in Supplementary Note 4). While PSB-CuN_3_ shows better electrocatalytic stability than PS-CuN_4_ in both H-cell and flow cell under the same applied potential, the detailed operando and postmortem XAFS with AC-HAADF-STEM characterisations revealed a structural reconstruction occurs on both PSB-CuN_3_ and PS-CuN_4_ during CO_2_ reduction. Compared with PS-CuN_4_ with high valence Cu^2+^, the structure change of low-valent Cu^1+^ PSB-CuN_3_ is slight. And, the structural evolution process of PSB-CuN_3_ is reversible and no atoms aggregation can be observed after CO_2_ electrocatalysis. On contrary, the dramatic structural reconstruction of PS-CuN_4_ leads to an irreversible aggregation of fractional Cu atoms. Therefore, the electrochemical stability of PSB-CuN_3_ is better than that of PS-CuN_4_, consistent with previous works that the low-valent Ni^1+^ single-atom catalysts showed no reconstruction into nanoparticles for CO_2_RR to CO^54^, but the high-valent Cu^2+^ single-atom catalysts reconstructed to nanoparticles in the electrochemical reduction of nitrate to ammonia^56^ and electrocatalytic oxygen reduction^57^.

In summary, the local planar-symmetry-broken PSB-CuN_3_ is successfully prepared by a simple and facile method, and further used as the electrocatalysts for CO_2_RR. Significantly, the as-prepared PSB-CuN_3_ catalyst exhibits extremely high electrocatalytic activity and catalytic stability in both H-cell and flow cell systems, superior to the highly symmetrical PS-CuN_4_ and comparable to recently best-reported CO2RR-to-HCOO^−^ catalysts. By combining ex-situ/operando EXAFS, XANES and ATR-SEIRAS analyses with theoretical calculations, we unambiguously unveiled that the high catalytic performance for formate production originates from the intrinsic local symmetry breaking from the planar-like *D*_4*h*_ to *C*_2*v*_ configuration in PSB-CuN_3_, such that an unconventional *dsp* hybridisation is emerged. With this anomaly, the catalytic activities are unconformable to the widely used *d*-band centre theory, whereas the local environment of the metal centre including coordination number and geometric distortion is identified as a newly universal descriptor to the intermediate adsorption and thus catalytic activities for H_2_/CO/HCOOH/CH_4_/CH_3_OH productions in graphene-based SACs. Our study provides a universal and efficient guideline for the rational design and accurate modulation of the active centre’s local symmetry in nonprecious metal SACs for diverse CO_2_RR applications at the atomic scale.

## Methods

### Chemicals

CuCl_2_·6H_2_O (99.99% AR grade) was bought from Alfa Aesar. Nafion solution (5 wt%), natural graphite flakes (100 mesh), ammonia solution (~30%) and KHCO_3_ (99.95%) were obtained from Sigma Aldrich. All chemicals were used directly without further purification. The ultrapure water (18 MΩ) used in the experiments was supplied by a Millipore System (Millipore Q).

### Synthesis of PSB-CuN_3_ and PS-CuN_4_

Graphene oxide (GO) was first synthesised by a modified Hummers’ method^58^. Then, NH_4_OH solution (0.5 mL) was added dropwise to a GO aqueous suspension (5 mL, 2 mg mL^−1^) and stirred for 20 min. The mixed suspension was transferred to a Teflon vessel at 70 °C for 5 h under airtight conditions. After cooling and six times washing using water, the purified AGNs were obtained. Subsequently, 80 μL CuCl_2_·6H_2_O (3 mg mL^−1^) was added to the well-dispersed AGNs solution under stirring for 1 h. The purified samples were subsequently transferred into a freeze-drying vessel, which had been set at the temperature of −53 °C and vacuum pressure of −30 Pa. After being freeze-dried, the Cu^2+^-containing AGNs, together with small amounts (≈1 wt%) of thermally reduced (300 °C for 1 h in Ar) graphene as catalysts, were sealed in an Ar gas-protected glass vial and then undergo a microwave process (microwave digestion workstation XH-800C) under 1000 W for 5 s^59^. PSB-CuN_3_ was collected after a cooling period for 20 min. For comparison, PB-CuN_4_ was synthesised by the pyrolysis of Cu^2+^-containing AGNs in an Ar/NH_3_ mixed atmosphere according to the reported method^17^.

### Physical characterisations

XRD patterns were collected by using a Bruker D8 Advance X-ray powder diffractometer with a Cu *K*_α_ irradiation source (λ = 1.5406 Å), and a self-calibration process was performed with a SiO_2_ internal standard sample prior to target measurement. The sizes and morphologies of the samples were first recorded using a Hitachi H-800 transmission electron microscope. Then, the high-resolution STEM and elemental mappings were carried out on a JEOL JEM-2100F with an electron acceleration energy of 200 kV. A JEM-ARM200F transmission electron microscope operated at 200 keV, equipped with a probe spherical aberration corrector, was used to obtain HAADF-STEM images of the samples. All TEM/STEM samples were prepared by depositing a tiny amount of the samples onto a molybdenum (Mo) grid. Quantitative analysis of metal loading was carried out using inductively coupled plasma atomic emission spectroscopy (TJA RADIAL IRIS 1000 ICP-AES). XPS spectra were performed by a Thermo Scientific K-Alpha spectrometer using a monochromatic Al *K*_α_ radiation, where the analysis chamber was 1.5 × 10^−9^ mbar and the X-ray spot was ~500 μm. The C 1 s peak for adventitious hydrocarbons at 284.8 eV was used for binding energy calibration. Raman measurements were conducted on a Renishaw Microscope System RM2000 with a Renishaw spectrometer of 532 nm. The N_2_ adsorption/desorption curve was performed at 77 K using a Quadrasorb SI (Quantachrome, USA) surface area analyser.

### Electrochemical measurements

The electrochemical measurements were performed on a CHI 760e electrochemical workstation in a three-electrode configuration cell using an as-prepared electrode as the working electrode, platinum wire as the counter electrode and Ag/AgCl (saturated KCl) as the reference electrode in 0.5 M KHCO_3_ aqueous electrolyte. Nafion117 membrane was inserted between the cathodic chamber and anodic chamber of H-type cell. The catalyst inks were prepared by a mixture of catalyst (5 mg), water (240 μL), isopropanol (750 μL) and 5% Nafion solution (10 μL), followed by ultrasonication for 4 h. An aliquot of 0.1 mL of the catalyst ink was deposited onto carbon paper (HCP120, Shanghai Hesen Electric Co., Ltd) and allowed to dry in air, giving a catalyst loading of 0.5 mg cm^−2^. Before the measurement, the 0.5 M KHCO_3_ aqueous solution (pH = 7.3) was purged by bubbling Ar for 30 min and then switched to CO_2_ until saturation. The LSV curves were conducted at a scan rate of 10 mV/s with continuous bubbling CO_2_. For comparison, cyclic voltammetry curves tested in an Ar (99.99%)-saturated electrolyte were also obtained. All potentials were converted to the RHE scale according to the standard Nernst equation (${{{{{{\rm{E}}}}}}}_{{{{{{\rm{RHE}}}}}}}={{{{{{\rm{E}}}}}}}_{{{{{{\rm{Ag}}}}}}/{{{{{\rm{AgCl}}}}}}}+0.0591\times {{{{{\rm{pH}}}}}}+0.197{{{{{\rm{V}}}}}}$). All current densities were normalised to the geometrical area of electrode (1 × 1 cm^2^). The gas products of CO_2_RR were checked by online gas chromatography (GC, Supplementary Fig. 33). The ^1^H NMR spectroscopy was used to analyse and quantify the products in the liquid phase (Supplementary Fig. 34). Samples were prepared by mixing DMSO and electrolyte aliquots. All the potentials were reported vs. RHE and manually corrected by 100% *iR* compensation, which was obtained through electrochemical impedance spectroscopy (Supplementary Fig. 30 and Supplementary Table 9). EIS measurements were conducted at 25 °C using an H-type cell (consisting of two independent anode and cathode chambers separated by a nafion membrane) and a CHI 760e electrochemical workstation. Platinum wire served as the counter electrode, while Ag/AgCl (saturated with KCl) was employed as the reference electrode. The EIS measurement was carried out under open-circuit voltage, and the frequency setting range was from low frequency 1 Hz to high frequency 100,000 Hz. The amplitude was set as 0.005 V. For the flow cell study, catalyst inks were air-brushed onto a gas diffusion layer (GDL) as the cathode electrode with a mass loading of ~0.6 mg cm^−2^. IrO_2_ was used as the counter electrode. The IrO_2_ catalyst ink was prepared by a mixture of commercial IrO_2_ powder (5 mg), water (240 μL), isopropanol (750 μL) and 5% Nafion solution (10 μL), followed by ultrasonication for 4 h. Then, IrO_2_ catalyst (0.7 mg/cm^2^) was loaded onto the GDL as anode. Electrolyte was pumped by a syringe pump (PHD 2000, Harvard Apparatus) over the cathode GDL at a constant flow rate of 10 standard cubic centimetres per minute (sccm). High-purity CO_2_ gas flowed at a rate of 30 sccm behind the cathode GDL controlled by a mass flow controller (CS200, Beijing Sevenstar flow). Polarisation curves of all studied electrodes are performed at a sweeping potential between 0 and −1.2 V vs. RHE (all potentials reported here were based on RHE) with a scan rate of 10 mV s^−1^.

### Gas product analysis

Gas products from the cathodic compartment during the electrochemical reactions were analysed using a GC-2014 (Shimadzu) equipped with a BID detector and ShinCarbon ST100/120 packed column. High-purity helium (99.9999%) was used as the carrier gas. The FEs of the gas products were calculated by using the concentrations (ppm) detected by the GC as follows (CO products are selected as an example):

2 $${{{{{\rm{F}}}}}}{{{{{{\rm{E}}}}}}}_{{{{{{\rm{CO}}}}}}}(\%)=\frac{{Q}_{{{{{{\rm{CO}}}}}}}}{{{{{{{\rm{Q}}}}}}}_{{{{{{\rm{total}}}}}}}}\times 100\%=\frac{\left(\frac{\nu }{60{{{{{\rm{s}}}}}}/\min }\right) \times \left(\frac{y}{22400{{{{{\rm{c}}}}}}{{{{{{\rm{m}}}}}}}^{3}/{{\rm{mol}}}}\right)\times N\times F\times 100\%}{{{{j}}}}$$

where *ν* is gas flow rate measured by a flowmeter at the exit of the cell at room temperature, *y* is the measured volume concentration of product, *N* is the number of electrons required to form a molecule of gas product, F and *j* are the Faraday constant (96,500 C mol^−1^) and the recorded current, respectively.

### Liquid product analysis

Liquid products were analysed by using a 400 MHz NMR spectrometer (Bruker), with DMSO as internal standards. The calibration curve was made by measuring standard solutions of formate. The FEs of liquid products were calculated as follows:

3 $${{{{{\rm{F}}}}}}{{{{{{\rm{E}}}}}}}_{{{{{{\rm{HCO}}}}}}{{{{{{\rm{O}}}}}}}^{-}}(\%)=\frac{{Q}_{{{{{{\rm{HCO}}}}}}{{{{{{\rm{O}}}}}}}^{-}}}{{Q}_{{{{{{\rm{total}}}}}}}}\times 100\%=\frac{{n}_{{{{{{\rm{HCO}}}}}}{{{{{{\rm{O}}}}}}}^{-}\times N\times F\times 100\%}}{j\times t}$$

where *n*_HCOO−_ is the measured amount of formate in the cathodic compartment and *t* is the reaction time. The HCOO^−^ partial current density at different potentials was calculated by multiplying the overall geometric current density and its corresponding FE.

Turnover Frequency (TOF, h^−1^) for the CO_2_ electroredution to formate was evaluated based on the 2-electron pathway:

4 $${{{{{\rm{TOF}}}}}}=\frac{{I}_{{{{{{\rm{product}}}}}}}/{NF}}{{m}_{{{{{{\rm{cat}}}}}}}\times w/{M}_{{{{{{\rm{metal}}}}}}}}\times 3600$$

where *I*_product_ is the partial current for formate, *N* is the number of electrons transferred for product formation, *F* is the Faraday constant (96,500 C mol^−1^), *m*_cat_ is the catalyst mass in the electrode, *w* is the metal loading in the catalyst and *M*_metal_ is the atomic mass of Cu (63.5 g mol^−1^).

### Soft XAS measurements

Soft C, N *K*-edge and Cu *L*_3,2_-edge X-ray absorption spectra were measured at BL12B station of Hefei National Synchrotron Radiation Laboratory in the total electron yield mode by collecting the sample drain current under a vacuum better than 10^−7^ Pa.

### Ex-situ and operando XAFS measurements

Hard X-ray absorption spectra at Cu *K*-edge were acquired under ambient conditions in fluorescence mode using Si(111) double-crystal monochromators at 1W1B and 4B9A beamlines of Beijing Synchrotron Radiation Facility (BSRF, operated at 2.5 GeV with a maximum current of 250 mA). The incident and fluorescence X-ray intensities were monitored by using standard N_2_-filled ion chambers and air-filled Lytle-type detector, respectively.

A home-made cell collected to a computer-controlled electrochemical analyser was used for the operando XAFS experiments, which were operated under identical conditions as the electrochemical measurements. A catalyst-modified carbon paper was used as working electrode, Pt wire as counter electrode and Ag/AgCl (KCl-saturated) electrode as the reference electrode. CO_2_-saturated 0.5 M aqueous KHCO_3_ was used as electrolytes. Operando XAFS spectra were recorded at different potentials to monitor the structural evolutions of the catalysts.

### XAFS data analysis

The XAFS raw data were background-subtracted, normalised and Fourier-transformed by the standard procedures with the ATHENA programme^44,60^, and least-squares curve-fitting analysis of the EXAFS *χ*(k) data was carried out using the ARTEMIS programme based on the standard EXAFS equation. All fits were performed in the *R* space with *k*-weight of 2. The best-fit results are shown in Supplementary Figs. 13–17 with the fitting parameter values listed in Supplementary Table 1.

The Cu *K*- and *L*-edge theoretical XANES calculations were carried out with the FDMNES code in the framework of real-space full multiple-scattering (FMS) scheme using Muffin-tin approximation for the potential^43,61,62^. The energy-dependent exchange-correlation potential was calculated in the real Hedin–Lundqvist scheme, and then the spectra was convoluted using a Lorentzian function with an energy-dependent width to account for the broadening due both to the core–hole width and to the final state width. The CuN_*x*_C_*y*_ moieties were built based on the graphene-enclosed MN_4_C_4_ structural motif (Supplementary Fig. 21), which can be regarded as a single Cu atom occupying the divacancy in the graphene lattice with coordination to four pyridinic Ns included in six-member rings. Satisfactory convergence for the cluster sizes had been achieved.

### Operando ATR-SEIRAS

Operando ATR-SEIRAS was performed on a Nicolet iS20 spectrometer equipped with a VeeMax III (PIKE technologies) accessory and an HgCdTe (MCT) detector cooled with liquid nitrogen. The electrochemical test was conducted in a custom-made three-electrode electrochemical single cell. A Pt wire and a saturated Ag/AgCl were used as the counter and reference electrodes, respectively. A fixed-angel Si prism (60°) coated with Au thin layer was used to load catalysts and serve as the working electrode. The operando ATR-SEIRAS spectra were recorded by varying the potential in CO_2_-saturated 0.50 M KHCO_3_. The spectrum recorded at the initial potential of 0 V was used for the background subtraction.

### Computational method and models

The first-principles calculations including structural and electronic performances were carried out in the framework of density functional theory (DFT) as implemented in the Cambridge Sequential Total Energy Package (CASTEP)^63^. The exchange-correlation functional under the generalised gradient approximation (GGA) with norm-conserving pseudopotentials and Perdew–Burke–Ernzerhof functional was adopted to describe the electron-electron interaction^64^. The spin polarisation was considered^52^. The DFT-D2 method of Grimme for the Van der Waals correction was used^65^. An energy cutoff of 750 eV and a *k*-point sampling set of 5 × 5 × 1 were tested to be converged. A force tolerance of 0.01 eV Å^−1^, energy tolerance of 5.0 × 10^−7 ^eV per atom and maximum displacement of 5.0 × 10^−4 ^Å were adopted. The surfaces of the structures were built with a vacuum space of 20 Å along the z-direction, which is enough to avoid interaction between the two neighbouring images. The effect of water was considered by using the implicit solvent model with the dielectric constant of 78.54 for water molecule. The structure models were built with a vacuum space of 20 Å along the z-direction, which is enough to avoid interaction between the two neighbouring images. Then the intermediates for CO, HCOOH, H_2_, CH_4_, CH_3_OH and C_2_ products were adsorbed on the active centres. All the atoms were relaxed to release the internal stress of systems. In addition, an electric field was applied to consider its effect on the binding geometry and energy of the adsorbates.

The formation energy of the active centres was estimated by:

5 $$\Delta {E}_{{{{{{\rm{f}}}}}}}={E}_{{{{{{\rm{sys}}}}}}}-{n}_{{{{{{\rm{C}}}}}}}*{\mu }_{{{{{{\rm{C}}}}}}}-{n}_{{{{{{\rm{N}}}}}}}*{\mu }_{{{{{{\rm{N}}}}}}}-{n}_{{{{{{\rm{H}}}}}}}*{\mu }_{{{{{{\rm{H}}}}}}}-{\mu }_{C{{{{{\rm{u}}}}}}}$$

where *E*_sys_ was the total energy of each system; n_C_,n_N_ and n_H_ denoted the amounts of C, N and H atoms in each system; μ_C_, μ_N_ and μ_H_ were the chemical potentials of C, N and H atoms, denoting the energy of per C, N and H atom in graphite, N_2_ gas and H_2_ gas.

The adsorption energy Δ*E*_ads_ of each intermediate on the surface of substrates was defined as:

6 $$\Delta {E}_{{{{{{\rm{ads}}}}}}}={E}_{{{{{{{\rm{inter}}}}}}}^{*}}-({E}_{{}^{*}}+{E}_{{{{{{\rm{inter}}}}}}})$$

where the subscripts inter* and * denoted the adsorption of intermediate on substrates and the bare substrates, *E*_inter_ denoted the energy of intermediate.

The Gibbs free energy (Δ*G*) of each chemical reaction without external electric field (i.e., *E* = 0) was calculated by:

7 $$\Delta {G}_{{{ads}}^{E=0}}=\Delta {E}_{{{ads}}^{E=0}}+\Delta {ZPE}-T\Delta S$$

where Δ*E*_ads_, Δ*ZPE*, *T* and Δ*S* denoted the calculated total energy, zero point energy, temperature and entropy, respectively. Then, the energy was corrected for the RHE scale and electric field by^49,50^:

8 $$\Delta {G}_{{ads}}=\Delta {G}_{{{ads}}^{E=0}}+\mu E-\frac{\alpha {E}^{2}}{2}-{ne}{U}_{{RHE}}$$

where *E* was the external electric field, *ne* was the amount of proton–electron pairs transferred in each chemical reaction, *U*_*RHE*_ was the applied potential vs. RHE. The values of *μ and α* were acquired from the second-order polynomial fitting for the energy of each adsorbate across the range of electric fields (see Supplementary Fig. 48). The external electric field was pH-dependent on an RHE scale and estimated by:

9 $$E={C}_{H}\left(\right.{U}_{{RHE}}-{\kappa }_{B}T \, {{{{\mathrm{ln}}}}}\left(10\right)\times {pH}-{U}_{{PZC}}/{{{{{{\rm{\epsilon }}}}}}{{{{{\rm{\epsilon }}}}}}}_{0}$$

where *C*_*H*_ = 20 μF cm^−2^ refers to Helmholtz capacitance for archetypical carbon material^66^, ϵ_0_ refers to vacuum permittivity (8.85 ×10^−12 ^F m^−1^), *ϵ* = 2 refers to dielectric constant of water near a surface (unitless), *k*_B_ is the Boltzmann constant, *T* = 300 K and pH = 7.3 is kept consistent with experimental conditions, *U*_*PZC*_ = 0.22 V refers to the potential at the point of zero charge (PZC) vs. SHE, which was measured by differential capacitance curves using impedance methods^67^.

Five electrochemical reaction pathways were considered^47,48,68^:

(I) For formate production,
  10 $${}^{*}+{{{{{\rm{C}}}}}}{{{{{{\rm{O}}}}}}}_{2}+{{{{{{\rm{e}}}}}}}^{-}\to {}^{*}{{{{{\rm{O}}}}}}^{-}{{{{{\rm{OC}}}}}}$$
  11 $${}^{*}{{{{{\rm{OC}}}}}}{{{{{{\rm{O}}}}}}}^{-}+{{{{{{\rm{H}}}}}}}^{+}\to {}^{*}{{{{{\rm{OCHO}}}}}}$$
  12 $${}^{*}{{{{{\rm{OCHO}}}}}}+({{{{{{\rm{H}}}}}}}^{+}+{e}^{-})\to {}^{*}{{{{{\rm{HCOOH}}}}}}$$
  13 $${}^{*}{{{{{\rm{HCOOH}}}}}}\to {}^{*}\,+{{{{{\rm{HCOOH}}}}}}$$
(II) For formate production,
  14 $${}^{*}+({{{{{{\rm{H}}}}}}}^{+}+{e}^{-})\to {}^{*}{{{{{\rm{H}}}}}}$$
  15 $${}^{*}{{{{{\rm{H}}}}}}+{{{{{{\rm{CO}}}}}}}_{2}\to {}^{*}{{{{{\rm{OCHO}}}}}}$$
  16 $${}^{*}{{{{{\rm{OCHO}}}}}}+({{{{{{\rm{H}}}}}}}^{+}+{e}^{-})\to {}^{*}{{{{{\rm{HCOOH}}}}}}$$
  17 $${}^{*}{{{{{\rm{HCOOH}}}}}}\to {}^{*}\,+{{{{{\rm{HCOOH}}}}}}$$
(III) For CO production,
  18 $${}^{*}+{{{{{\rm{C}}}}}}{{{{{{\rm{O}}}}}}}_{2}+{{{{{{\rm{e}}}}}}}^{-}\to {}^{*}{{{{{\rm{O}}}}}}^{-}{{{{{\rm{CO}}}}}}$$
  19 $${}^{*}{{{{{\rm{O}}}}}}^{-}{{{{{\rm{CO}}}}}}+{{{{{{\rm{H}}}}}}}^{+}\to {}^{*}{{{{{\rm{COOH}}}}}}$$
  20 $${}^{*}{{{{{\rm{COOH}}}}}}+\left({{{{{{\rm{H}}}}}}}^{+}+{{{{{{\rm{e}}}}}}}^{-}\right)\to {}^{*}{{{{{\rm{CO}}}}}}+{{{{{{\rm{H}}}}}}}_{2}{{{{{\rm{O}}}}}}$$
  21 $${}^{*}{{{{{\rm{CO}}}}}}\to {}^{*}\,+{{{{{\rm{CO}}}}}}$$
(IV) For CO production,
  22 $${}^{*}+({{{{{{\rm{H}}}}}}}^{+}+{e}^{-})\to {}^{*}{{{{{\rm{H}}}}}}$$
  23 $${}^{*}{{{{{\rm{H}}}}}}+{{{{{{\rm{CO}}}}}}}_{2}\to {}^{*}{{{{{\rm{COOH}}}}}}$$
  24 $${}^{*}{{{{{\rm{COOH}}}}}}+\left({{{{{{\rm{H}}}}}}}^{+}+{e}^{-}\right)\to {}^{*}{{{{{\rm{CO}}}}}}+{{{{{{\rm{H}}}}}}}_{2}{{{{{\rm{O}}}}}}$$
  25 $${}^{*}{{{{{\rm{CO}}}}}}\to {}^{*}\,+{{{{{\rm{CO}}}}}}$$
(V) For hydrogen evolution reaction,

26 $${}^{*}+({{{{{{\rm{H}}}}}}}^{+}+{e}^{-})\to {}^{*}{{{{{\rm{H}}}}}}$$

27 $${}^{*}+({{{{{{\rm{H}}}}}}}^{+}+{e}^{-})\to {}^{*}{{{{{\rm{H}}}}}}$$

The electrochemical reaction steps for CH_4_, CH_3_OH and C_2_ productions were referred to in the published works^48,60^.

### Supplementary information

Supplementary Information

## Data Availability

Data availability Full data supporting the findings of this study are available within the article and its Supplementary Information, as well as from the corresponding author on request.

## Code availability

The codes that support the findings of this study are available from the corresponding author on request.
