# Supplementary material for: Continuous electroproduction of formate via CO2 reduction on local symmetry-broken single-atom catalysts
Source: Nat Commun. 2023 Oct 27;14:6849. doi: 10.1038/s41467-023-42539-1 (PMC10611760; doi:10.1038/s41467-023-42539-1)
Supplement: Supplementary file 1 — Supplementary Information [file 41467_2023_42539_MOESM1_ESM.pdf]

Supplementary Information for

Continuous electroproduction of formate via CO_2 reduction on local symmetry-broken single-atom catalysts

Juncai Dong[1,#,*], Yangyang Liu[2,3,#], Jiajing Pei[1,#], Haijing Li[1], Shufang Ji[4], Lei Shi[2], Yaning Zhang[1], Can Li[5*], Cheng Tang[6], Jiangwen Liao[1], Shiqing Xu[5], Huabin Zhang[7], Qi Li[2] and Shenlong Zhao[2,3*]

Contents:

Supplementary Figures 1 to 67

Supplementary Tables 1 to 9

Supplementary Notes 1 to 4

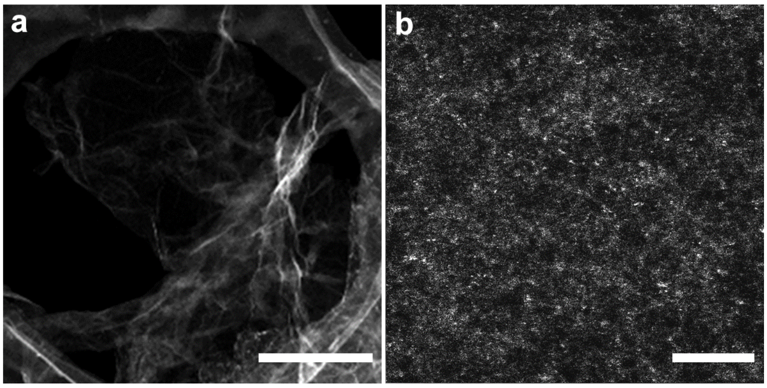

Supplementary Fig. 1. **a**,**b**, HADDF-STEM image (scale bar, 1 μm; **a**) and AC HAADF-STEM image (scale bar, 5 nm; **b**) of PSB-CuN_3.

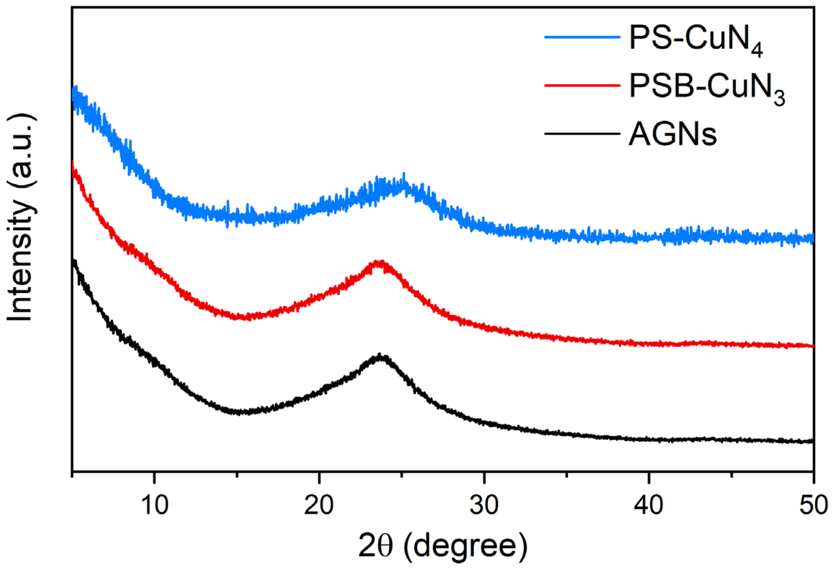

Supplementary Fig. 2. XRD patterns of AGNs, PSB-CuN_3 and PS-CuN_4. XRD patterns of AGNs, PSB-CuN_3 and PS-CuN_4 demonstrated a broadened peak at the range of 20-30° that is assigned to the (002) plane of the graphitic carbon. Further, no signals of Cu-derived nanoparticles were observed, excluding obvious aggregation of the Cu species.

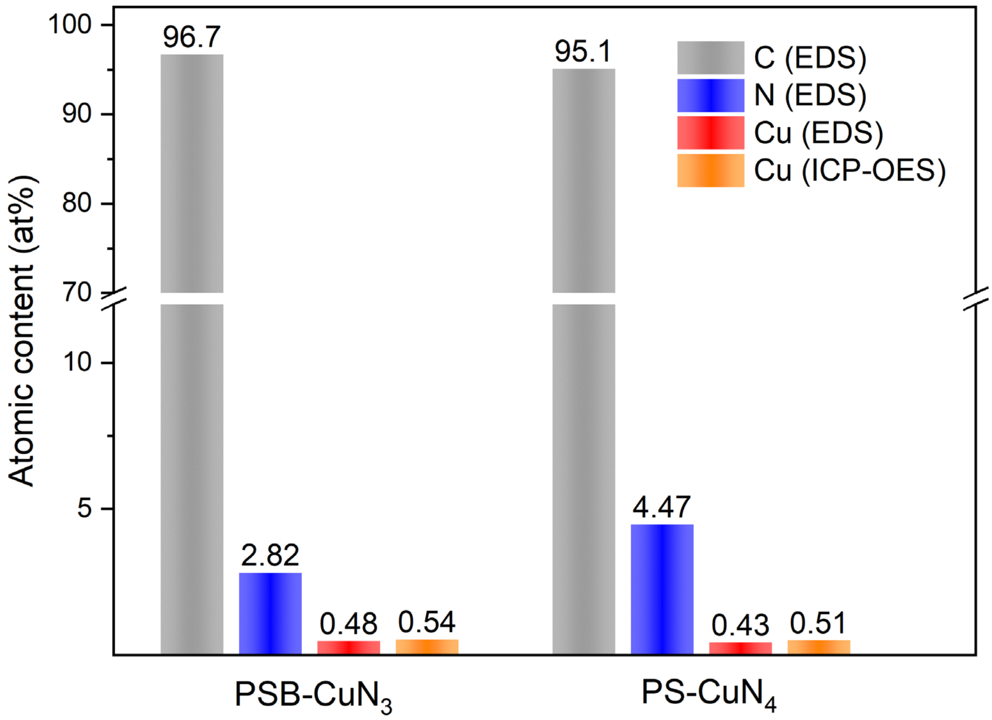

Supplementary Fig. 3. Comparison of the atomic content percentages of C, N and Cu in PSB-CuN_3 and PS-CuN_4 measured by STEM-EDS analysis, together with the ICP-OES results. Composition analysis by STEM-EDS indicates that as-obtained PSB-CuN_3 are composed of C, N, and Cu with average content 96.7 at %, 2.82 at %, and 0.48 at %, respectively, which is similar to that of PS-CuN_4 (Supplementary Fig. 10). Also, quantitative measurement by inductively coupled plasma-mass spectrometry (ICP-OES) reveals that Cu content in PSB-CuN_3 and PS-CuN_4 are 0.54 at% and 0.51 at%, respectively.

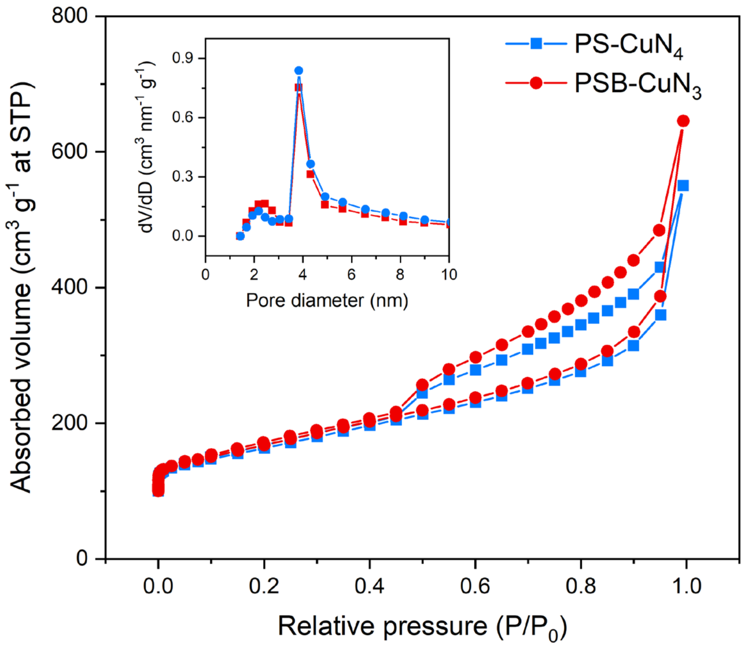

Supplementary Fig. 4. N_2 adsorption-desorption isotherms at 77 K up to 1 bar and the pore size distribution (the inset) of PSB-CuN_3 and PS-CuN_4. N_2 adsorption-desorption isotherms of the sample show a comparable BET surface area of ~ 510 m^2g^{-1} with the dominant pore size of ~ 4 nm (Supplementary Fig. 4), benefitting to the mass transfer and diffusion during electrocatalysis. The samples were degassed at 300 ℃ for 8 h before adsorption measurements.

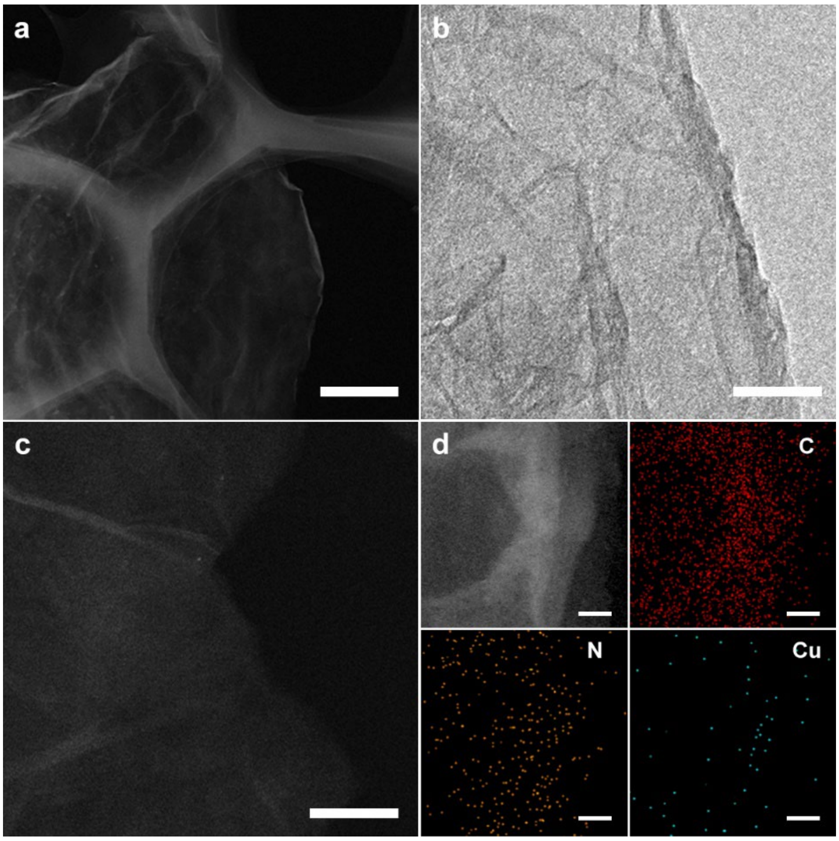

Supplementary Fig. 5. a-d, HADDF-STEM image (scale bar, 1 μm; **a**), TEM image (scale bar, 200 nm; **b**), HAADF-STEM image (scale bar, 200 nm; **c**) and corresponding EDS mapping images (C, red; N, orange; Cu, blue; scale bar, 10 nm; **d**) of PS-CuN_4. It shows that the as-prepared PS-CuN_4 possesses the layered structure and no aggregates on its surface.

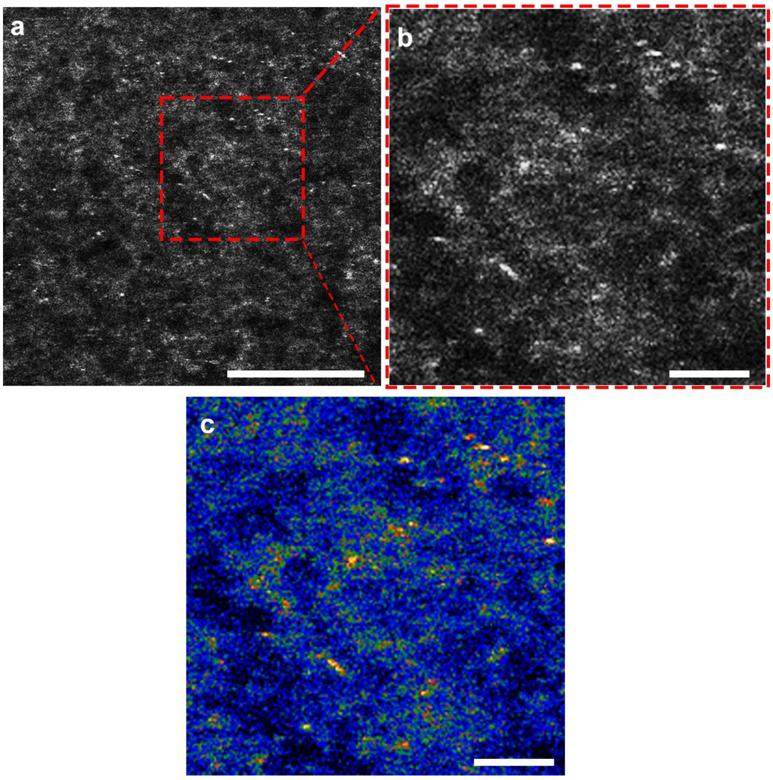

Supplementary Fig. 6. a-c, AC-HAADF-STEM image (scale bar, 5 nm; **a**), enlarged image (scale bar, 1 nm; **b**) and corresponding intensity maps (scale bar, 1 nm; **c**) of PS-CuN_4. The atomically dispersed Cu centres can be observed in PS-CuN_4 by AC-HAADF-STEM analysis (Supplementary Fig. 6a). These active sites are clearly identified by several separated bright dots under a high magnification mode and corresponding intensity maps (Supplementary Fig. 6b,c).

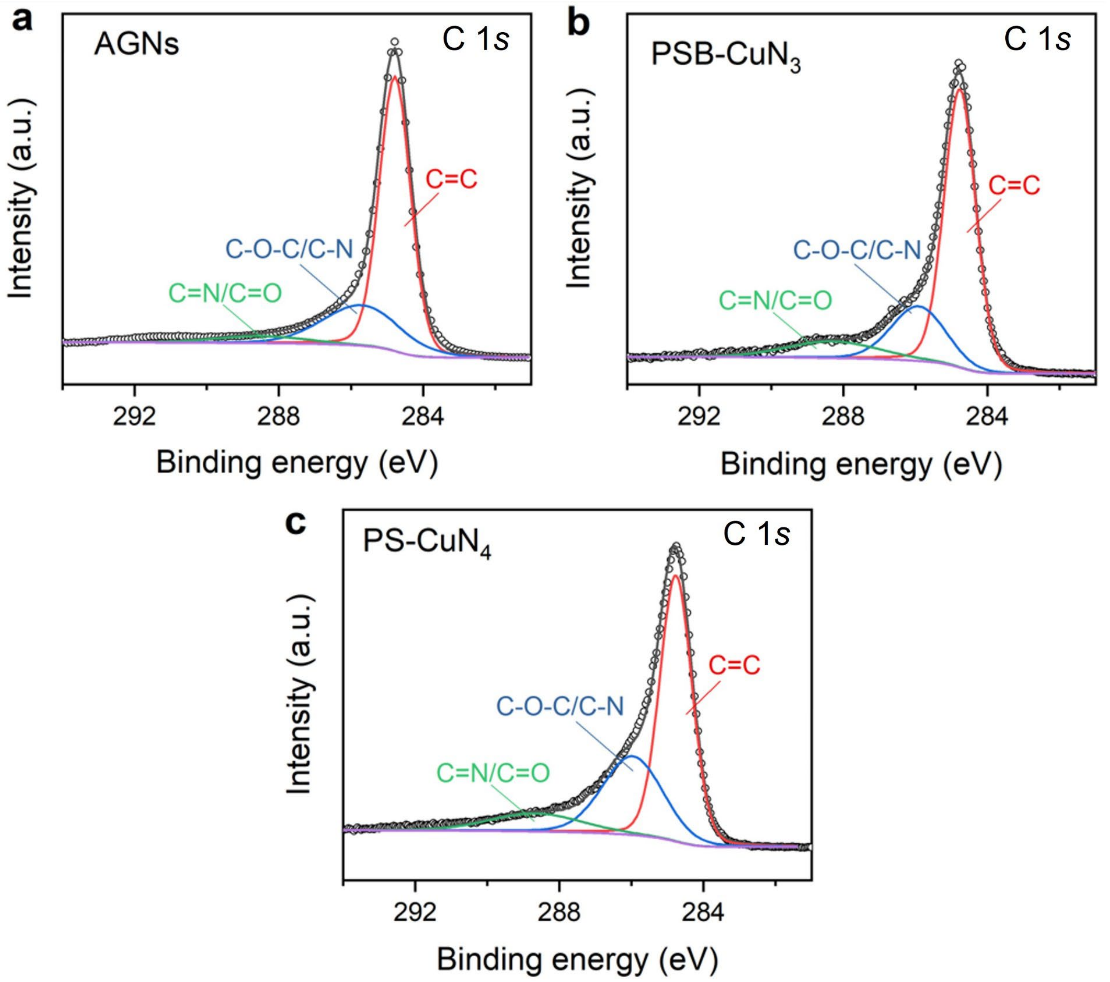

Supplementary Fig. 7. **a**-**c**, C 1*s* XPS spectra of AGNs (**a**), PSB-CuN_3 (**b**) and PS-CuN_4 (**c**). C 1*s* spectrum can be deconvoluted into three peaks with binding energies of 284.8, 285.9 and 288.3 eV, which can be ascribed to graphitic C=C, C-N/C-O-C and C=N/C=O respectively, with the former two being the dominant species.

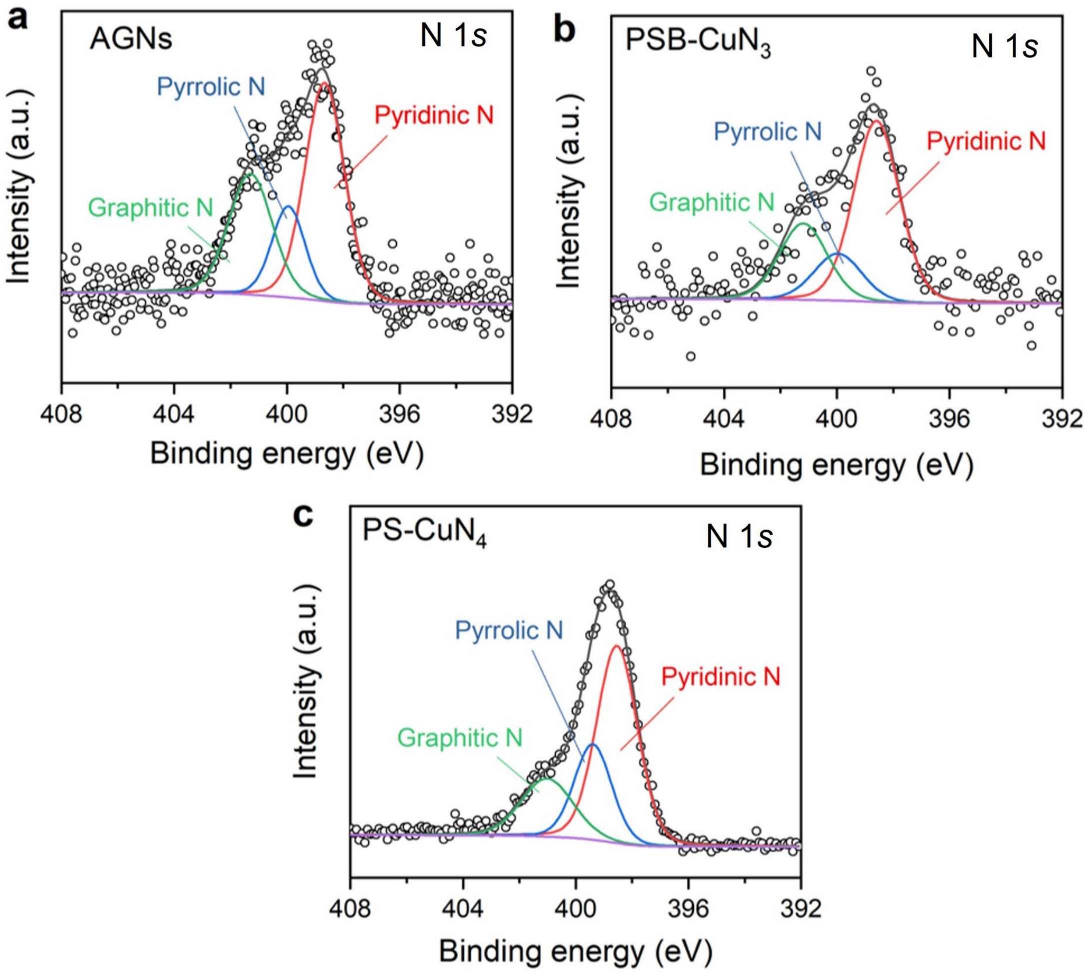

Supplementary Fig. 8. a-c, N 1*s* XPS spectra of AGNs (**a**), PSB-CuN_3 (**b**) and PS-CuN_4 (**c**). N 1*s* spectrum shows the pyridinic (398.6 eV), pyrrolic (399.4 eV) and graphitic N (401.2 eV) are the dominant species.

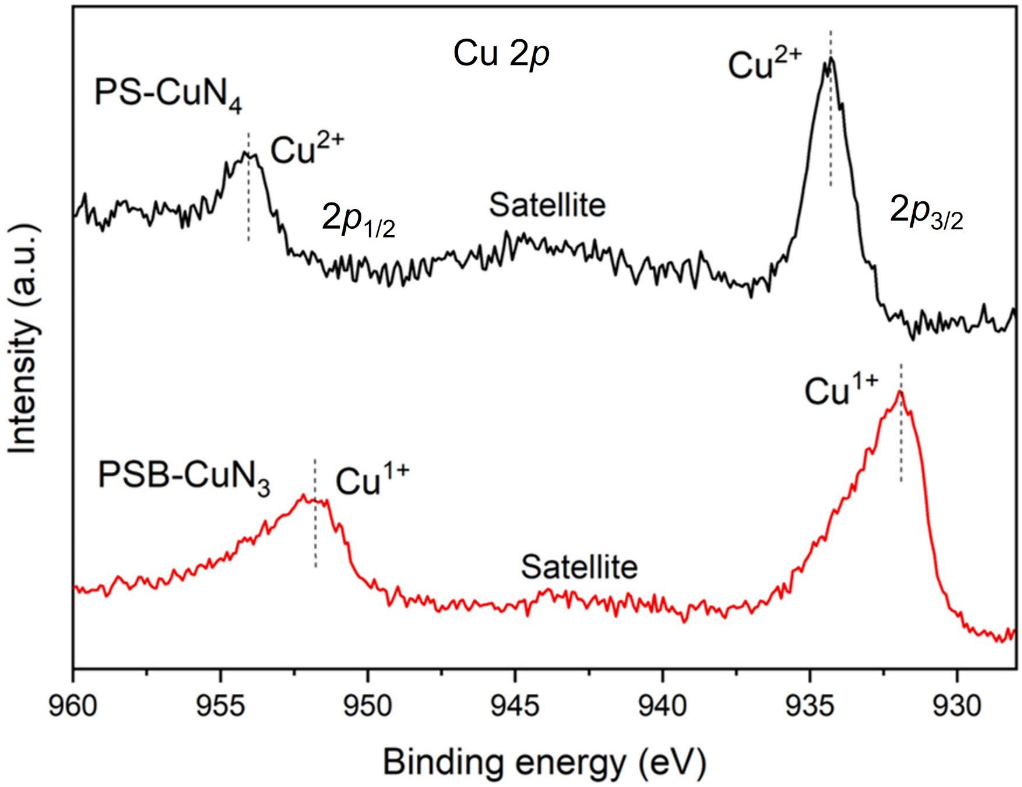

Supplementary Fig. 9. Cu 2*p* XPS spectrum of PSB-CuN_3 and PS-CuN_4. From Cu 2*p* spectrum, it can be observed that PSB-CuN_3 is dominated by Cu^{1+} species (Cu $2p_{3/2}$ and $2p_{1/2}$ peaks are located at 931.9 eV and 951.8 eV, respectively), while PS-CuN_4 is dominated by Cu^{2+} species (Cu $2p_{3/2}$ and $2p_{1/2}$ peaks are located at 934.3 eV and 953.9 eV respectively), indicating a lower valence state of Cu^{1+} in PSB-CuN_3.

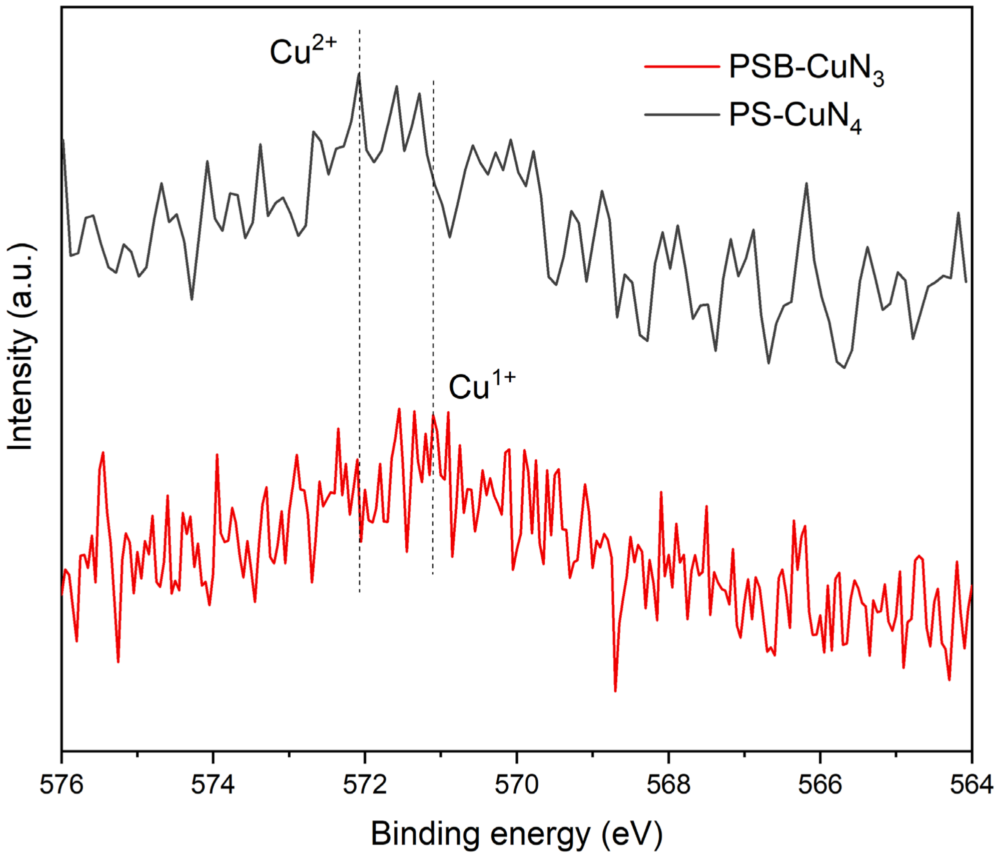

Supplementary Fig. 10. The Cu LMM XPS spectra for PSB-CuN_3 and PS-CuN_4 catalysts.

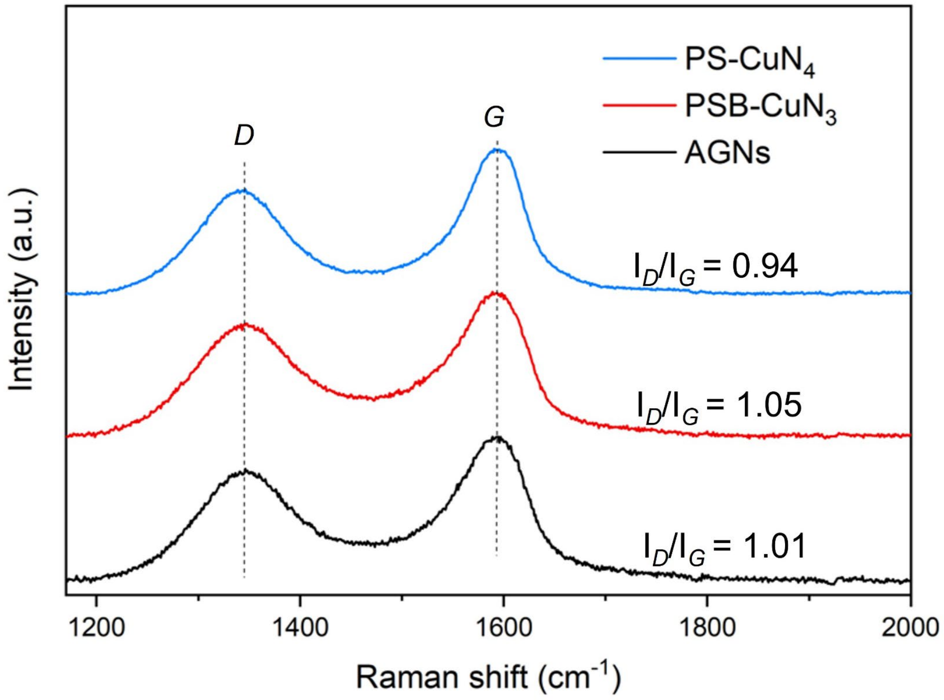

Supplementary Fig. 11. Raman spectra of AGNs, PSB-CuN_3 and PS-CuN_4. For Raman spectrum, the peak *D* and *G* represented the disordered carbon and graphitic carbon, respectively. The integral area ratios of I_D/I_G indicate that the PSB-CuN_3 and PS-CuN_4 samples were disordered with a large number of defects.

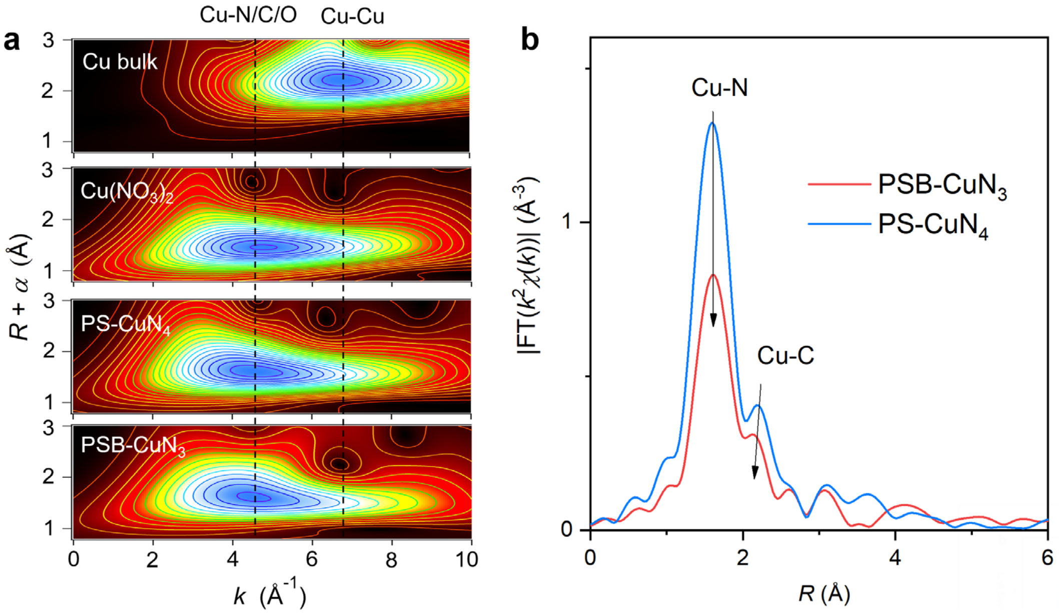

Supplementary Fig. 12. a, Wavelet transforms (WT) of Cu *K*-edge EXAFS signals based on Morlet wavelets with optimum resolutions at 2.0 Å. **b**, Fourier-transformed (FT) magnitudes of Cu *K*-edge EXAFS signals. For EXAFS-WT, the location of the intensity maximum on the *k*-axis is approximately proportional to the atomic number Z of the coordination atom. By comparison to the Cu bulk and $Cu(NO_3)_2$ references, it can be obtained that the nearly identical intensity maxima near 4.2 $Å^{-1}$ in PSB-CuN_3 and PS-CuN_4 samples indicate the adoption of Cu-N/C bonding in the first two coordination spheres, and the disappearance of intensity maxima at about 7.0 $Å^{-1}$ that corresponds to the Cu-Cu contributions suggests that Cu species are dispersed as mononuclear centres without the presence of metal-derived crystalline structures. From EXAFS-FT, it can be observed that the two peak intensities at ~ 1.60 and 2.20 Å in PSB-CuN_3 are obviously weaker than that in PS-CuN_4, suggesting a lower coordination configuration for Cu atoms in PSB-CuN_3.

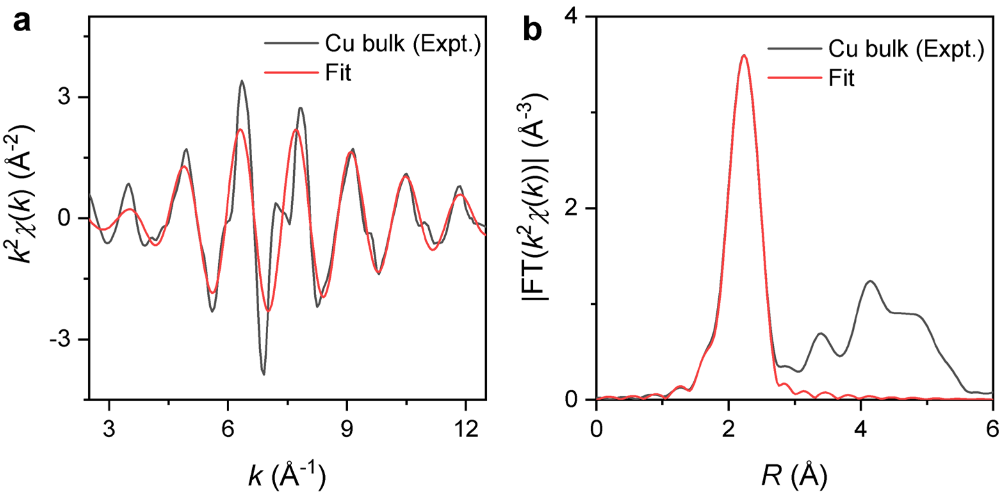

Supplementary Fig. 13. a,**b**, Cu *K*-edge EXAFS curve-fitting in *k* space (**a**) and its fourier-transformed magnitude in *R* space (**b**) for Cu bulk. The measured and calculated spectra are well matched, and the best-fit parameters are shown in Supplementary Table 1.

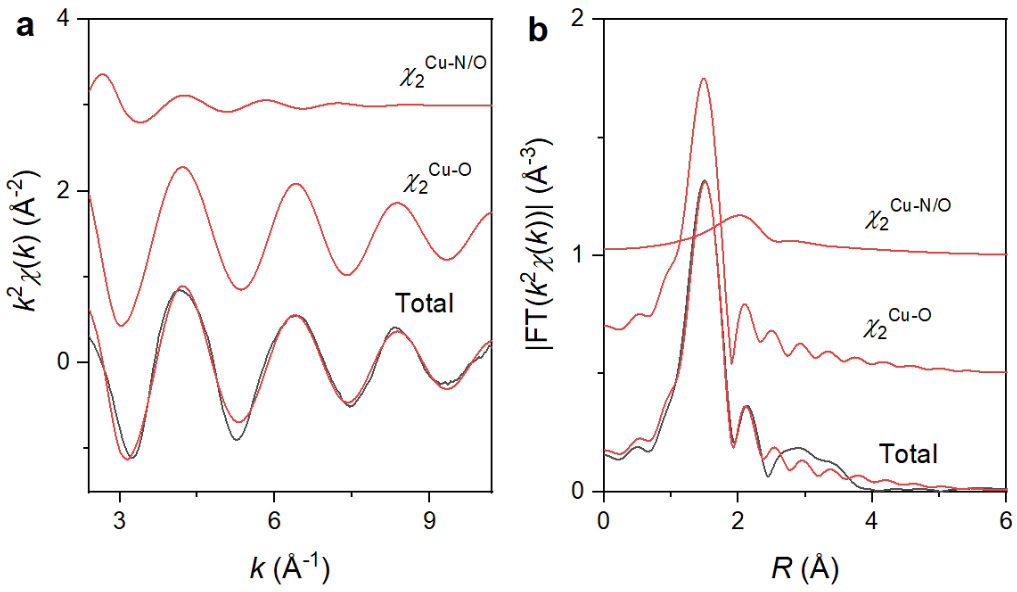

Supplementary Fig. 14. a,**b**, Cu *K*-edge EXAFS curve-fitting in *k* space (**a**) and its fourier-transformed magnitude in *R* space (**b**) for $Cu(NO_3)_2$ reference. Curves from top to bottom are the second-coordination Cu–N/O and first-coordination Cu–O two-body backscattering signals χ_2 included in the fit and the total signal (red line) superimposed on the experimental signal (black line). The measured and calculated spectra are well matched, and the best-fit parameters are shown in Supplementary Table 1.

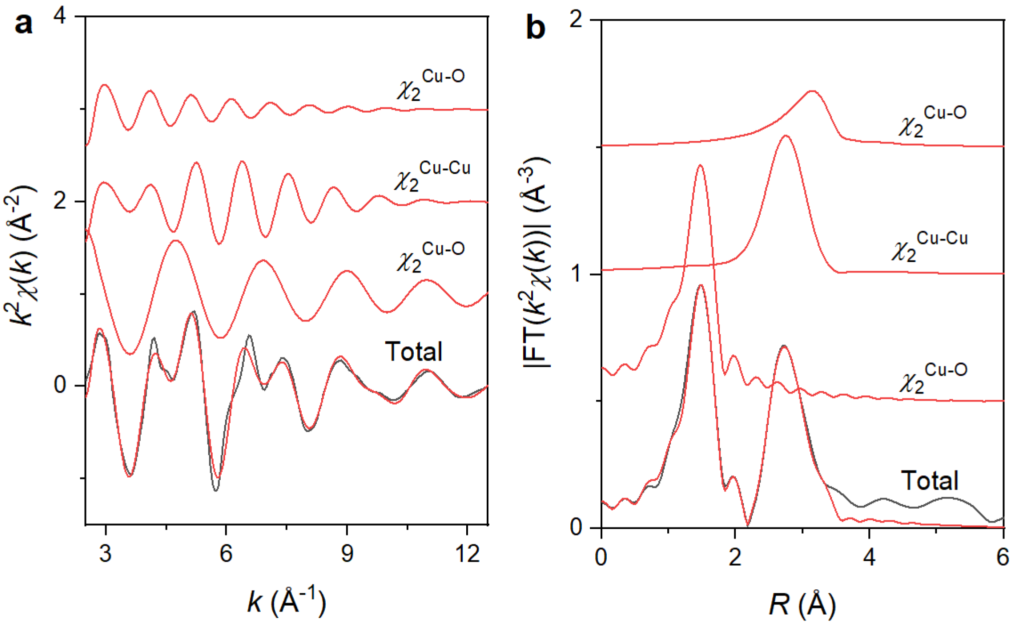

Supplementary Fig. 15. a,**b**, Cu *K*-edge EXAFS curve-fitting in *k* space (**a**) and its fourier-transformed magnitude in *R* space (**b**) for Cu_2O reference. Curves from top to bottom are the Cu–O, Cu–Cu and Cu–O two-body backscattering signals χ_2 included in the fit and the total signal (red line) superimposed on the experimental signal (black line). The measured and calculated spectra are well matched, and the best-fit parameters are shown in Supplementary Table 1.

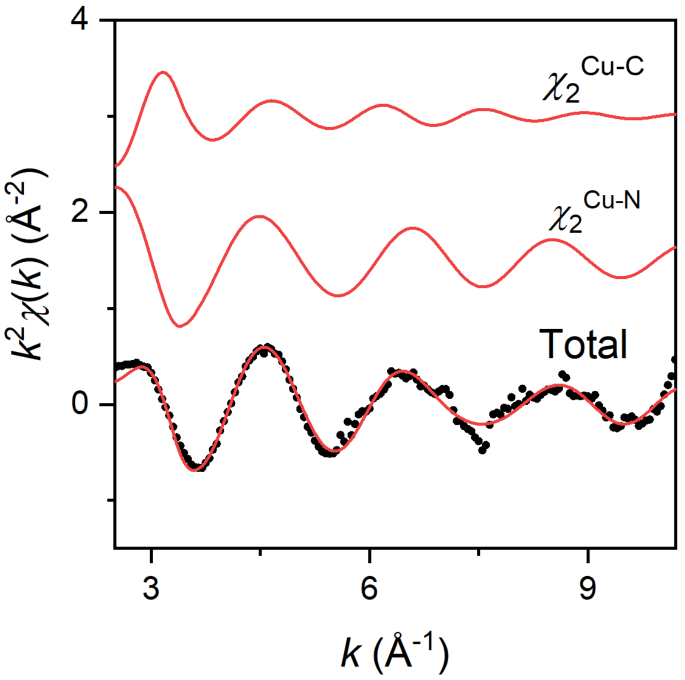

Supplementary Fig. 16. Cu *K*-edge EXAFS curve-fitting in *k* space for PSB-CuN_3 sample. Curves from top to bottom are the Cu–C and Cu–N two-body backscattering signals χ_2 included in the fit and the total signal (red line) superimposed on the experimental signal (black dots). The measured and calculated spectra are well matched for all samples. The best-fit parameters are shown in Supplementary Table 1.

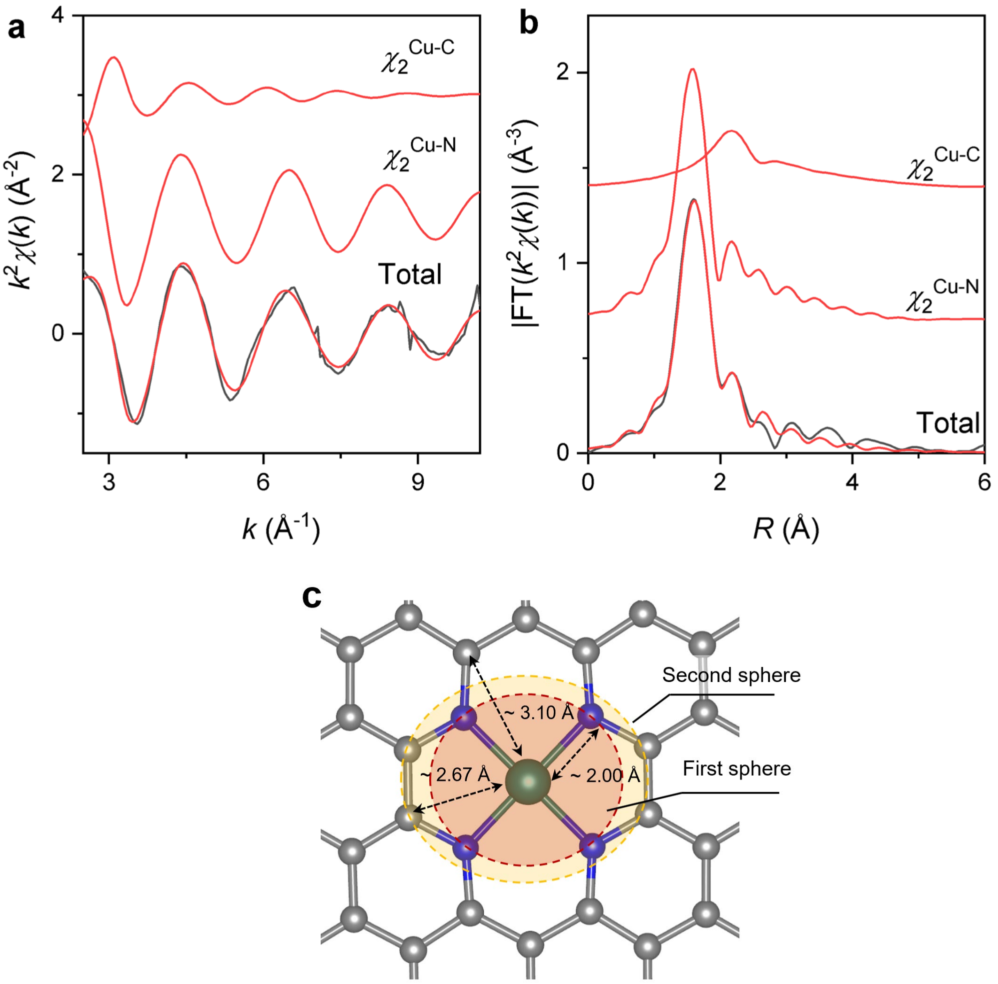

Supplementary Fig. 17. a,**b**, Cu *K*-edge EXAFS curve-fitting in *k* space (**a**) and its fourier-transformed magnitude in *R* space (**b**) for PS-CuN_4 sample. Curves from top to bottom are the Cu–C and Cu–N two-body backscattering signals χ_2 included in the fit and the total signal (red line) superimposed on the experimental signal (black line). The measured and calculated spectra are well matched for all samples. The best-fit parameters are shown in Supplementary Table 1. **c**, The assignment of the first and second coordination spheres in the CuN_4C_4 structure based upon the radial distribution of the N and C atoms around the central Cu atom. The interatomic Cu-N and Cu-C distances and their corresponding coordination spheres are indicated.

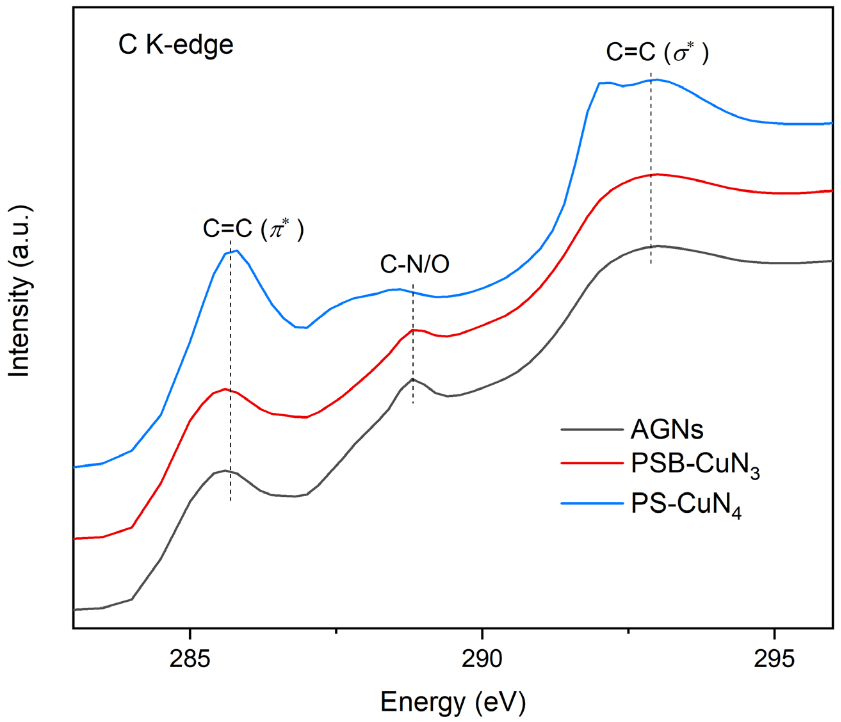

Supplementary Fig. 18. C *K*-edge soft XANES spectra of AGNs, PSB-CuN_3 and PS-CuN_4.The C *K*-edge spectra show three obvious peaks located at 285.6, 288.8 and 292.9 eV, respectively. While the two strong peaks at 285.6 and 292.9 eV correspond to corresponding to C=C (π*) and C=C (σ*) excitations, which suggest the high degree of graphitization, the noticeable peak at 288.8 eV can originate from the attached C-N/O bonds, in accordance with the C 1*s* XPS results in Supplementary Fig. 7.

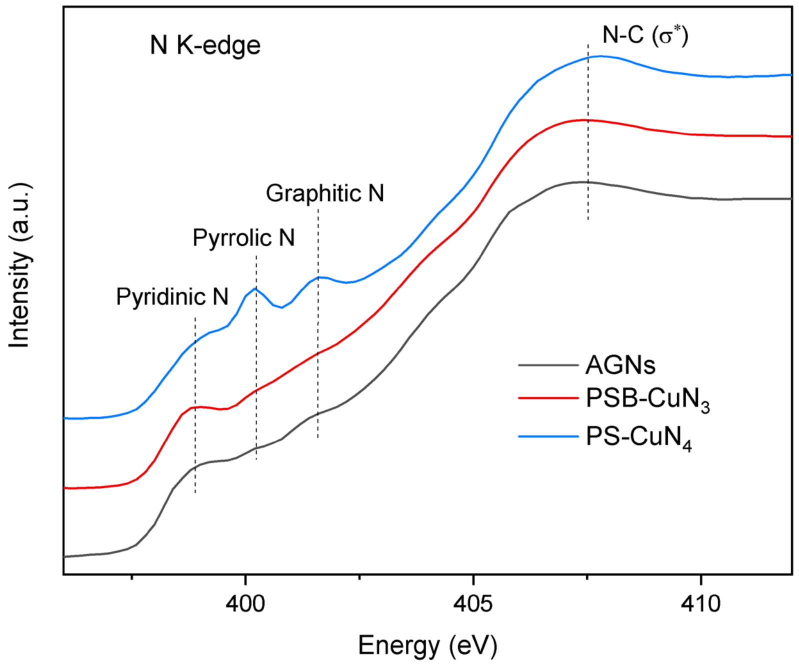

Supplementary Fig. 19. N *K*-edge soft XANES spectra of AGNs, PSB-CuN_3 and PS-CuN_4. Besides the N-C (σ*) bond at 407.5 eV, the N *K*-edge spectra show three obvious peaks located at 398.8, 400.2 and 401.6 eV, which suggests the presence of pyridinic N (π*), pyrrolic N (π*) and graphitic N (π*) bonds, in good agreement with the N 1*s* XPS results in Supplementary Fig. 8.

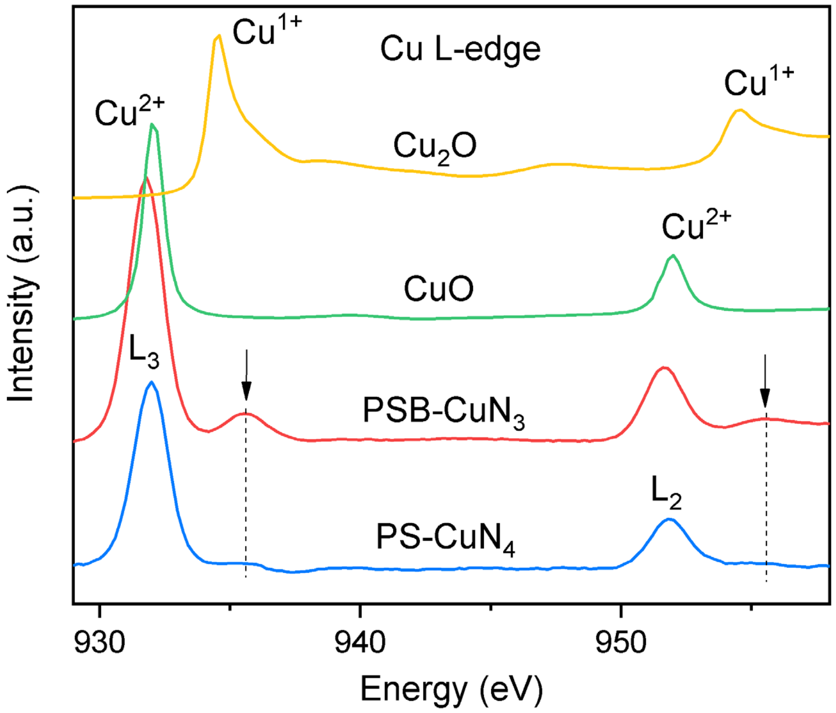

Supplementary Fig. 20. Comparison of soft Cu $L_{3,2}$-edge XANES spectra for PSB-CuN_3, PS-CuN_4, CuO and Cu_2O references. When shifted from PS-CuN_4 to PSB-CuN_3, besides the two main absorption peaks at ~931.7 eV (L_3) and 951.6 eV (L_2), the Cu $L_{3,2}$-edge spectra reveal an emergence of two extra post-edge resonant peaks at 935.6 and 955.4 eV that coincide with the positions of Cu_2O. It indicates the existence of Cu^{1+} species in PSB-CuN_3, thus consolidating the Cu hard *K*-edge XANES results in Fig. 2d and the Cu 2*p* and LMM XPS results in Supplementary Figs. 9 and 10.

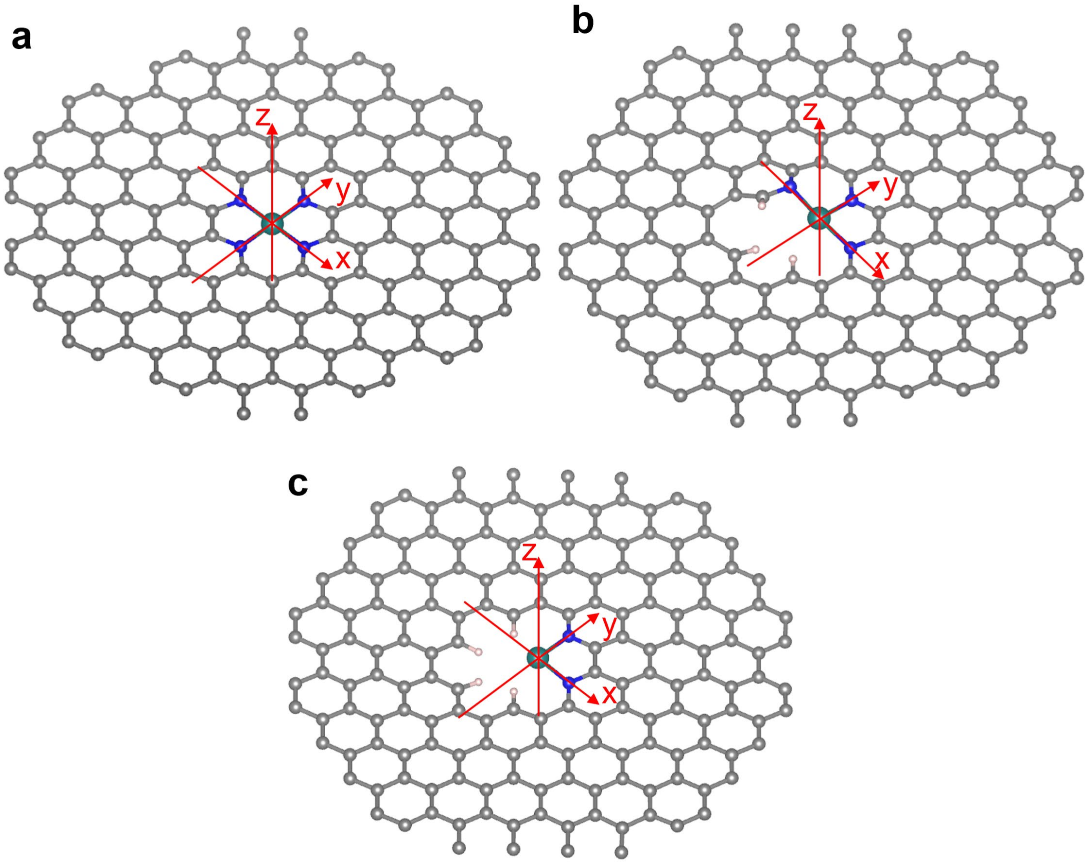

Supplementary Fig. 21. Different CuN_xC_y models used for XANES calculation. **a**, CuN_4C_4. **b**, Defective CuN_3C_3 with one N vacancy along the *y* axis. **c**, Defective CuN_2C_2 with two N vacancies along the *x* and *y* axes, respectively.

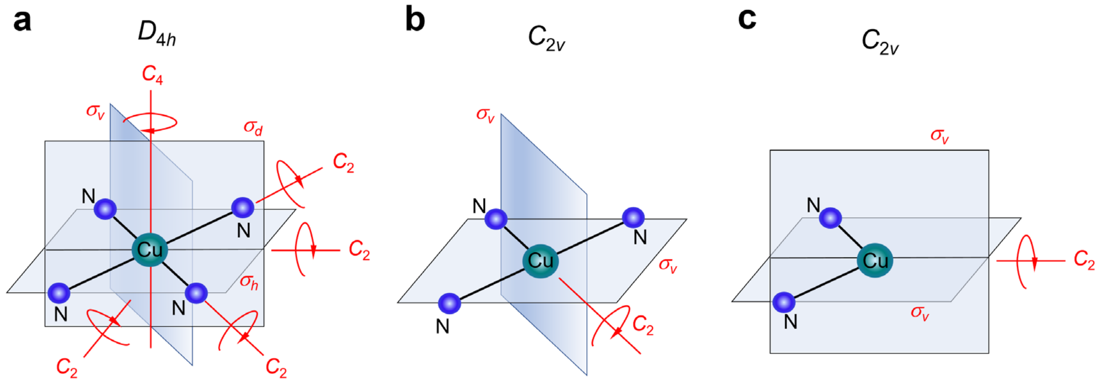

Supplementary Fig. 22. The local symmetry elements and operations for different CuN_x structures in the first coordination spheres. **a**, CuN_4. **b**, CuN_3. **c**, CuN_2. It can be observed that while the CuN_4 structure shows a planar D_{4h} symmetry that includes a C_4 symmetry axis perpendicular to the CuN_4 plane and four C_2 symmetry axes in the CuN_4 plane, the introduction of N vacancy in CuN_3 and CuN_2 structures leads to a breaking of the C_4 symmetry operation perpendicular to the plane and three C_2 symmetry operations in the plane, which corresponds to a lower C_{2v} symmetry in the CuN_3 and CuN_2 structures.

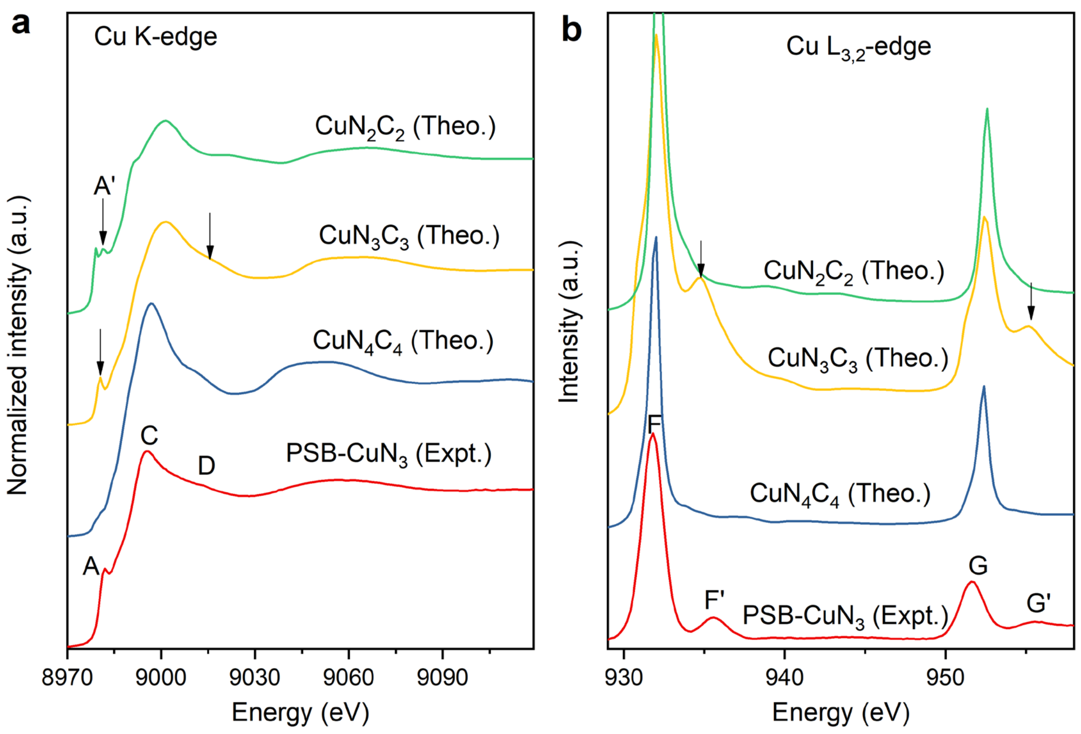

Supplementary Fig. 23. a,**b**, Comparison between the experimental Cu *K*-edge (i.e., 1*s* → 4*p* transition; **a**) and $L_{3,2}$-edge (i.e., 2*p* → 3*d* transition; **b**) XANES spectrum of PSB-CuN_3 and the theoretical spectra calculated with the CuN_4C_4, CuN_3C_3 and CuN_2C_2 structures. It can be observed that, only when a N defect is introduced in the defective CuN_3C_3 structure, the characteristic near-edge peak A and the flattened post-edge peak D in *K*-edge as well as the post-edge peaks F' and G' in $L_{3,2}$-edge can be precisely reproduced. By contrast, the CuN_2C_2 structure shows two minor near-edge peaks at ~ 8979 eV and two prominent peaks at 8991 and 9020 eV in *K*-edge; in $L_{3,2}$-edge, it also doesn't show post-edge peaks at the energy positions of F' and G'. Therefore, those dramatic discrepancies in both *K* and $L_{3,2}$-edge profiles exclude the probability for the CuN_2C_2 structure.

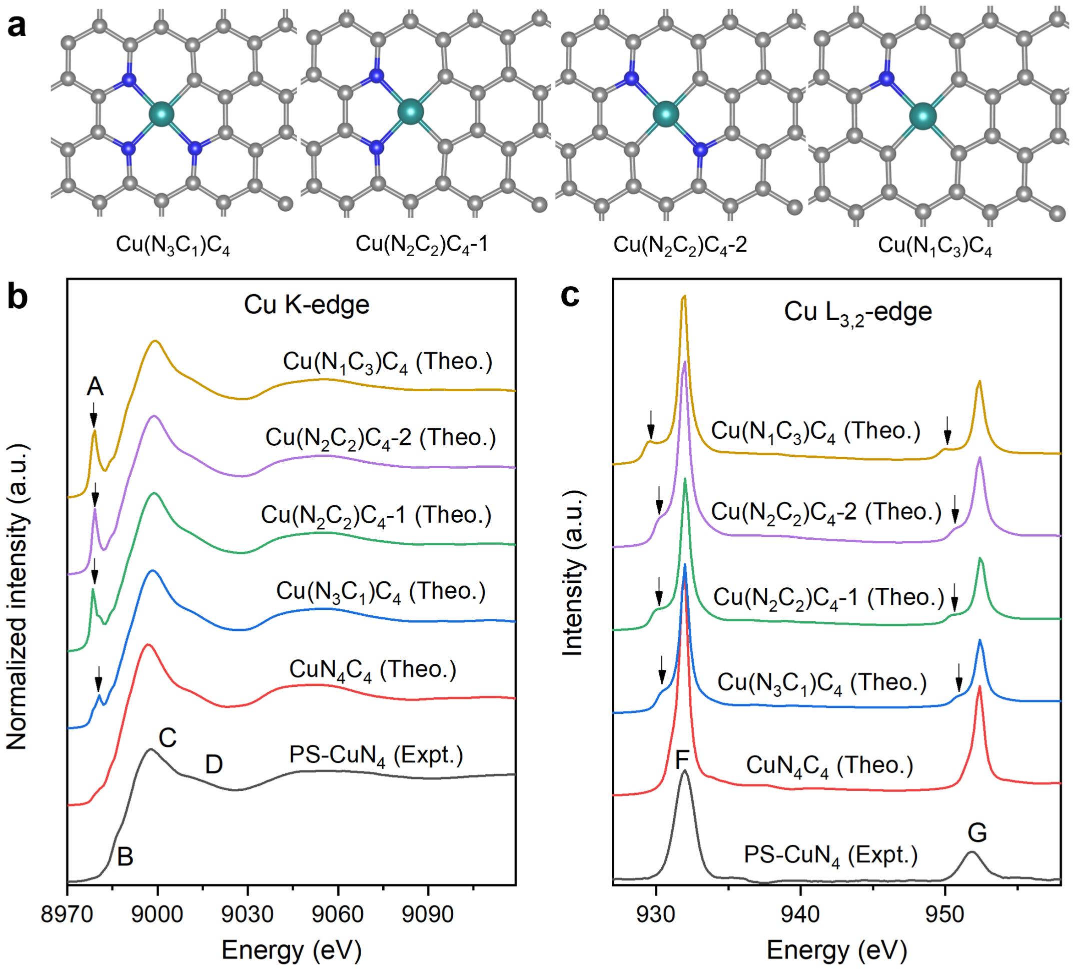

Supplementary Fig. 24. **a**, Various $Cu(N_{4-x}C_x)C_4$ structures. The $Cu(N_3C_1)C_4$, $Cu(N_2C_2)C_4$ and $Cu(N_1C_3)C_4$ moieties are derived from the CuN_4C_4 ($x = 0$) model with different amount of N atoms in the first coordination shell substituted with C atoms. **b**,**c**, Comparison between the experimental Cu K-edge (**b**) and $L_{3,2}$-edge (**c**) XANES spectra of PS-CuN_4 and the theoretical spectra calculated for the depicted structures. The arrows highlight the emergence of pre-edge peaks due to C substitution by N atoms.

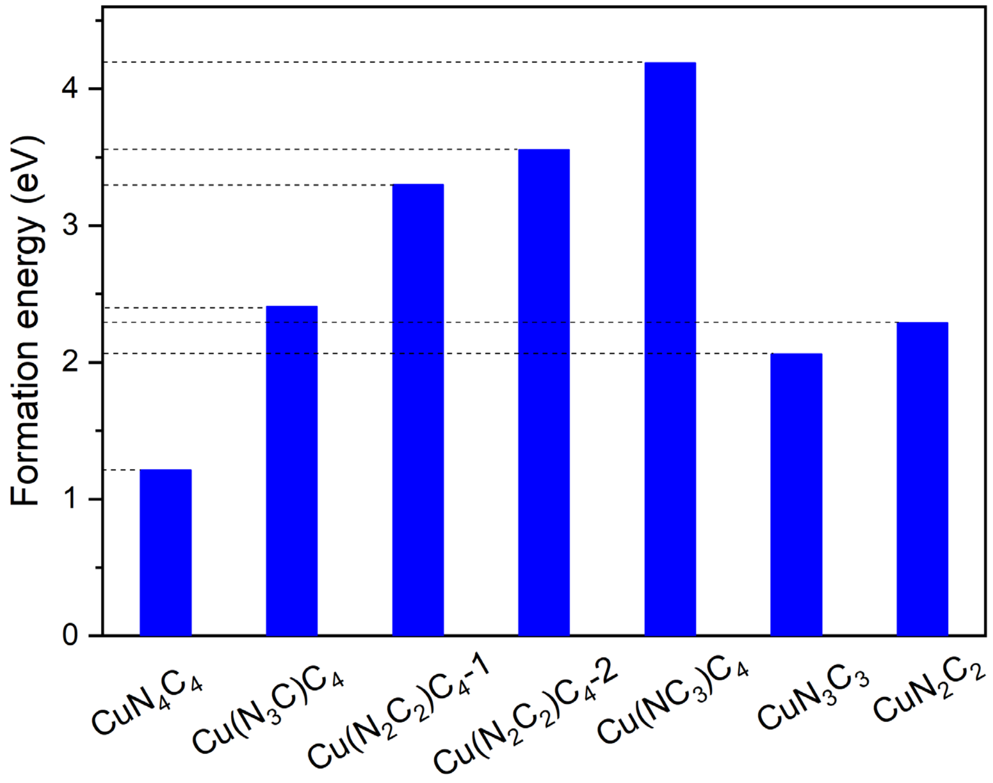

Supplementary Fig. 25. The formation energy of various CuN_xC_y model structures.

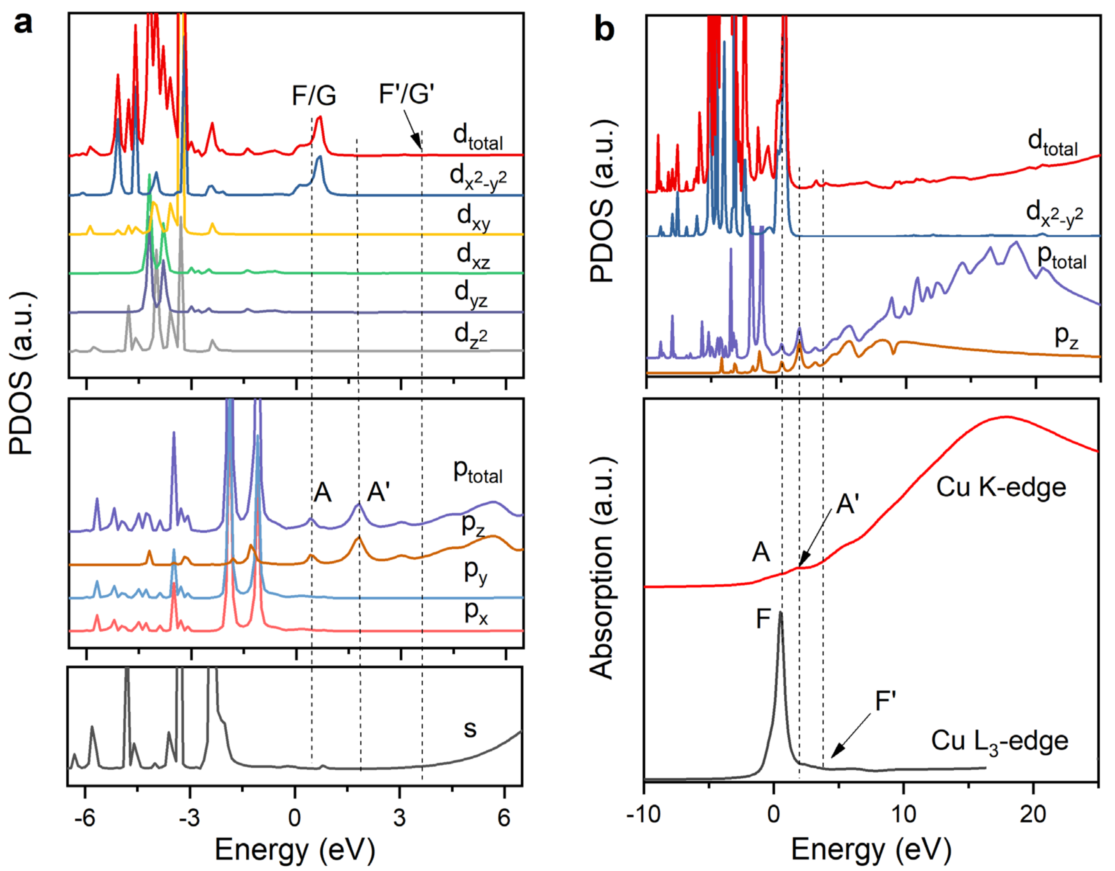

Supplementary Fig. 26. a, Detailed comparison of the total and partial *s*, *p*, *d* density of states on the Cu site of the planar CuN_4C_4 structure with local D_{4h} symmetry. **b**, Comparison between the total and dominant partial density of states along with simulated XANES spectra at Cu *K* and L_3-edge for the CuN_4C_4 structure. The dashed lines corresponding to the energy positions for the feature peaks (A, A', F, F', G and G') in the Cu *K* and L_3-edge XANES are provided to guide the eyes. The related discussion and explanation please see Supplementary Note 2.

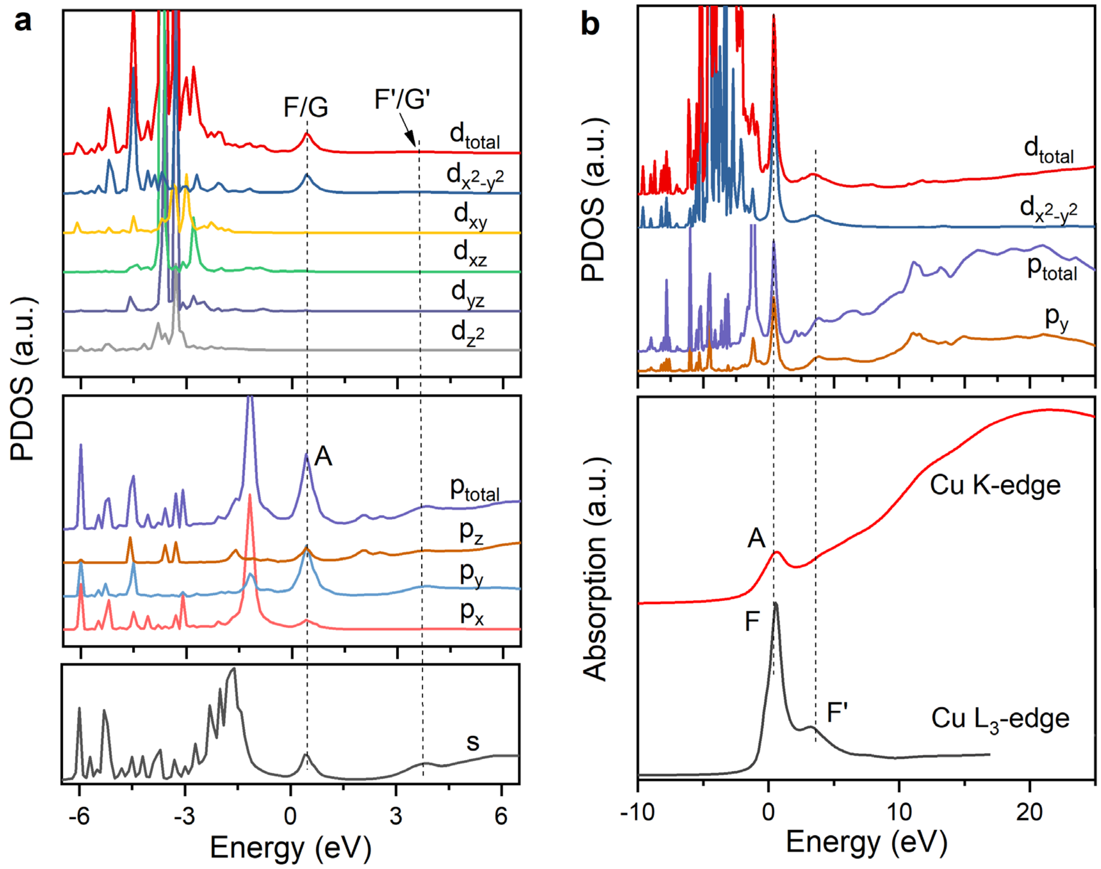

Supplementary Fig. 27. **a**, Detailed comparison of the total and partial *s*, *p*, *d* density of states on the Cu site of the defective CuN_3C_3 structure with broken D_{4h} symmetry (i.e., C_{2v}). **b**, A comparison between the total and partial density of states along with simulated XANES spectra at Cu *K* and L_3-edge for the CuN_3C_3 structure. The dashed lines corresponding to the energy positions for the feature peaks (A, F, F', G and G') in the Cu *K* and L_3-edge XANES are provided to guide the eyes. The related discussion and explanation please see Supplementary Note 2.

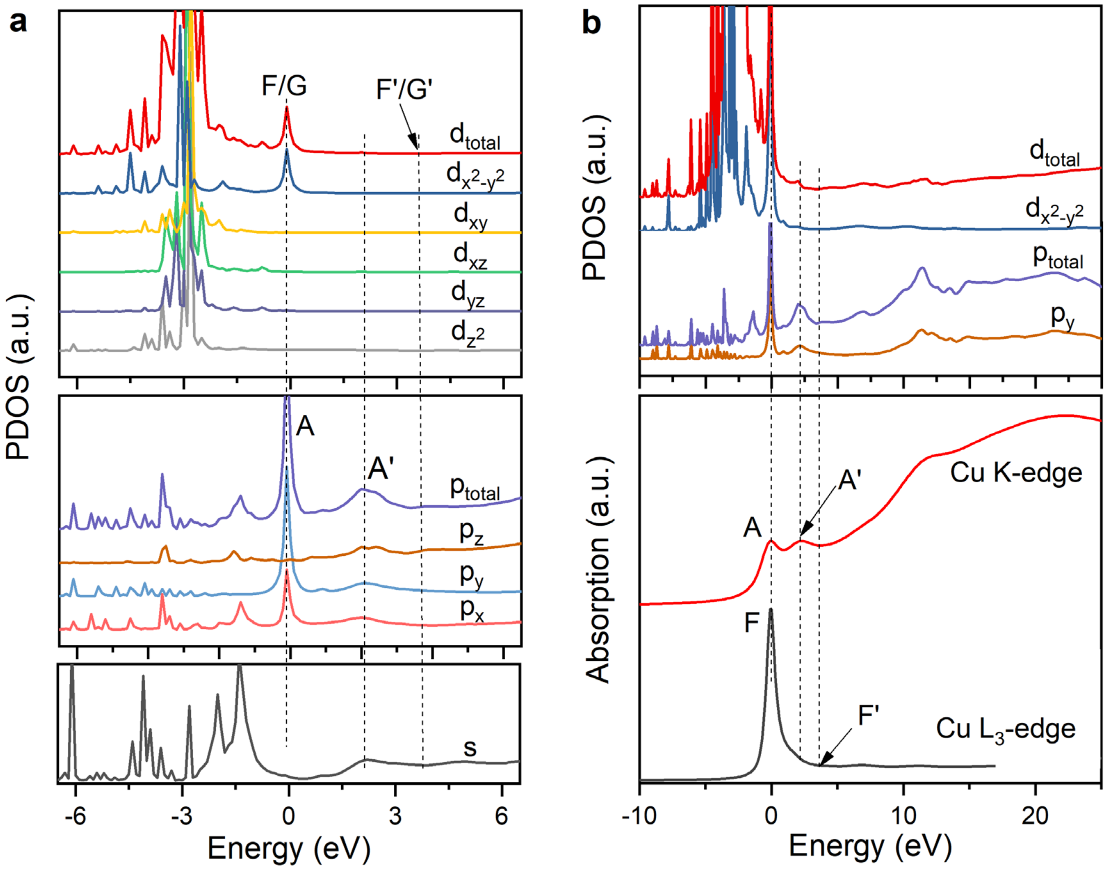

Supplementary Fig. 28. a, Detailed comparison of the total and partial *s*, *p*, *d* density of states on the Cu site of the defective CuN_2C_2 structure with broken D_{4h} symmetry (i.e., C_{2v}). **b**, A comparison between the total and partial density of states along with simulated XANES spectra at Cu *K* and L_3-edge for the CuN_2C_2 structure. The dashed lines corresponding to the energy positions for the feature peaks (A, A', F, F', G and G') in the Cu *K* and L_3-edge XANES are provided to guide the eyes. The related discussion and explanation please see Supplementary Note 2.

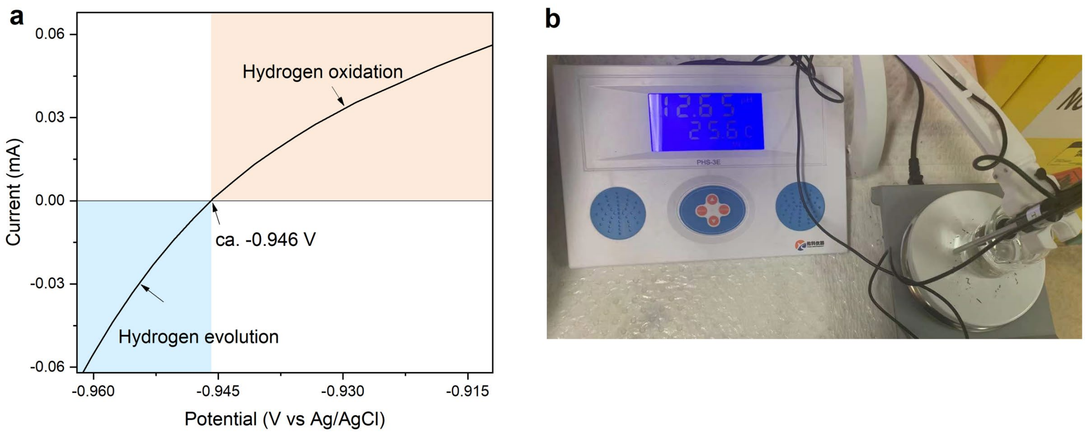

Supplementary Fig. 29. Calibration of the used reference electrode and pH measurement of the electrolyte. **a,** Calibration of the used Ag/AgCl electrode performed using a conventional three-electrode cell with 0.1M KOH solution. **b,** pH measurement of the 0.1M KOH electrolyte under a constant stirring rate of 100 rpm. As shown in Supplementary Fig. 29, the zero current point is at about -0.946 V in 0.1 M KOH and the pH value of the used electrolyte (0.1 M KOH) is ~ 12.65. According to E(RHE) = E (Ag/AgCl) + 0.946 V, therefore, the potential of the used Ag/AgCl electrode is ~0.198 V, which is very close to the standard potential (0.197 V, Ag/AgCl/Sat. KCl).

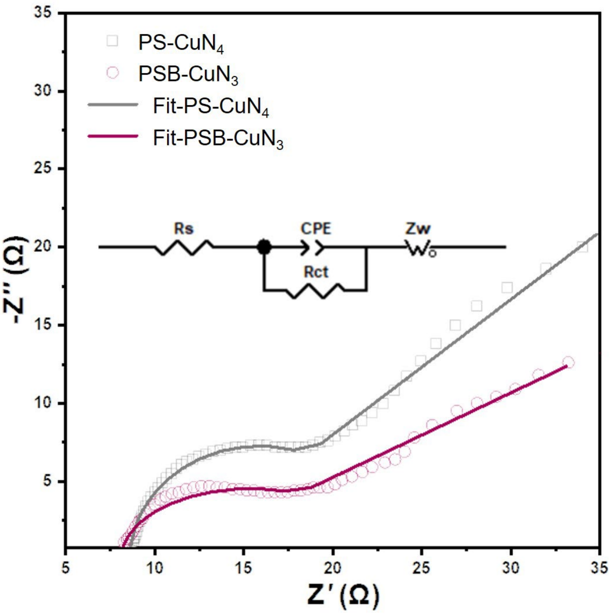

Supplementary Fig. 30. EIS spectra of PSB-CuN_3 and PS-CuN_4 catalysts at OCP. Compared to PS-CuN_4 catalyst (9.0 Ω), the solution impedance of PSB-CuN_3 showed a lower value of 8.3 Ω.

EIS measurements were conducted at 25°C using an H-type cell (consisting of two independent anode and cathode chambers separated by a nafion membrane) and a CHI 760e electrochemical workstation. Platinum wire served as the counter electrode, while Ag/AgCl (saturated with KCl) was employed as the reference electrode. The EIS measurement was carried out under open circuit voltage, and the frequency setting range was from low frequency 1 Hz to high frequency 100000 Hz. The amplitude was set as 0.005 V. The EIS spectra and fitting curves of PSB-CuN_3 and PS-CuN_4 catalysts are shown in Supplementary Fig. 30, which can be seen that the EIS experimental data is in good agreement with the fitting curves. The quantitative parameters including equivalent circuit and the error between the raw and fitted data are shown in Supplementary Table 9.

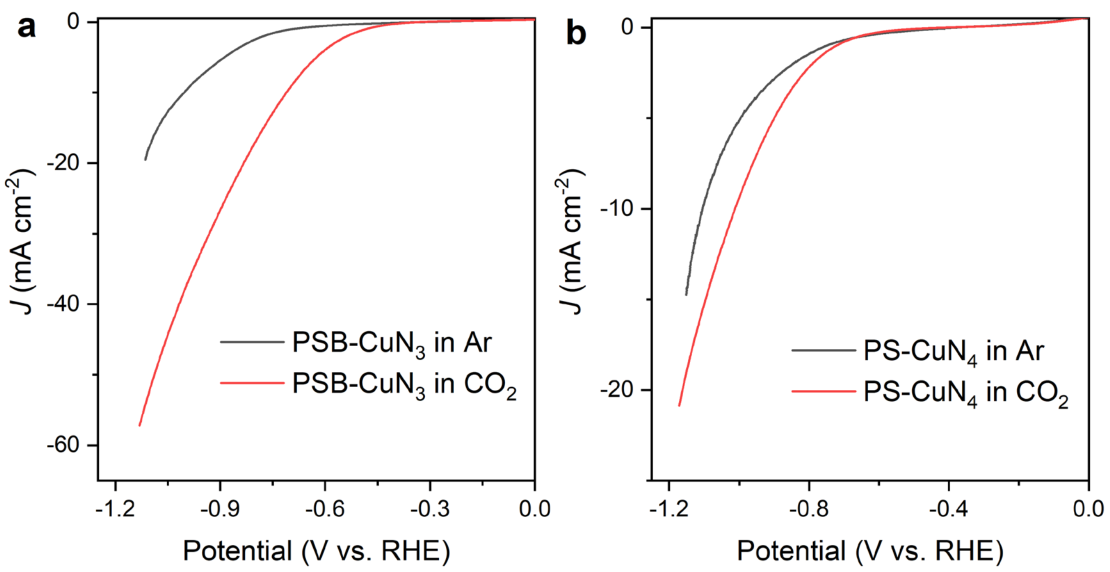

Supplementary Fig. 31. a,**b**, Comparison of LSV curves of PSB-CuN_3 (**a**) and PS-CuN_4 (**b**) in Ar- and CO_2-saturated 0.5 M $KHCO_3$ at the scan rate of 10 mV s^{-1}. The potential was iR-corrected.

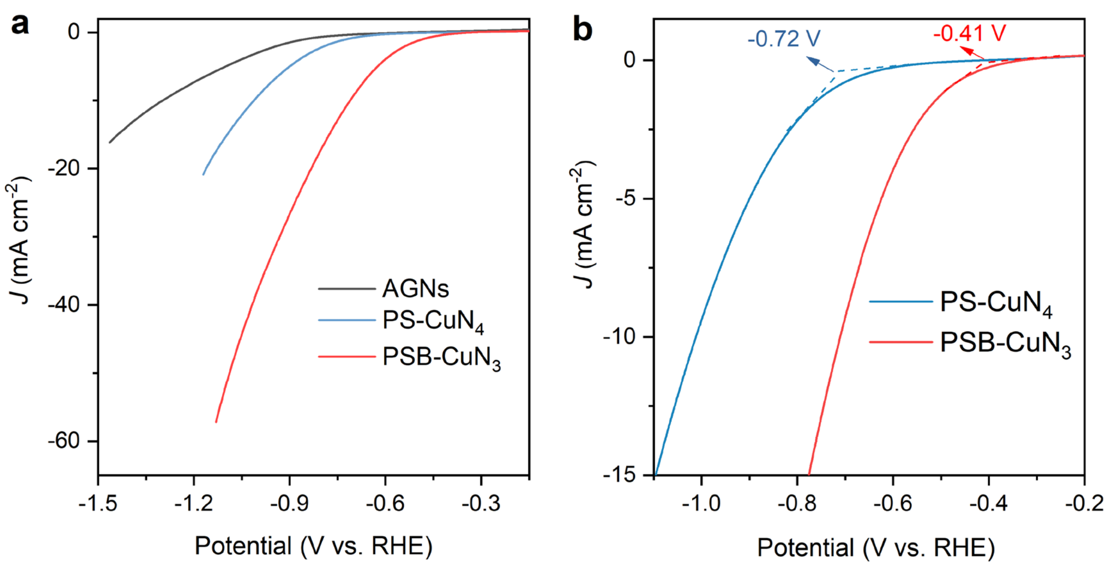

Supplementary Fig. 32. a, LSV curves of PSB-CuN3 and PS-CuN4 and AGNs performed in CO_2-saturated 0.5 M $KHCO_3$ at the scan rate of 10 mV s^{-1}. **b,** The comparison of onset potentials for PSB-CuN3 and PS-CuN4. The potential was iR-corrected.

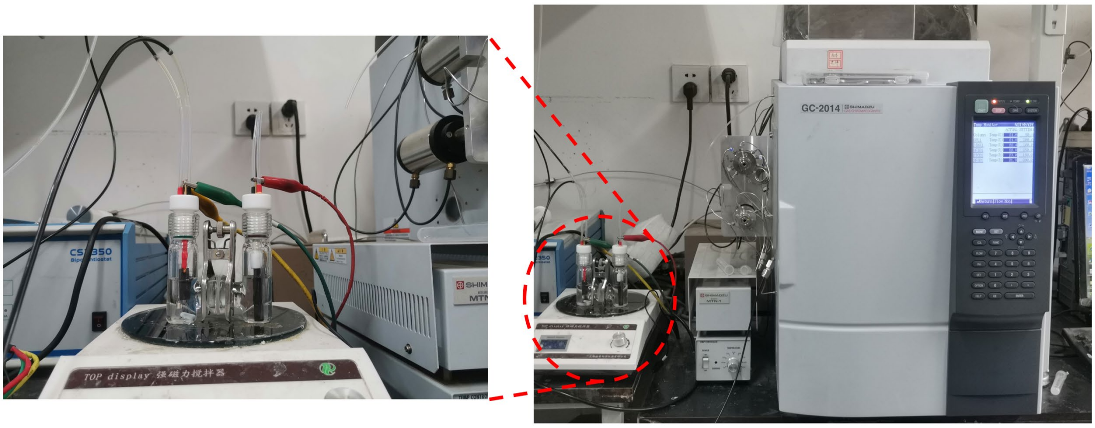

Supplementary Fig. 33. Photograph of the typical three-electrode H-type setup for the electrochemical CO_2RR measurements.

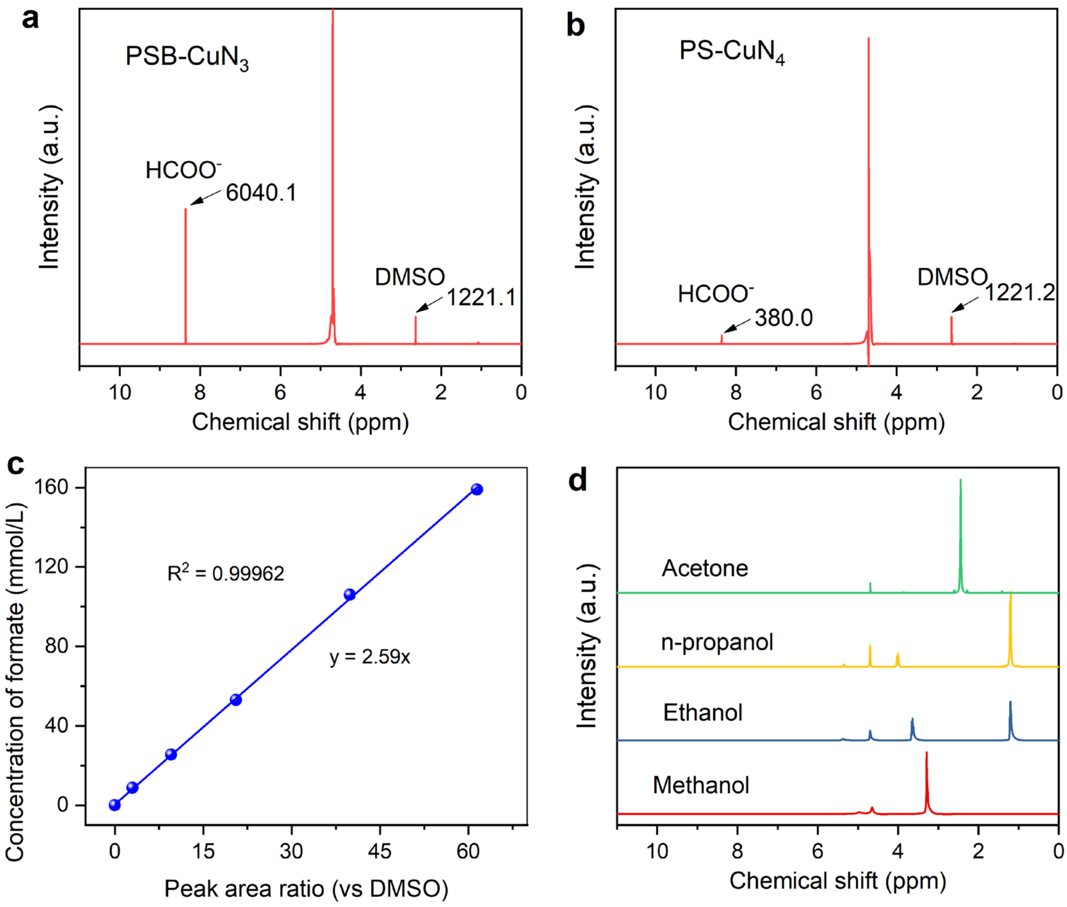

Supplementary Fig. 34. a,**b**, Representative NMR spectrum of the electrolyte after CO_2 reduction electrolysis at -0.80 V vs. RHE for PSB-CuN_3 and PS-CuN_4. DMSO is used as an internal standard for quantification of formate. **c**, Method for quantification of formate concentration. The concentration of formate is quantified through the standard curve of formate by plotting the formate concentration with respect to the formate/DMSO NMR peak area ratio. **d**, Representative NMR spectra of C_1 and C_2 liquids (i.e., methanol, ethanol, n-propanol and acetone). It should be noted that the formate FE is related to both the quantitative NMR peak intensity and the current density of LSV. By quantitative calculation, when the formate concentration of PSB-CuN_3 is 15.9 times that of PS-CuN_4, the formate FE achieves ~ 94% and 53% at -0.8 V vs. RHE on PSB-CuN_3 and PS-CuN_4 (see Supplementary Fig. 35), respectively.

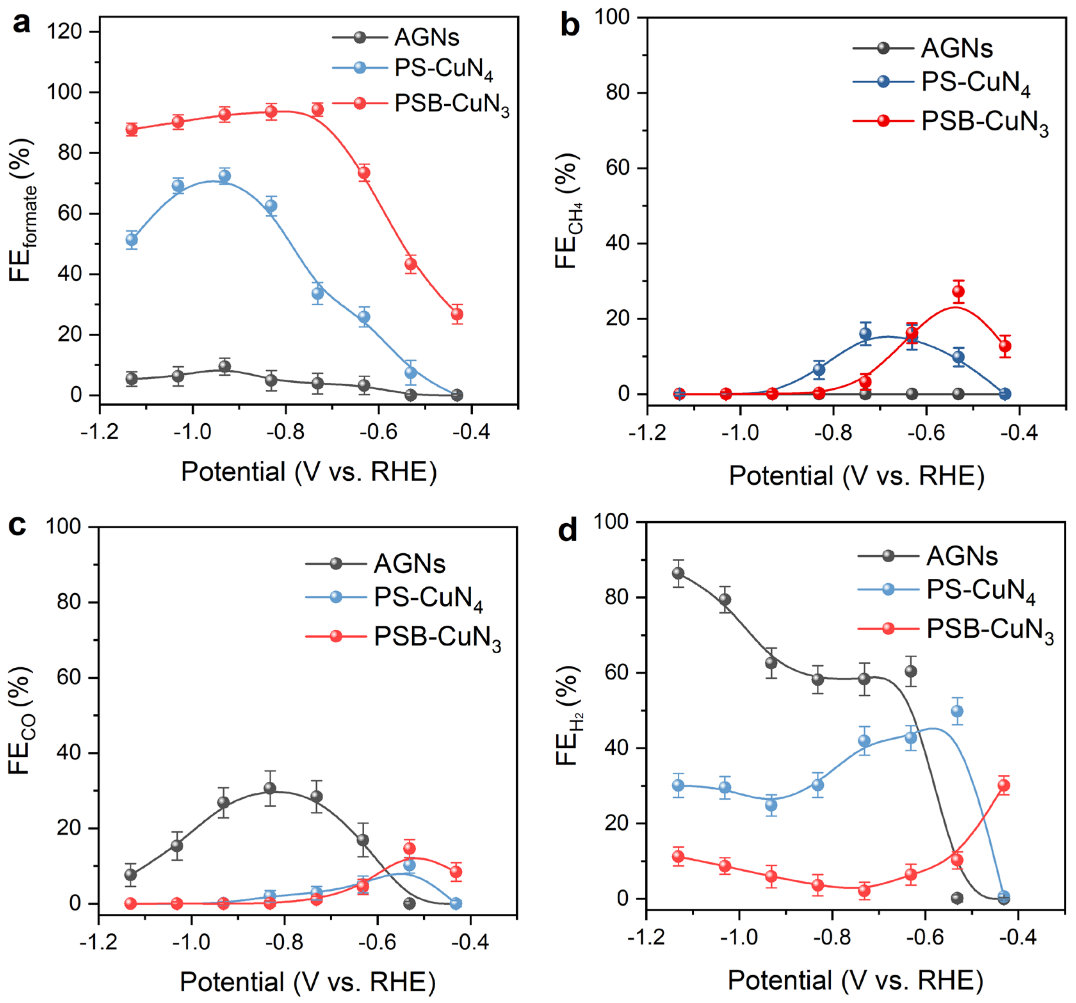

Supplementary Fig. 35. a-d, Potential dependence of Faradaic efficiencies for CO_2RR on PSB-CuN_3 and PS-CuN_4 and AGNs for formate (**a**), methane (**b**), carbon monoxide (**c**) and hydrogen (**d**) productions. The potential was iR-corrected. The presented values represent the mean, while the error bars indicate the standard deviation, based on three independent measurements.

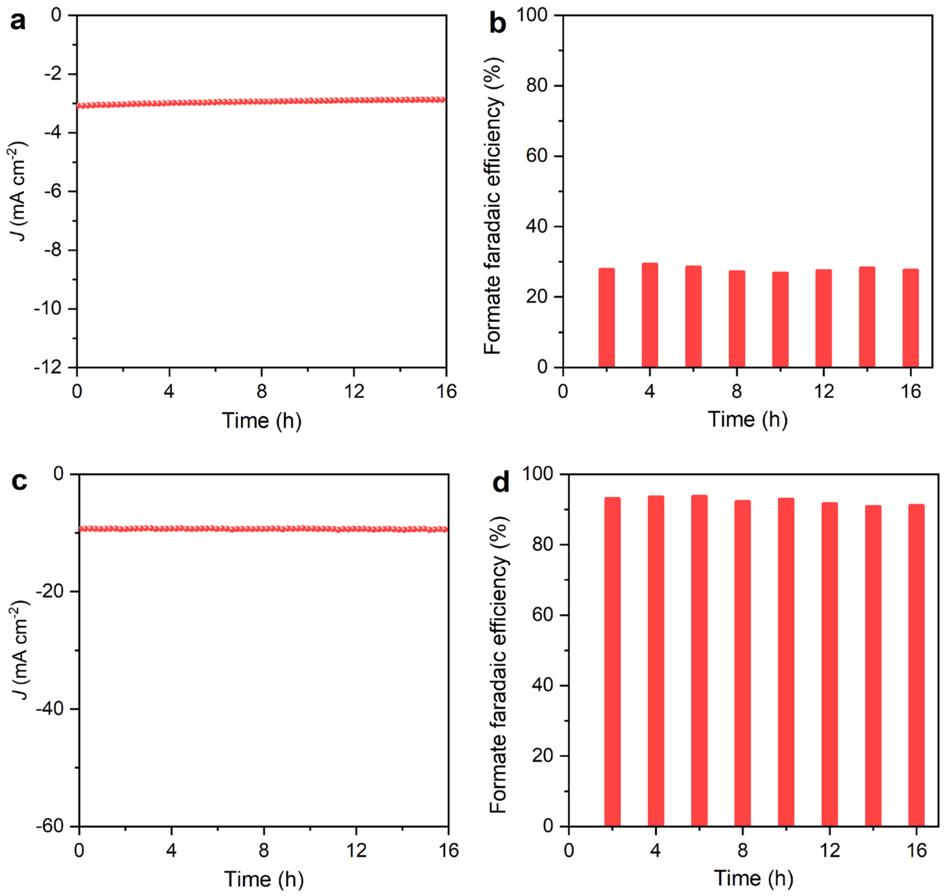

Supplementary Fig. 36. a-d, Stability test of PS-CuN_4 and PSB-CuN_3 at a potential of -0.7 V vs. RHE. (**a**) Current density and (**b**) Formate Faradaic efficiency for PS-CuN_4. (**c**) Current density and (**d**) Formate Faradaic efficiency for PSB-CuN_3. The potential was iR-corrected.

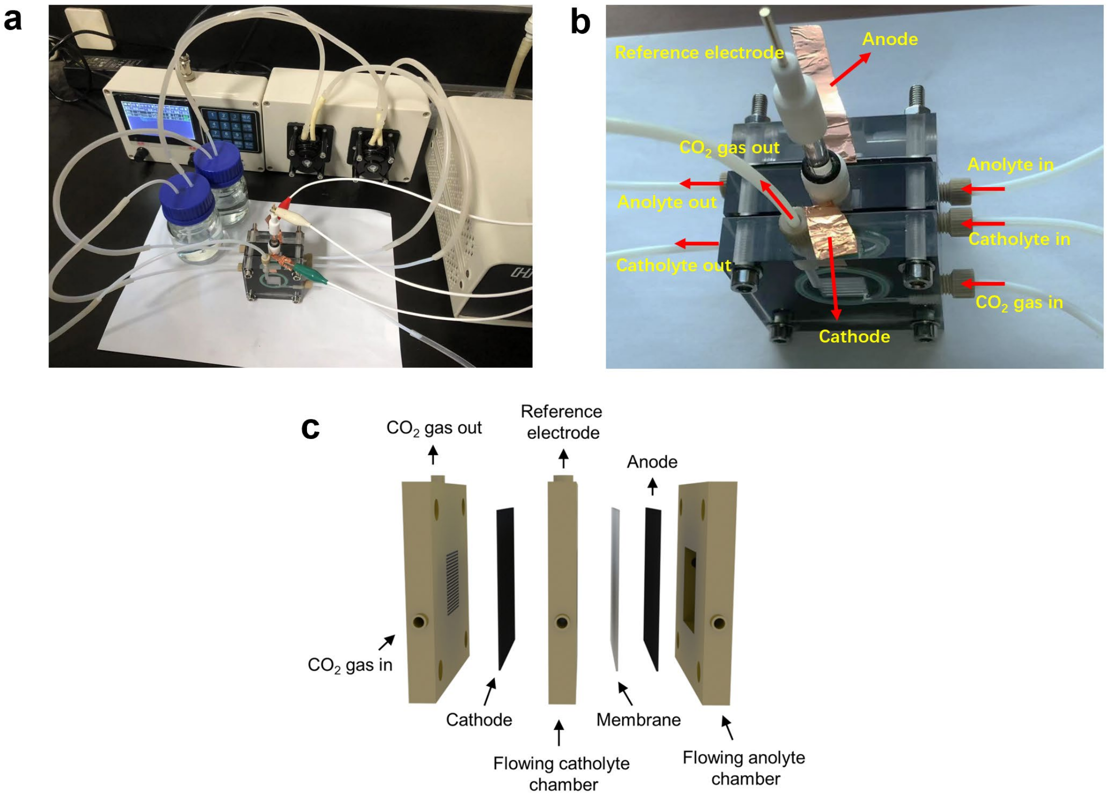

Supplementary Fig. 37. a-c, Schematic setup of electrocatalytic home-made flow reactor: Experimental test device diagram (**a**), partial enlargement experimental test device diagram (**b**), and structure diagram of flow cell (**c**).

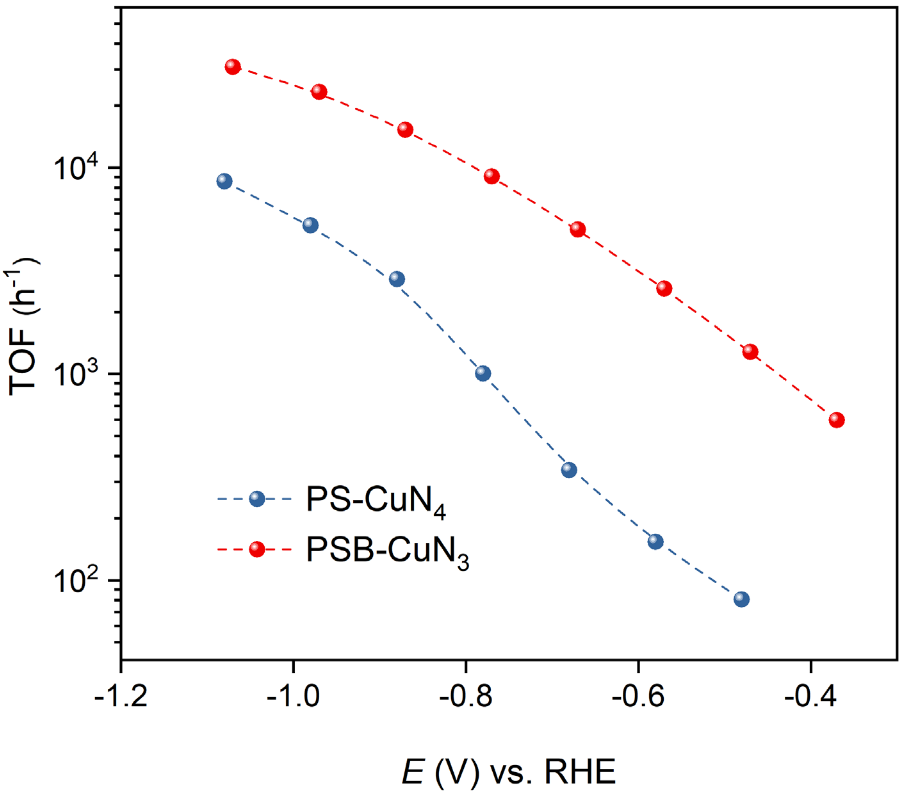

Supplementary Fig. 38. TOF of $HCOO^-$ at different potentials over PSB-CuN_3 and PS-CuN_4 catalysts. The potential was iR-corrected.

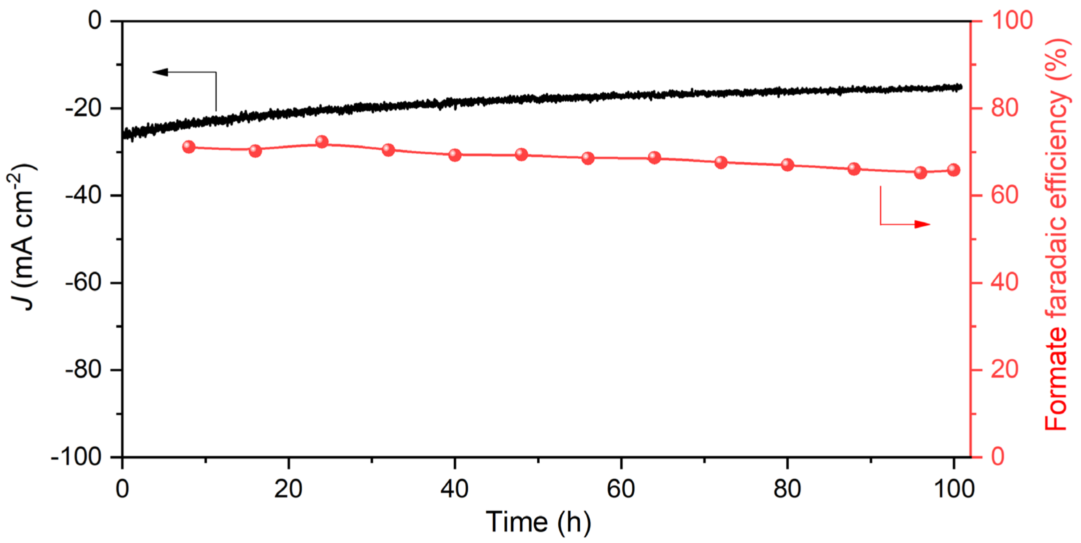

Supplementary Fig. 39. Long-term stability test of PS-CuN_4 at a potential of -0.95 V vs. RHE. The potential was iR-corrected.

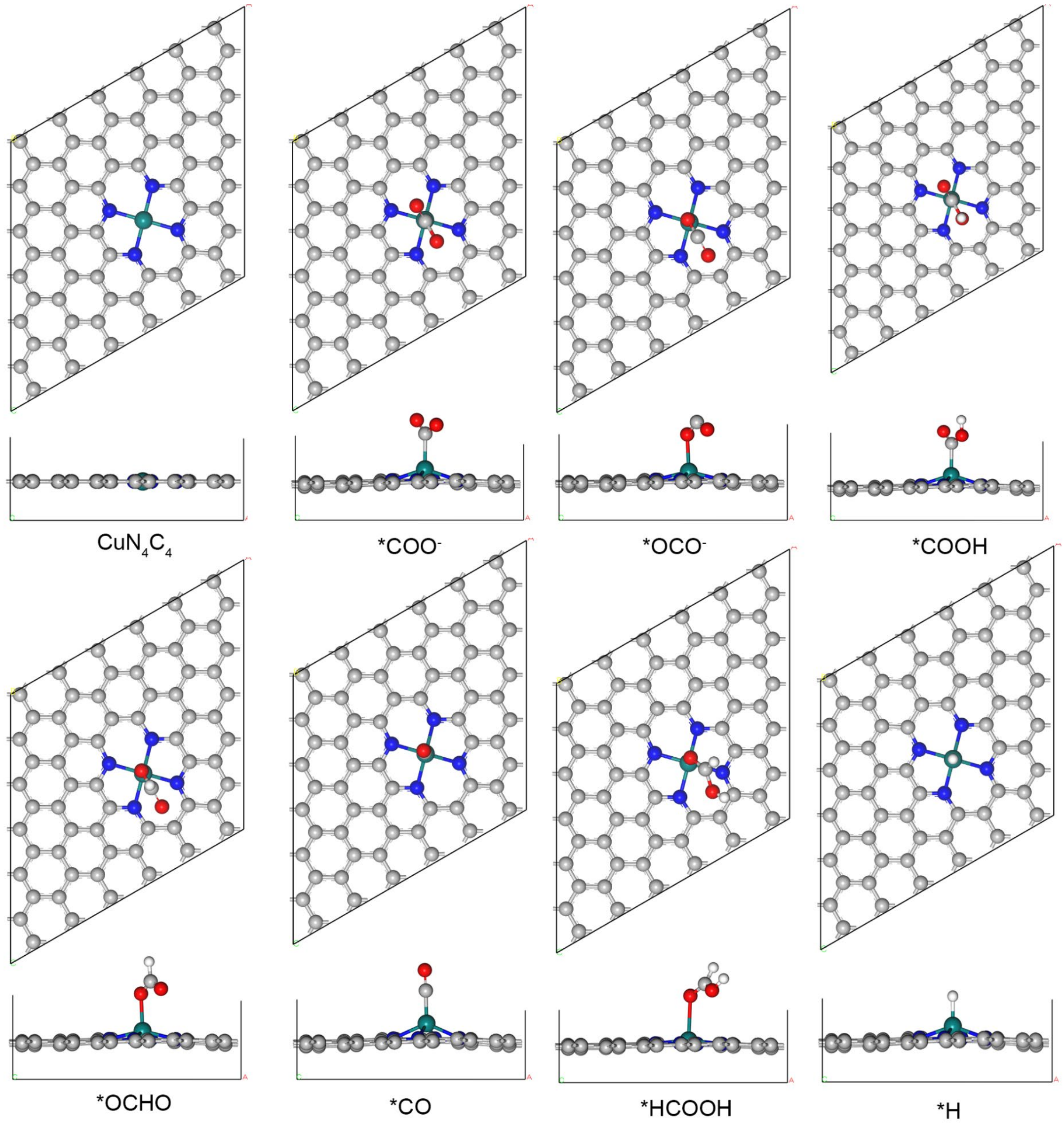

Supplementary Fig. 40. Optimized structures of CuN_4C_4 with adsorbed intermediates of *COO-, *OCO-, *COOH, *OCHO, *CO, *HCOOH and *H for CO, HCOOH and H_2 productions (dark cyan for Cu, blue for N, gray for C, red for O and white for H).

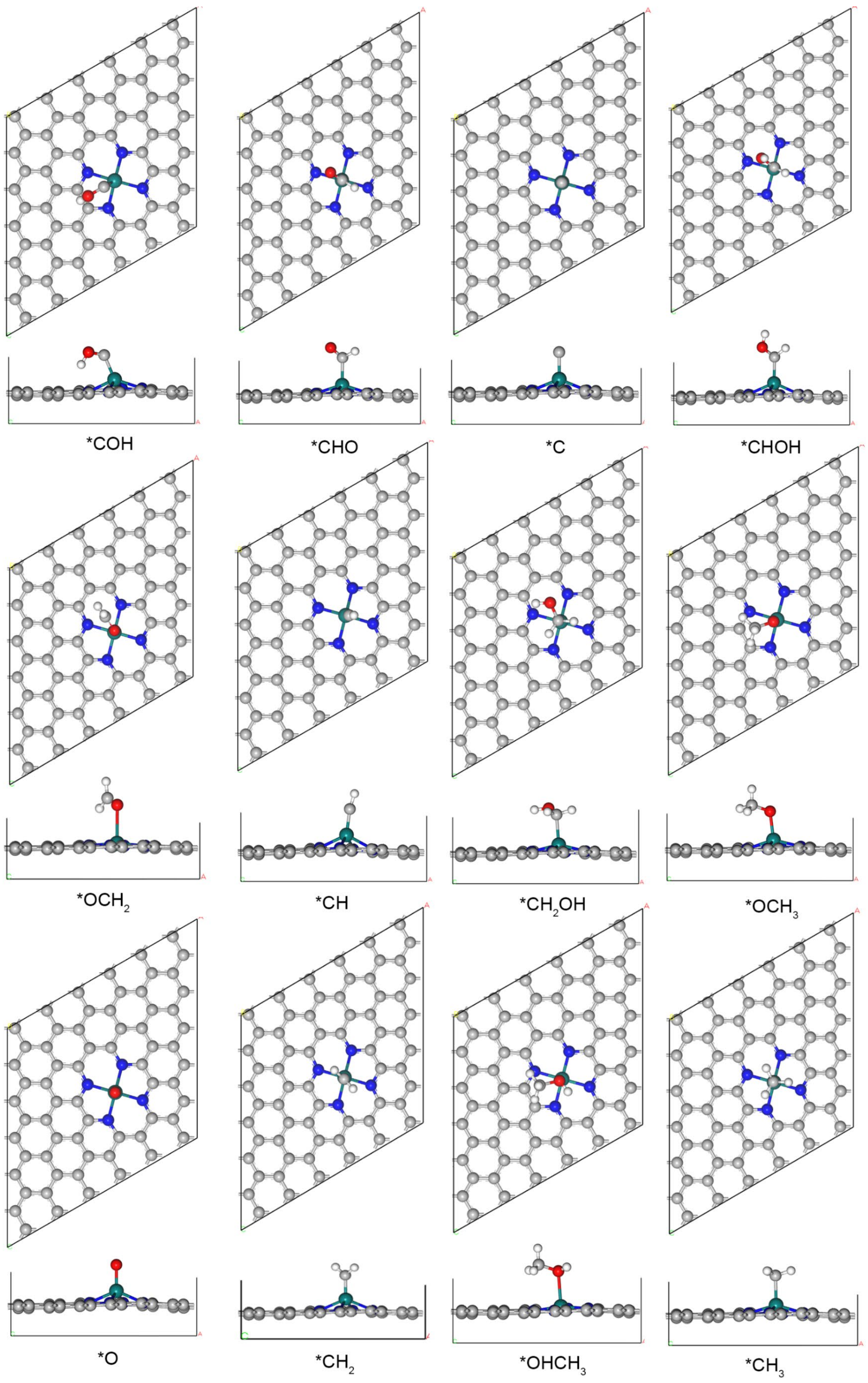

Supplementary Fig. 41. Optimized structures of CuN_4C_4 with adsorbed intermediates for CH_4 and CH_3OH productions (dark cyan for Cu, blue for N, gray for C, red for O and white for H).

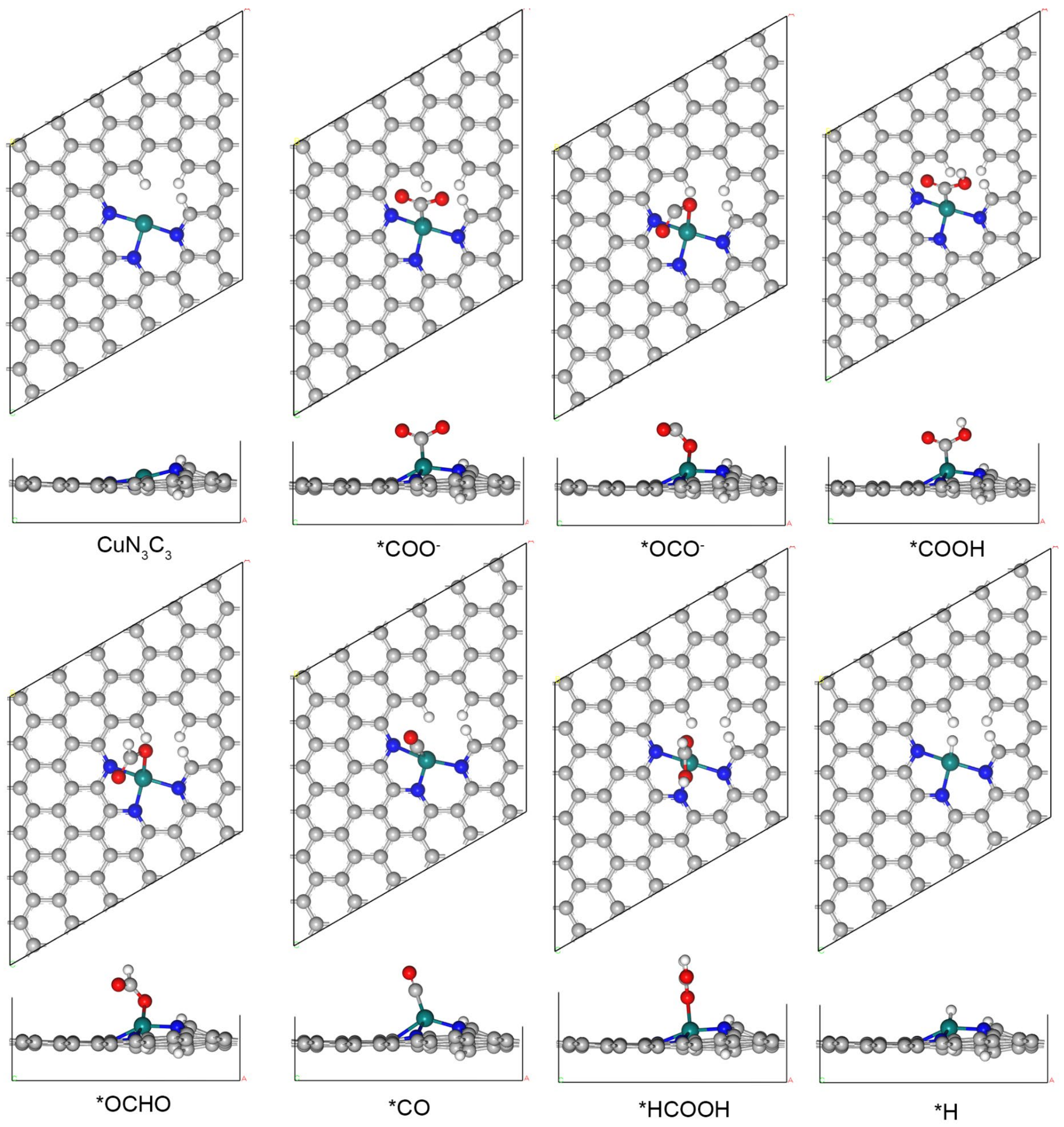

Supplementary Fig. 42. Optimized structures of CuN_3C_3 with adsorbed intermediates of $*COO^-$, $*OCO^-$, *COOH, *OCHO, *CO, *HCOOH and *H for CO, HCOOH and H_2 productions (dark cyan for Cu, blue for N, gray for C, red for O and white for H).

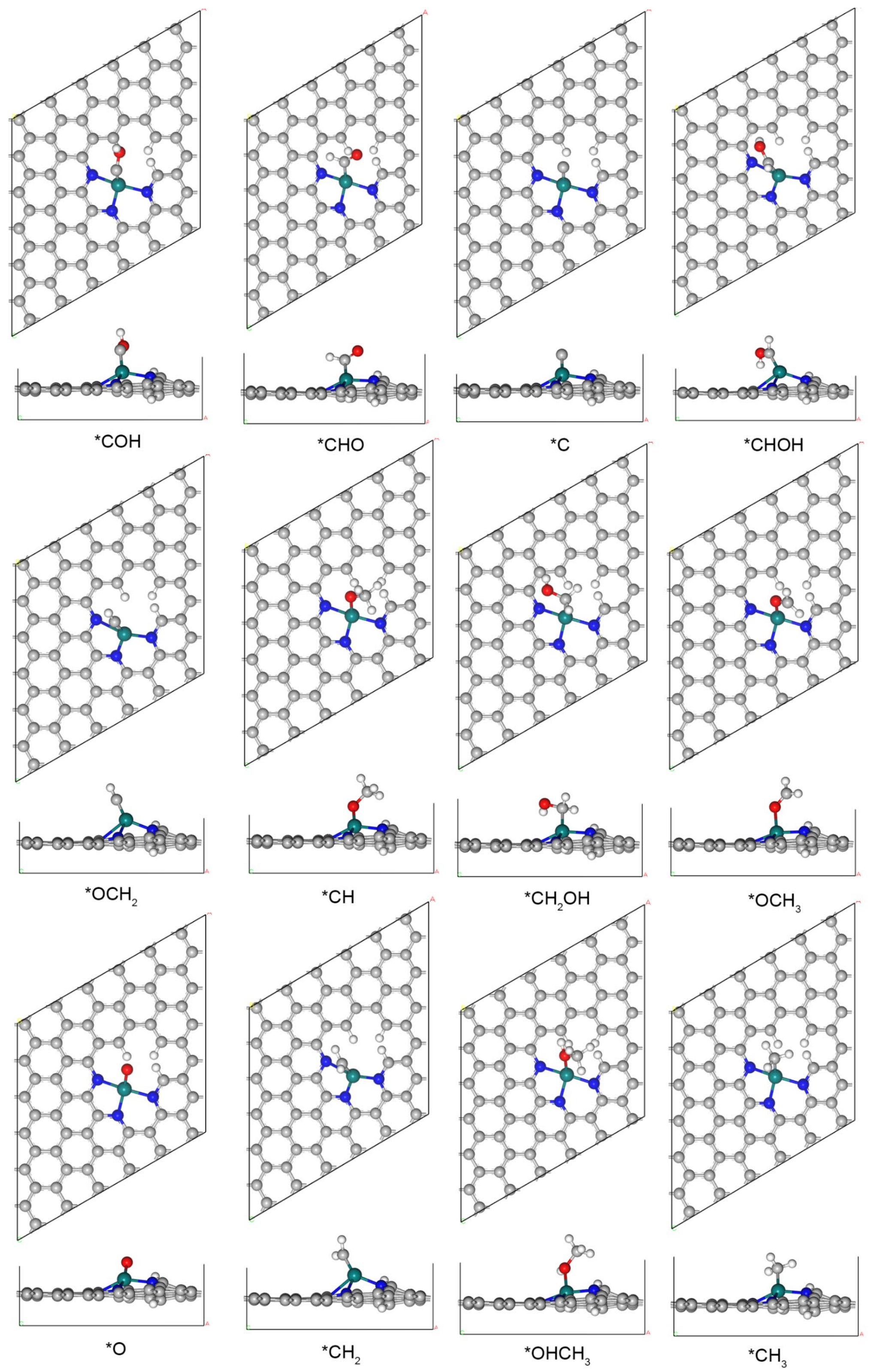

Supplementary Fig. 43. Optimized structures of CuN_3C_3 with adsorbed intermediates for CH_4 and CH_3OH productions (dark cyan for Cu, blue for N, gray for C, red for O and white for H).

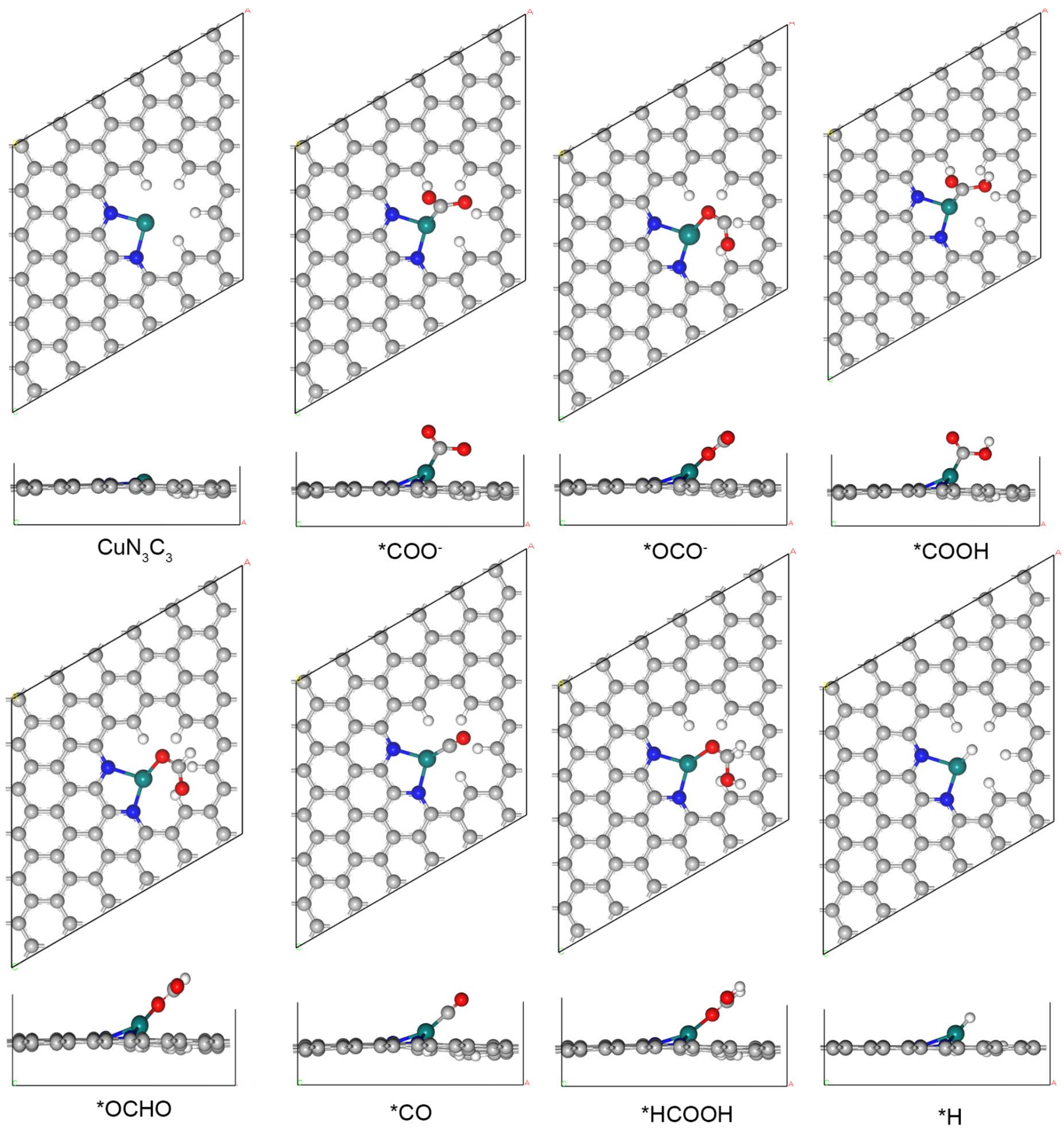

Supplementary Fig. 44. Optimized structures of CuN_2C_2 with adsorbed intermediates of *COO$^-$, *OCO$^-$, *COOH, *OCHO, *CO, *HCOOH and *H for CO, HCOOH and H_2 productions (dark cyan for Cu, blue for N, gray for C, red for O and white for H).

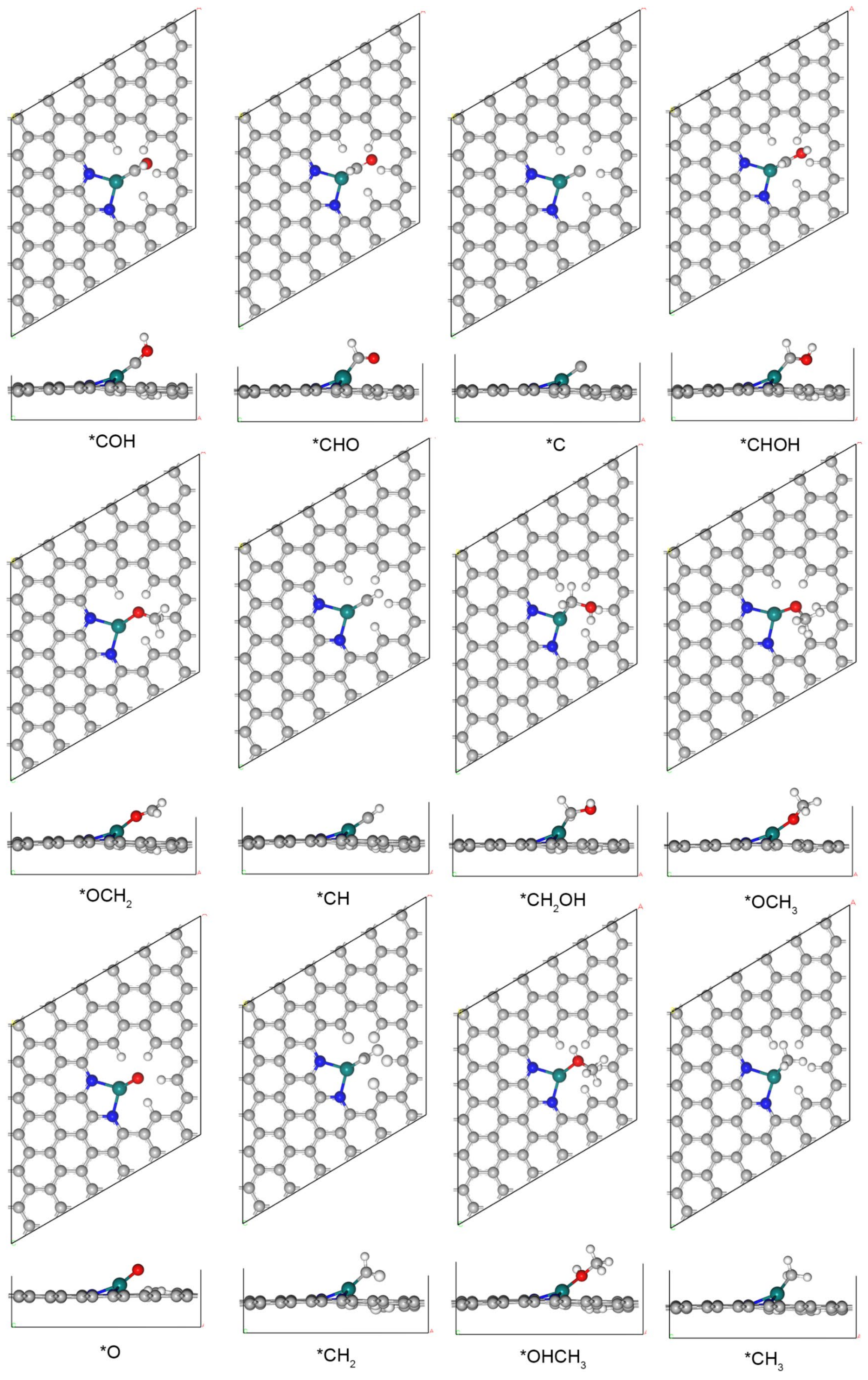

Supplementary Fig. 45. Optimized structures of CuN_2C_2 with adsorbed intermediates for CH_4 and CH_3OH productions (dark cyan for Cu, blue for N, gray for C, red for O and white for H).

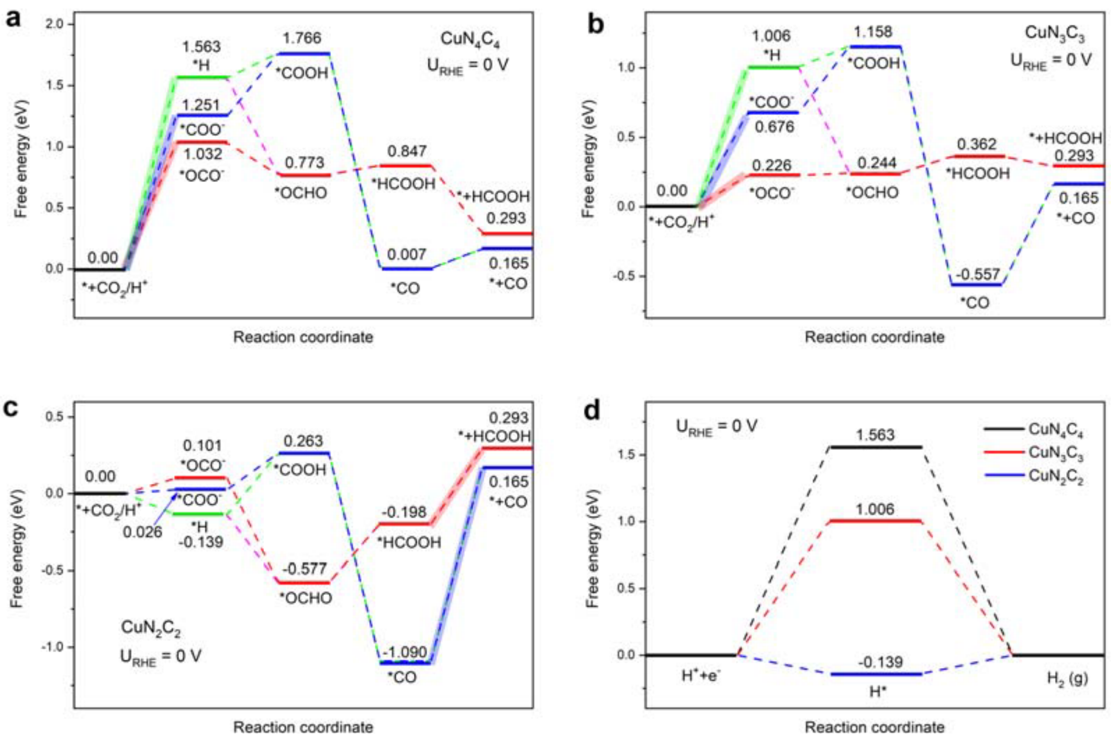

Supplementary Fig. 46. a-c, Gibbs free-energy diagrams for CO_2 electroreduction to CO and HCOOH on (**a**) CuN_4C_4, (**b**) CuN_3C_3 and (**c**) CuN_2C_2 structures at zero electrode potential. **d**, Gibbs free-energy diagrams for HER at zero electrode potential. The numbers represent the free energies for the adsorbed states. The highlights indicate the potential limiting steps.

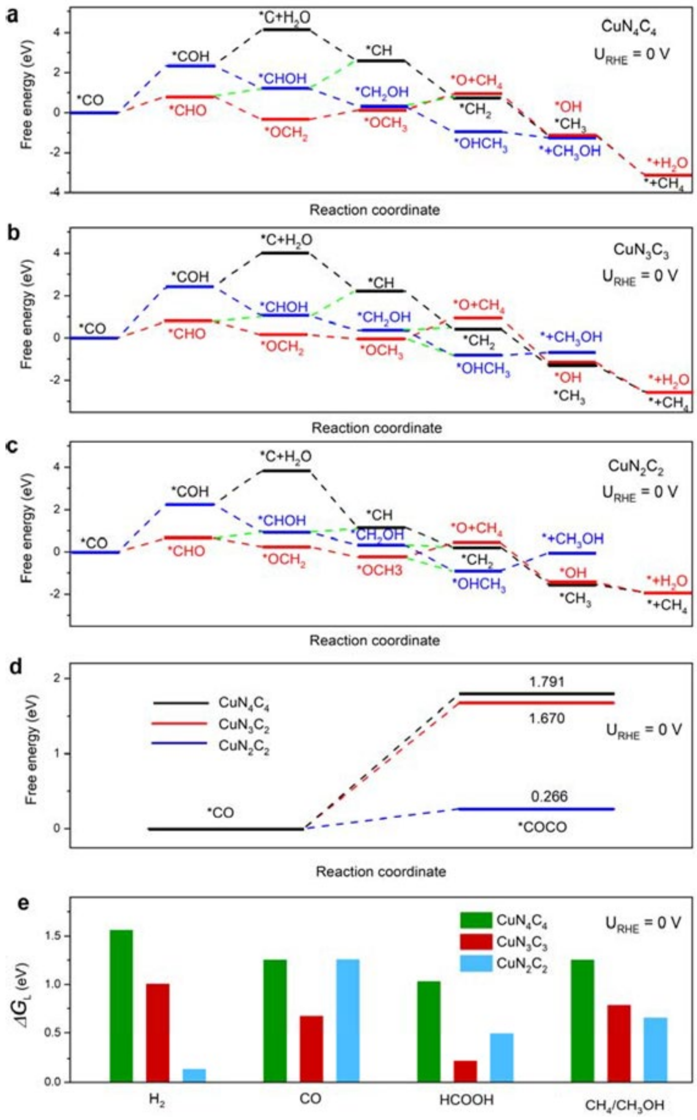

Supplementary Fig. 47. a-c, Free-energy diagrams for CO_2 electroreduction to CH_4 and CH_3OH products on CuN_4C_4, CuN_3C_3 and CuN_2C_2 structures at zero electrode potential. The highlights indicate the potential limiting steps. **d**, The ΔG values for the first C–C coupling of two CO* species at zero electrode potential. **e**, Magnitudes of the theoretical limiting free energy differences (ΔG_L) for CO, HCOOH, H_2 and CH_4/CH_3OH productions at zero electrode potential.

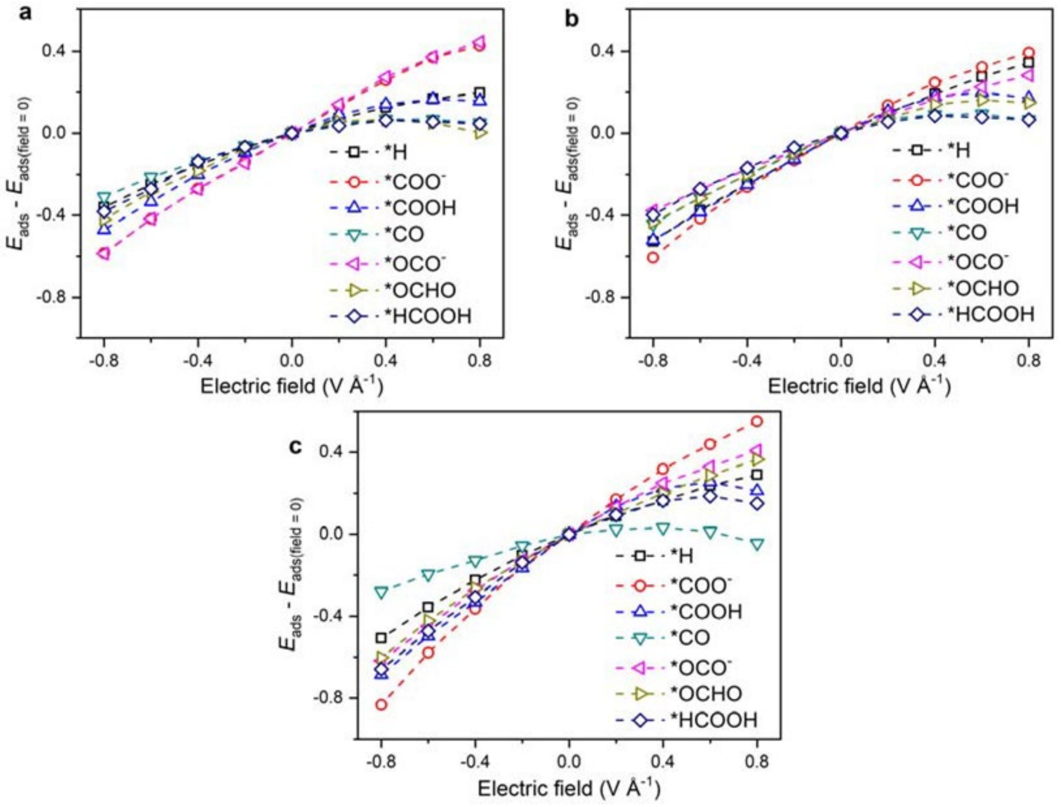

Supplementary Fig. 48. a-c, Electric field effects on the adsorbate bindings on (**a**) CuN_4C_4, (**b**) CuN_3C_3 and (**c**) CuN_2C_2 structures.

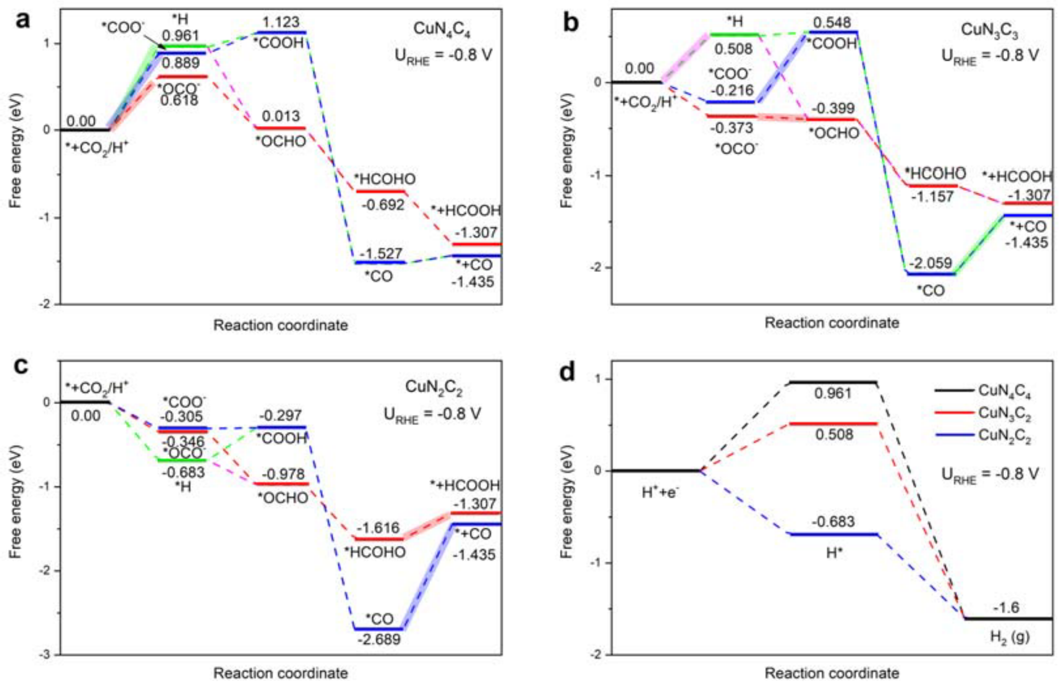

Supplementary Fig. 49. a-c, Gibbs free-energy diagrams for CO_2 electroreduction to CO, HCOOH and H_2 on (**a**) CuN_4C_4, (**b**) CuN_3C_3 and (**c**) CuN_2C_2 structures at an applied potential of -0.80 V vs. RHE. The numbers represent the free energies for the adsorbed states. The highlights indicate the potential limiting steps.

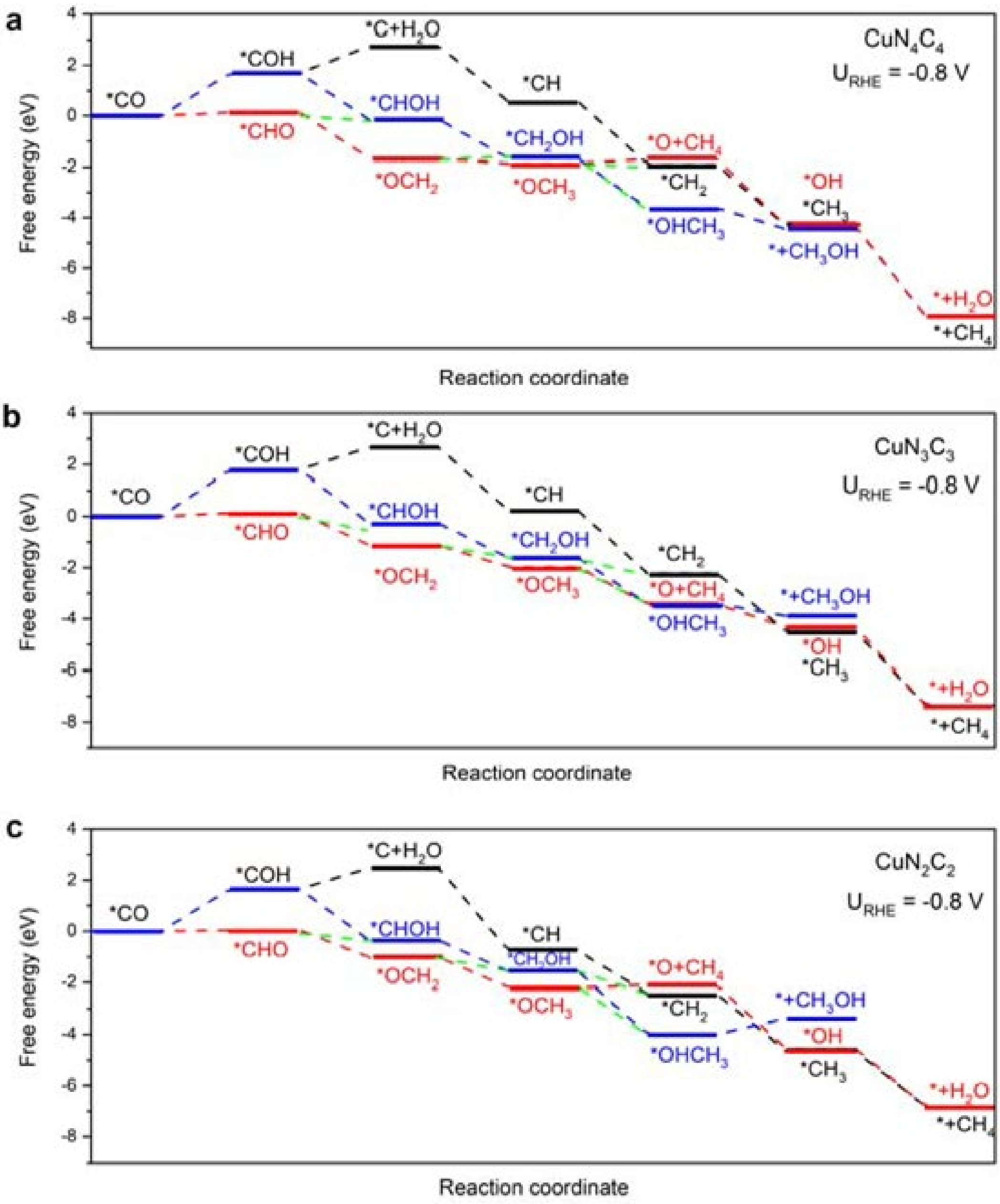

Supplementary Fig. 50. a-c, Free-energy diagrams for CO_2 electroreduction to CH_4 and CH_3OH products on (**a**) CuN_4C_4, (**b**) CuN_3C_3 and (**c**) CuN_2C_2 structures at an applied potential of -0.80 V vs. RHE.

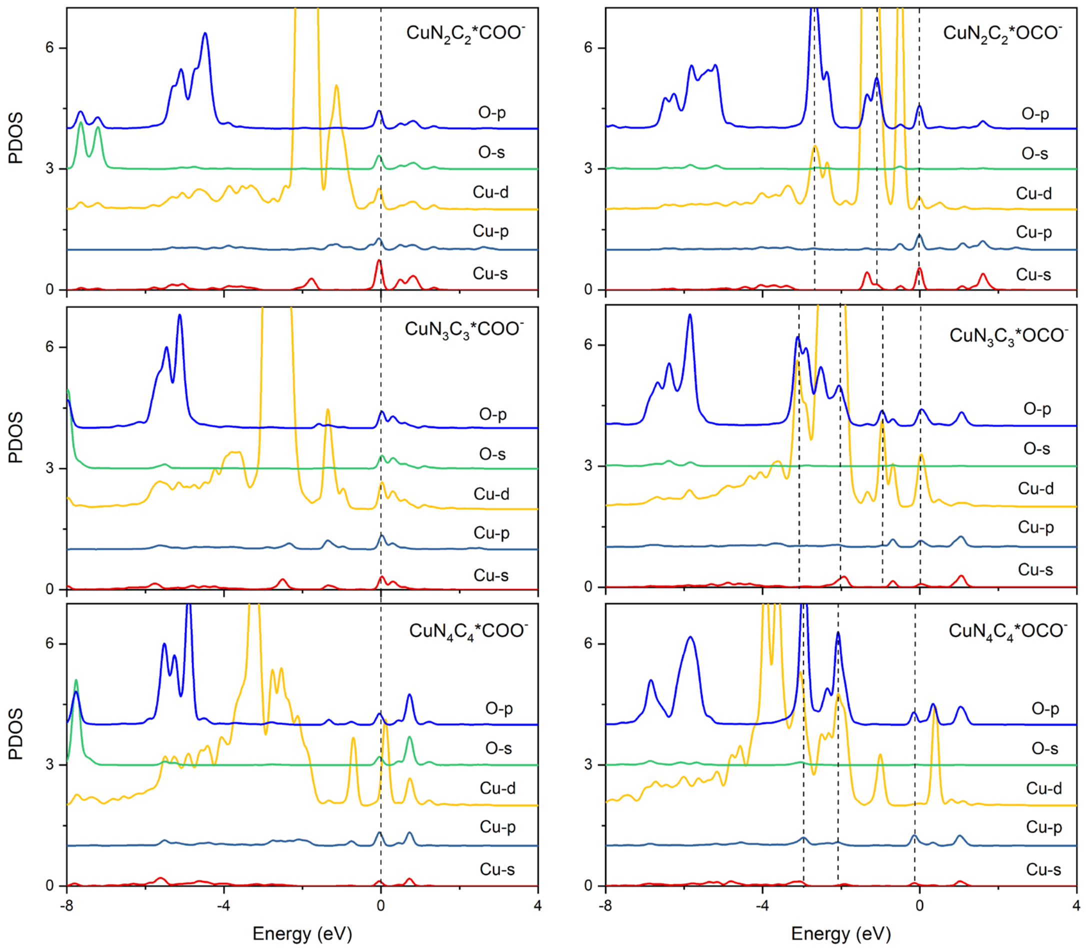

Supplementary Fig. 51. Projected density of states plots of the 4*s*, 4*p* and 3*d* orbitals for the single Cu atom and the 2*s* and 2*p* orbitals for the bound C/O atom in the adsorbed states of *COO⁻ and *OCO⁻ supported on different CuN_xC_y structures. The black dashed lines highlight the overlap between Cu-*d* and O/C-*p* orbitals. For the adsorbed intermediates of *COO⁻ and *OCO⁻, the CO_2 binds to the Cu centre through the C and O atoms, respectively.

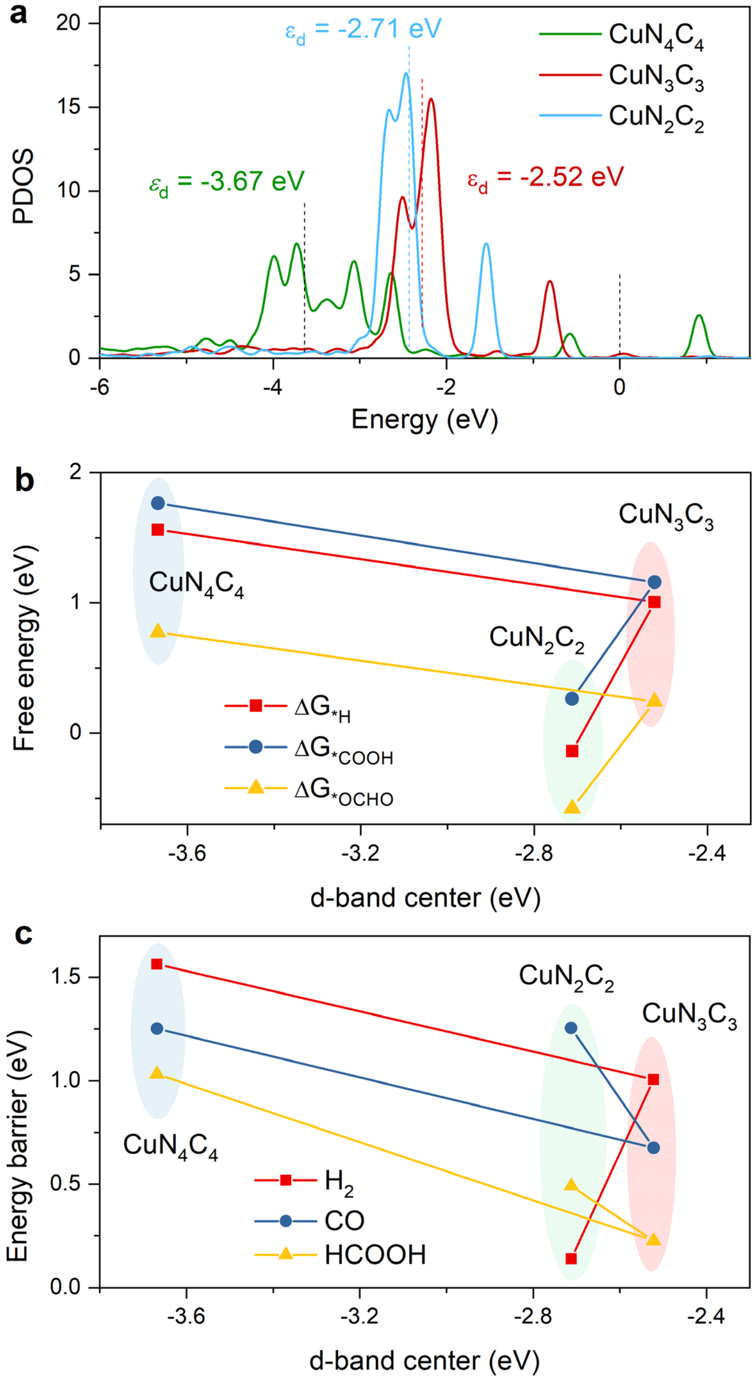

Supplementary Fig. 52. **a**, Calculated PDOS and *d*-band centres for Cu atom in CuN_4C_4, CuN_3C_3 and CuN_2C_2. **b**,**c**, The plots of free energy (**b**) and theoretical limiting free energy differences (ΔG_L) (**c**) versus d-band centre.

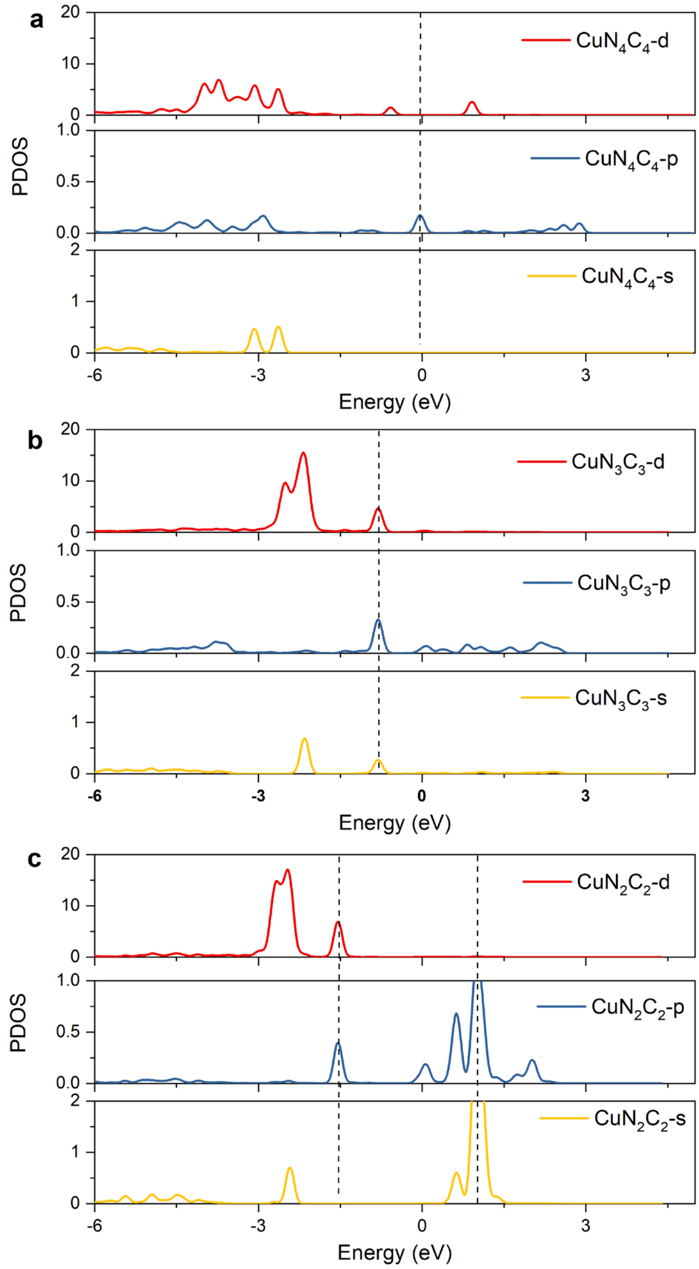

Supplementary Fig. 53. a-c, Calculated PDOS for Cu atom in CuN_4C_4 (**a**), CuN_3C_3 (**b**) and CuN_2C_2 (**c**).

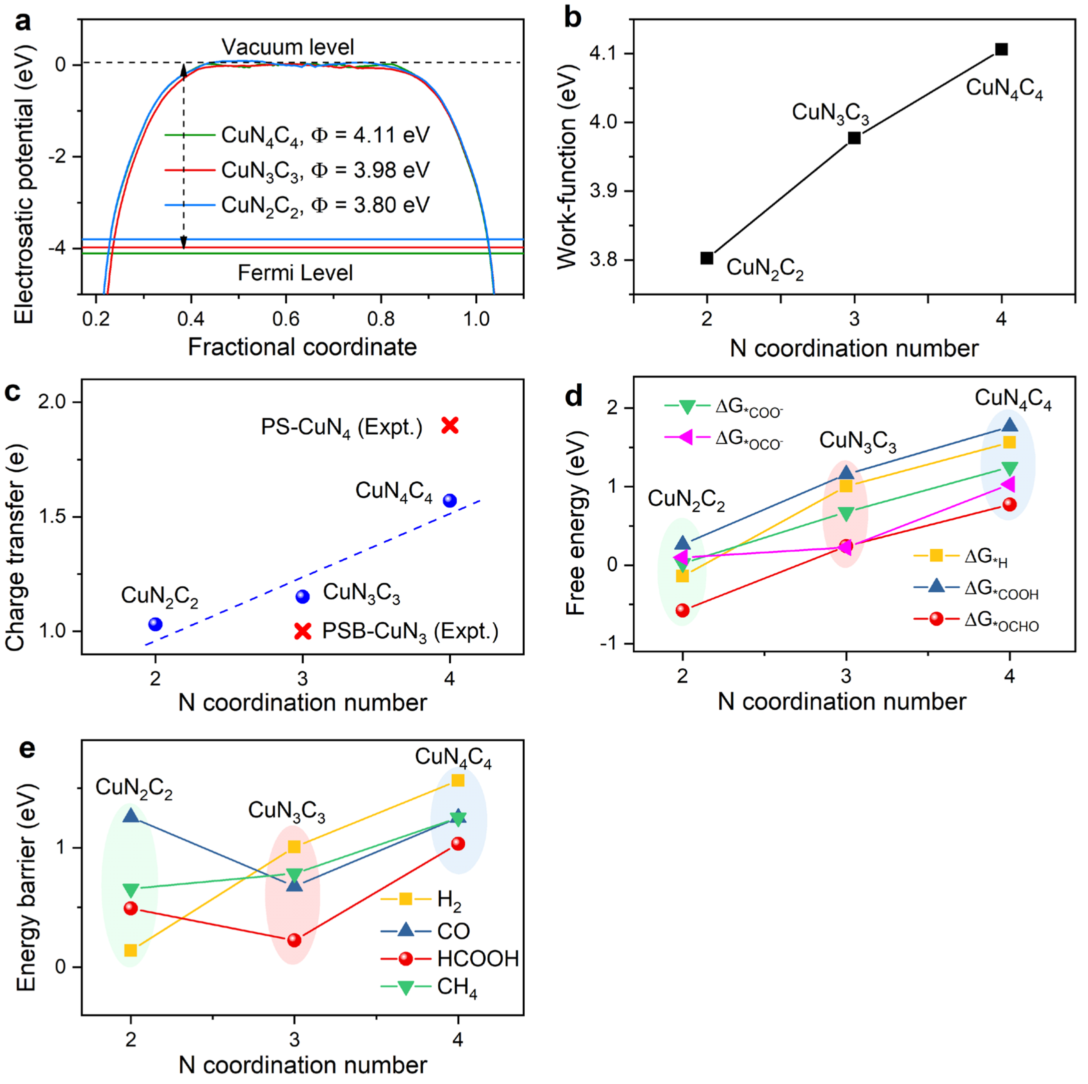

Supplementary Fig. 54. a, Calculated work-function of CuN_3C_3, CuN_4C_4 and CuN_2C_2. **b**, Work-function versus the coordination number of nearest-neighbouring N atoms around the centre Cu. **c**, Total charge transfer from Cu in different structures. **d**, Adsorption free energy versus the coordination number of nearest-neighbouring N atoms around the centre Cu. **e**, Theoretical limiting free energy differences (ΔG_L) versus the coordination number of nearest-neighbouring N atoms around the centre Cu.

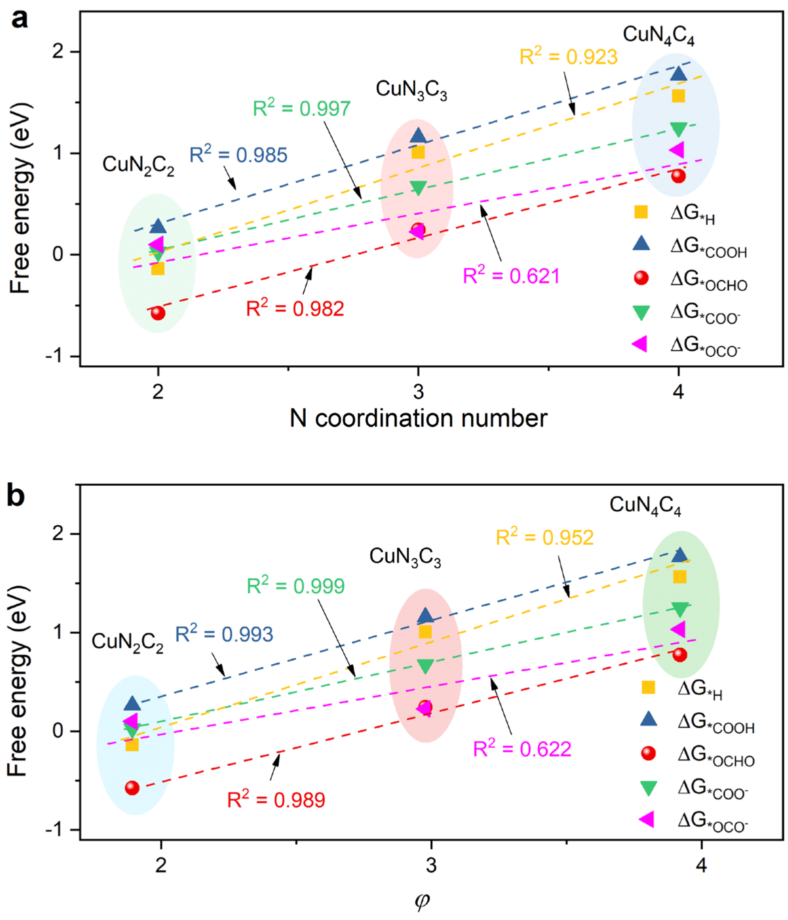

Supplementary Fig. 55. a, Adsorption free energy of ΔG_{*OCO^-}, ΔG_{*COO^-}, ΔG_{*OCHO}, ΔG_{*COOH} and ΔG_{*H} versus the coordination number of nearest-neighbouring N atoms around the centre Cu. **b**, Adsorption free energies of ΔG_{*OCO^-}, ΔG_{*COO^-}, ΔG_{*OCHO}, ΔG_{*COOH} and ΔG_{*H} versus the new descriptor φ. R^2 factor indicates the goodness of the fit. It is shown that the linearity between the new descriptor φ and the adsorption energies of ΔG_{*OCO^-}, ΔG_{*COO^-}, ΔG_{*OCHO}, ΔG_{*COOH} and ΔG_{*H} can be improved appreciably as compared to the usage of only CN, thus confirming the propriety and convenience of this new descriptor φ.

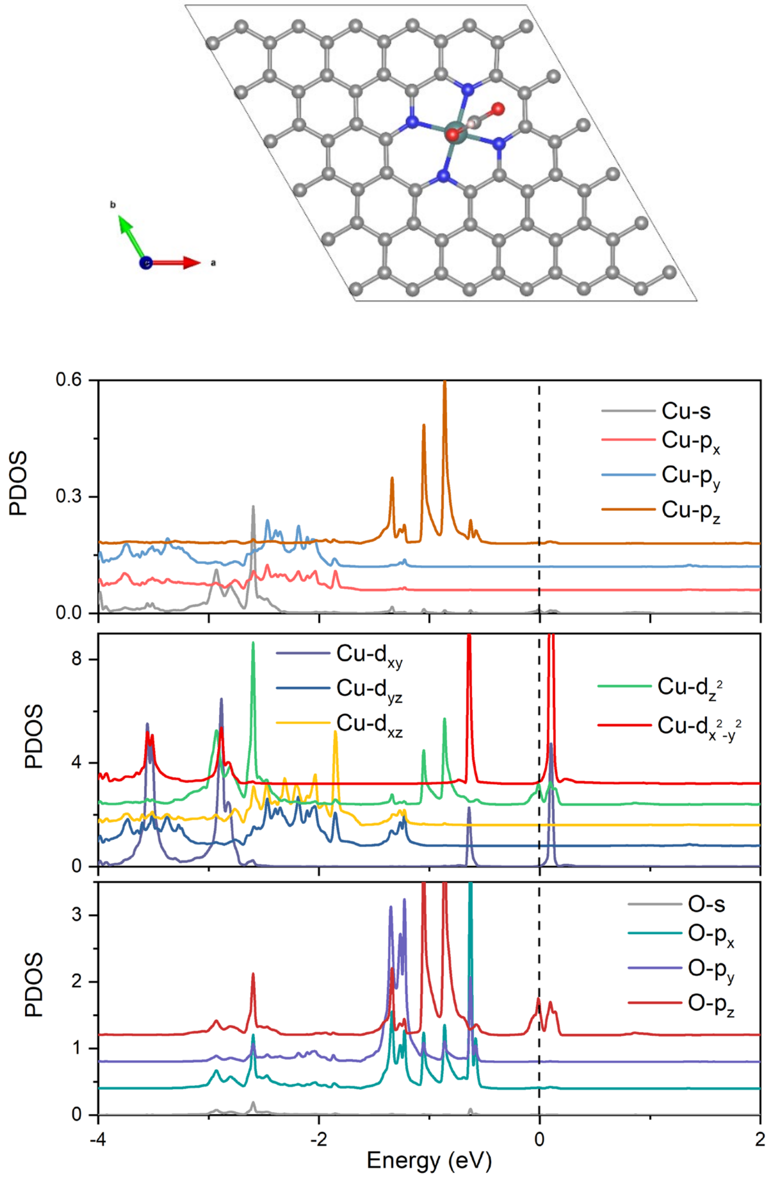

Supplementary Fig. 56. Projected density of states (PDOS) of the 4*s*, 4*p* and 3*d* orbitals for the single Cu atom and the 2*s* and 2*p* orbitals for the bound O atom in the adsorbed state of *OCHO supported on CuN_4C_4 structure. The black dashed line represents the Femi energy level.

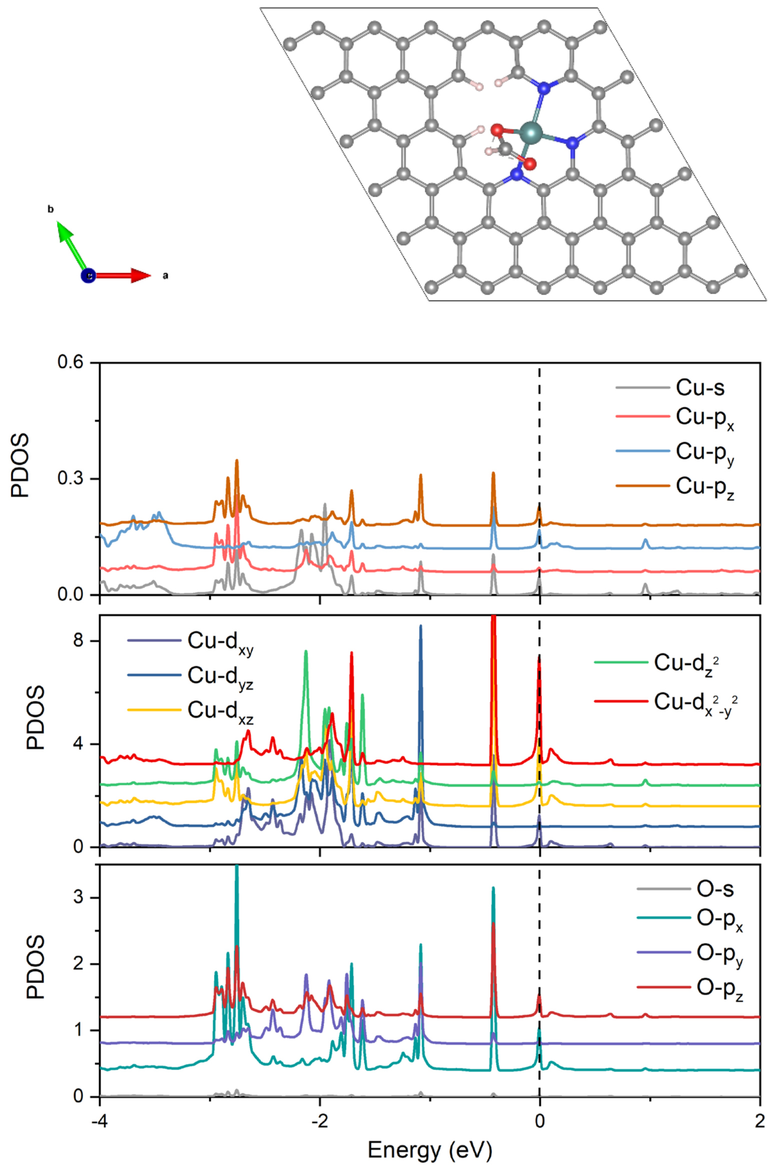

Supplementary Fig. 57. Projected density of states (PDOS) of the 4*s*, 4*p* and 3*d* orbitals for the single Cu atom and the 2*s* and 2*p* orbitals for the bound O atom in the adsorbed state of *OCHO supported on CuN_3C_3 structure. The black dashed line represents the Femi energy level.

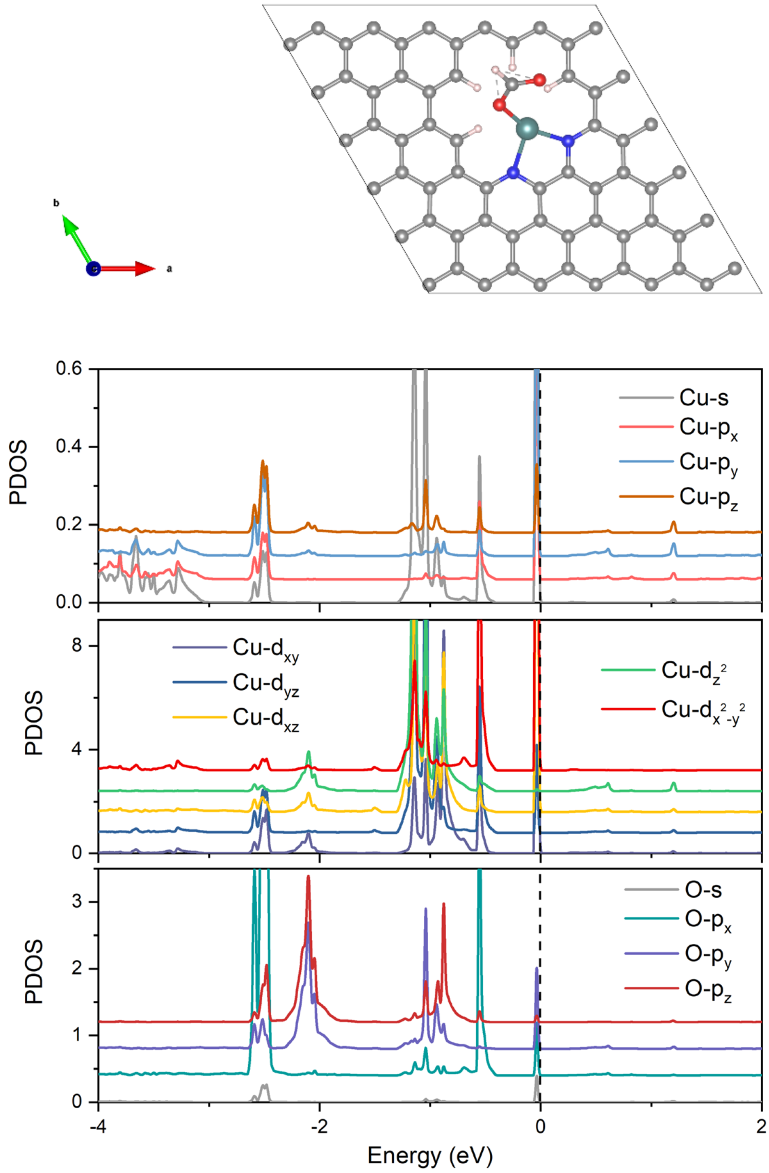

Supplementary Fig. 58. Projected density of states (PDOS) of the 4*s*, 4*p* and 3*d* orbitals for the single Cu atom and the 2*s* and 2*p* orbitals for the bound O atom in the adsorbed state of *OCHO supported on CuN_2C_2 structure. The black dashed line represents the Femi energy level.

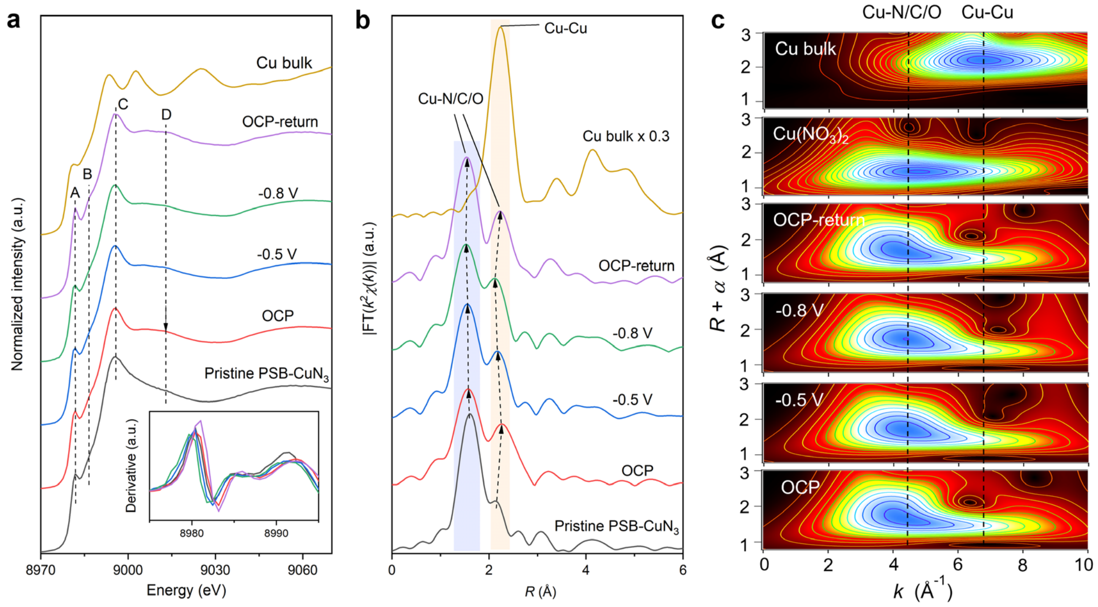

Supplementary Fig. 59. Operando Cu K-edge XAFS spectra for PSB-CuN_3 recorded at various applied potentials vs. RHE in 0.5 M $KHCO_3$ aqueous solution. **a**, XANES spectra at different potentials. **b**, Fourier-transformed magnitudes of the EXAFS spectra at different potentials. **c**, EXAFS wavelet transforms at different potentials.

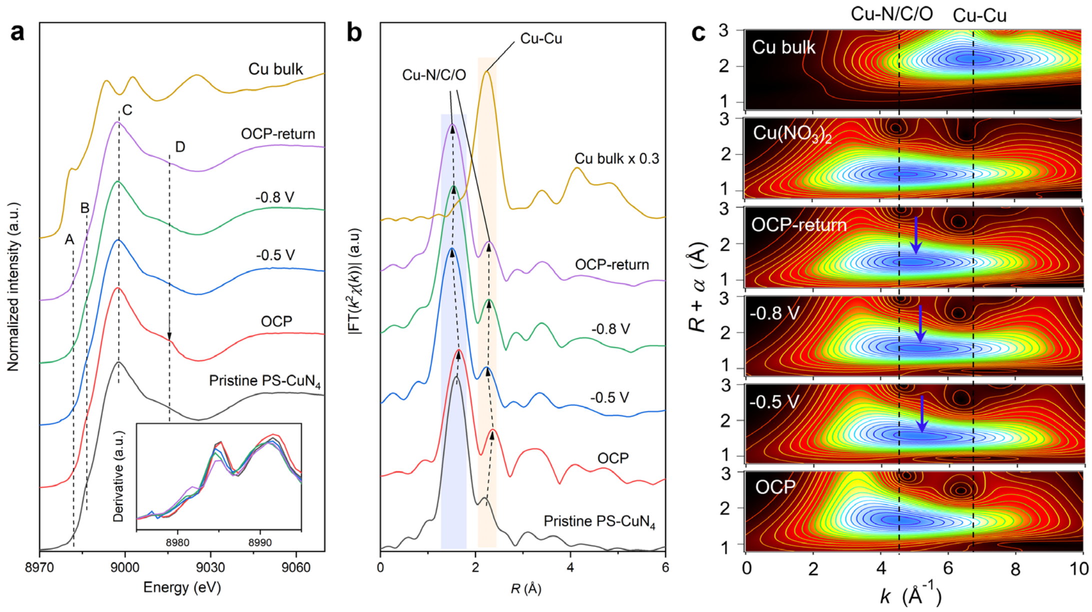

Supplementary Fig. 60. Operando Cu K-edge XAFS spectra for PS-CuN_4 recorded at various applied potentials vs. RHE in 0.5 M $KHCO_3$ aqueous solution. **a**, XANES spectra at different potentials. **b**, Fourier-transformed magnitudes of the EXAFS spectra at different potentials. **c**, EXAFS wavelet transforms at different potentials.

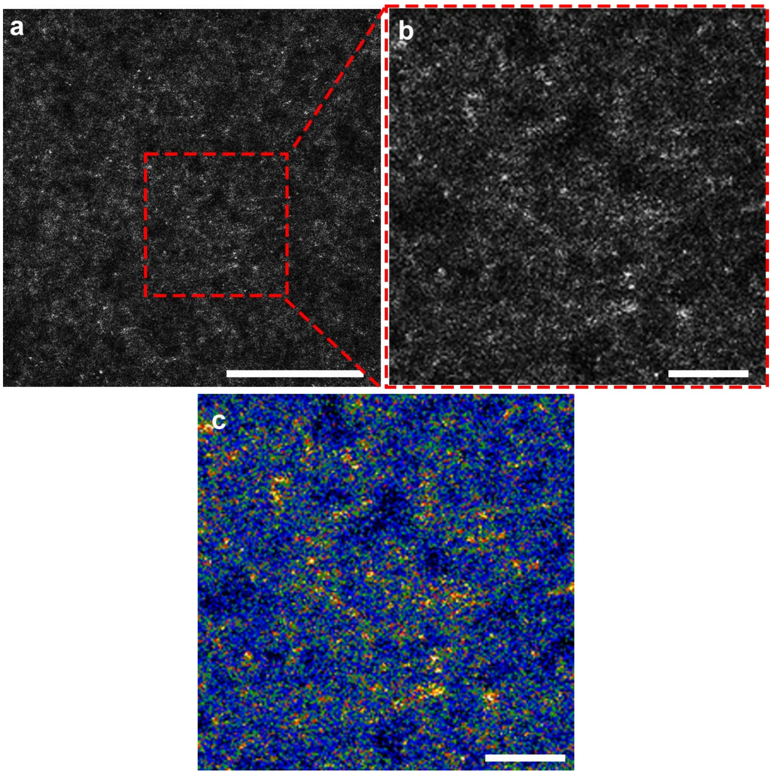

Supplementary Fig. 61. a-c, AC-HAADF-STEM image (scale bar, 5 nm; **a**), enlarged image (scale bar, 1 nm; **b**) and corresponding intensity maps (scale bar, 1 nm; **c**) of PSB-CuN_3 after CO_2RR test.

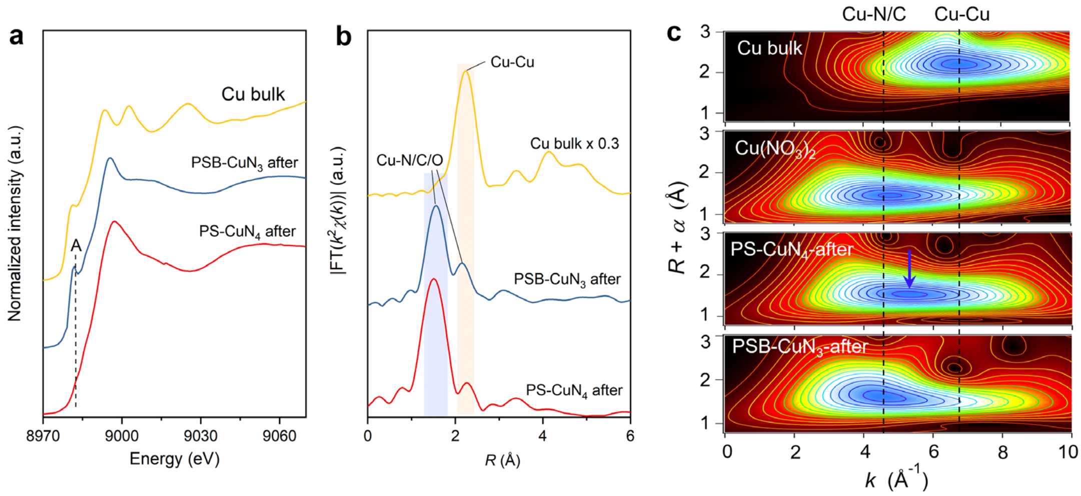

Supplementary Fig. 62. Comparison of the experimental Cu K-edge XAFS spectra for PSB-CuN_3 and PS-CuN_4 after CO_2RR test along with Cu bulk reference. **a**, XANES spectra. **b**, Fourier-transformed magnitudes of the EXAFS spectra. **c**, EXAFS wavelet transforms.

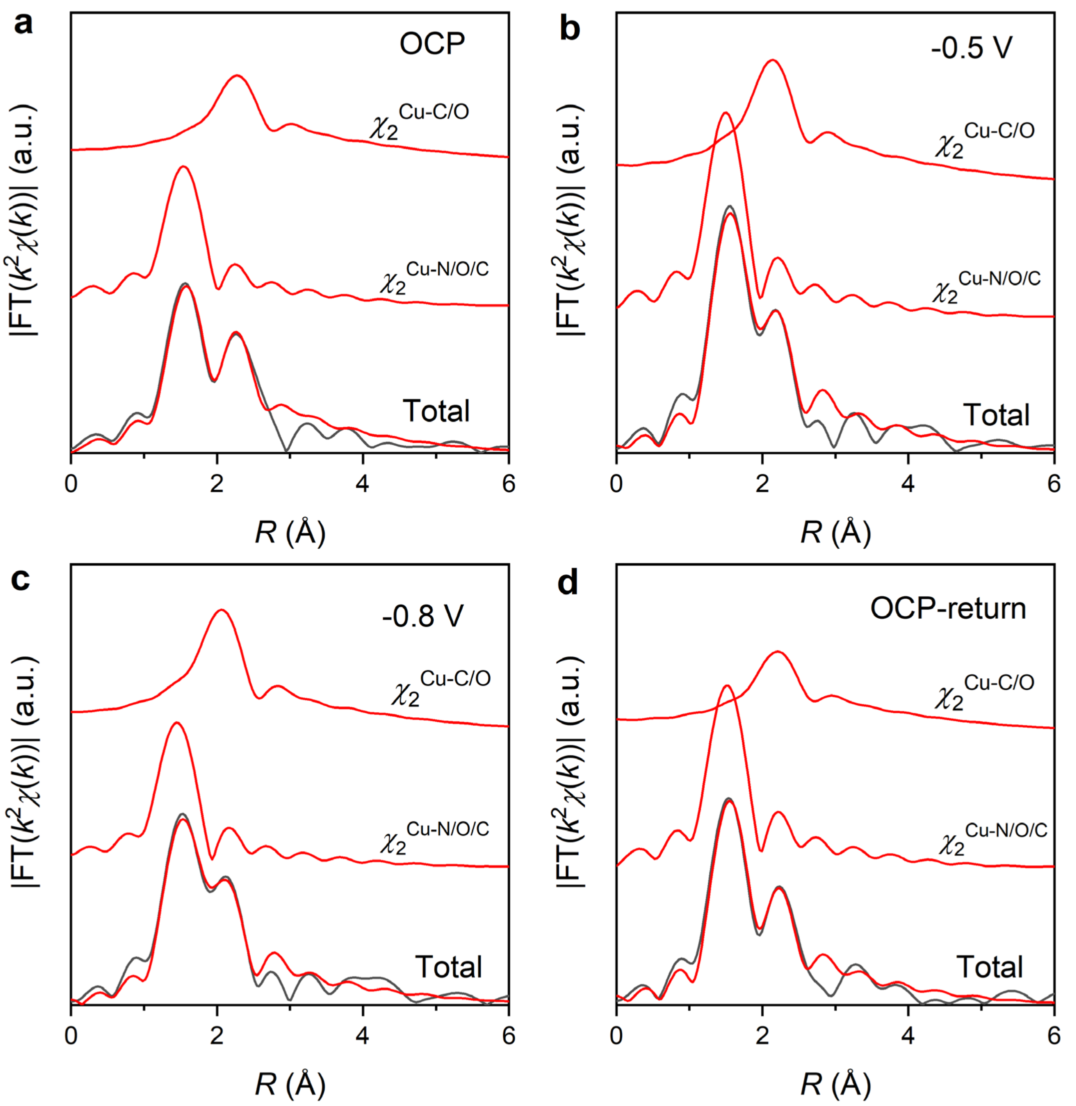

Supplementary Fig. 63. The fourier-transformed magnitudes of the operando Cu K-edge EXAFS curve-fitting in R space for PSB-CuN_3 sample at various applied potentials vs. RHE. The calculated (red line) and measured (black line) spectra show excellent agreement, validating the usage of the Cu–N and Cu–C two-body backscattering paths in the fit.

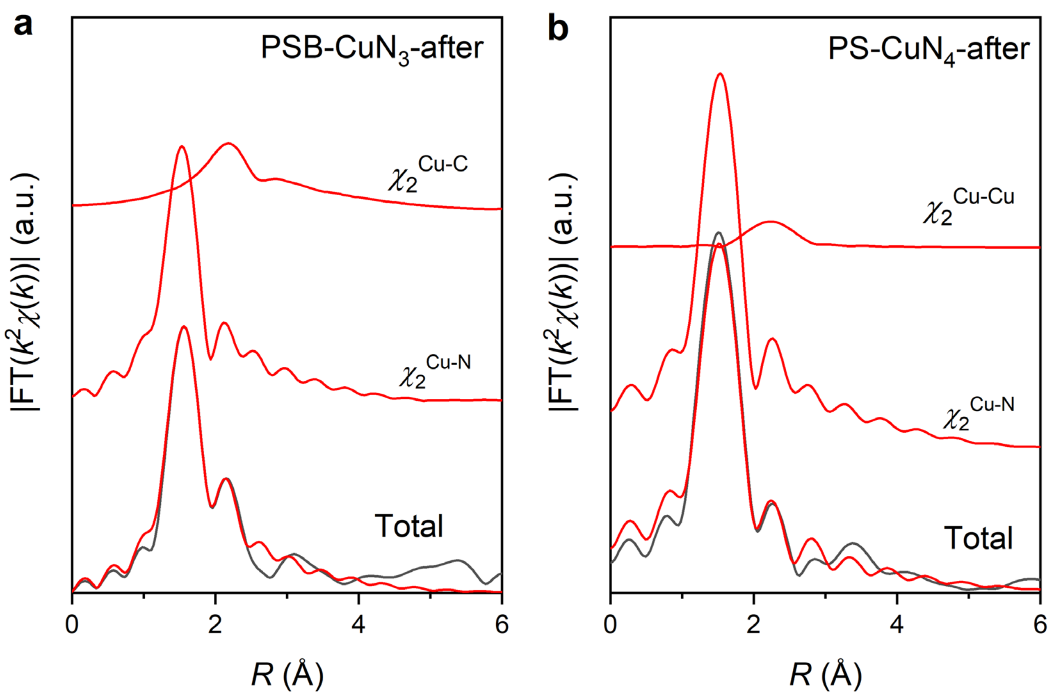

Supplementary Fig. 64. The fourier-transformed magnitudes of the Cu K-edge EXAFS curve-fitting in R space for PSB-CuN_3 and PS-CuN_4 samples after CO_2RR test.

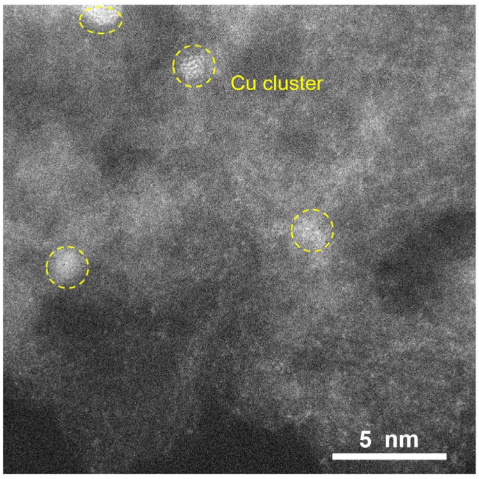

Supplementary Fig. 65. AC-HAADF-STEM image (scale bar, 5 nm) of PS-CuN_4 after CO_2RR test.

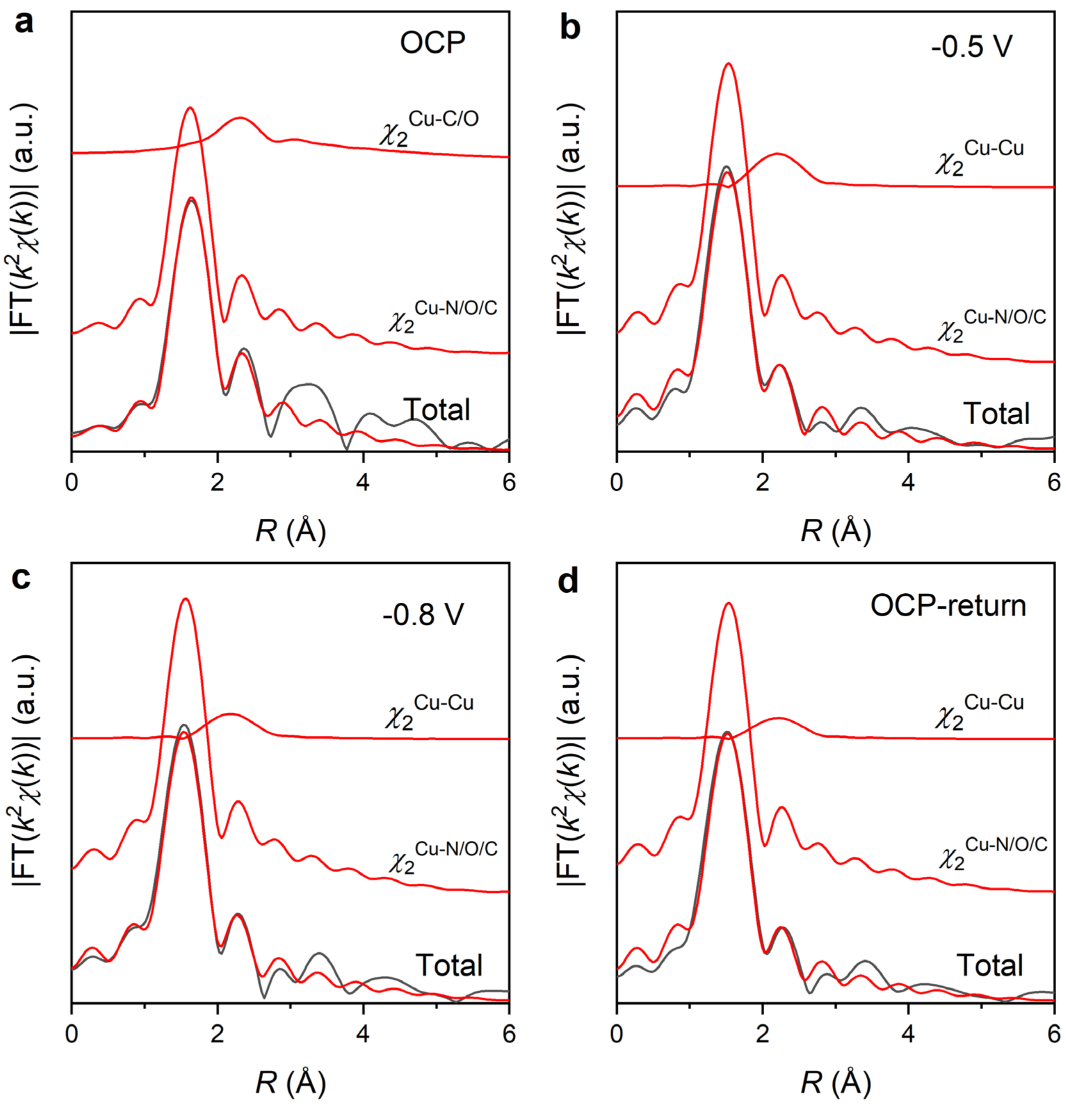

Supplementary Fig. 66. The fourier-transformed magnitudes of the operando Cu K-edge EXAFS curve-fitting in R space for PS-CuN_4 sample at various applied potentials. In contrast to the OCP state, a Cu–Cu two-body backscattering path is included in the fit at -0.5 V vs. RHE, -0.8 V vs. RHE and OCP-return, and the excellent agreement between the calculated (red line) and measured (black line) spectra confirm the formation of fractional Cu-Cu nanocluster.

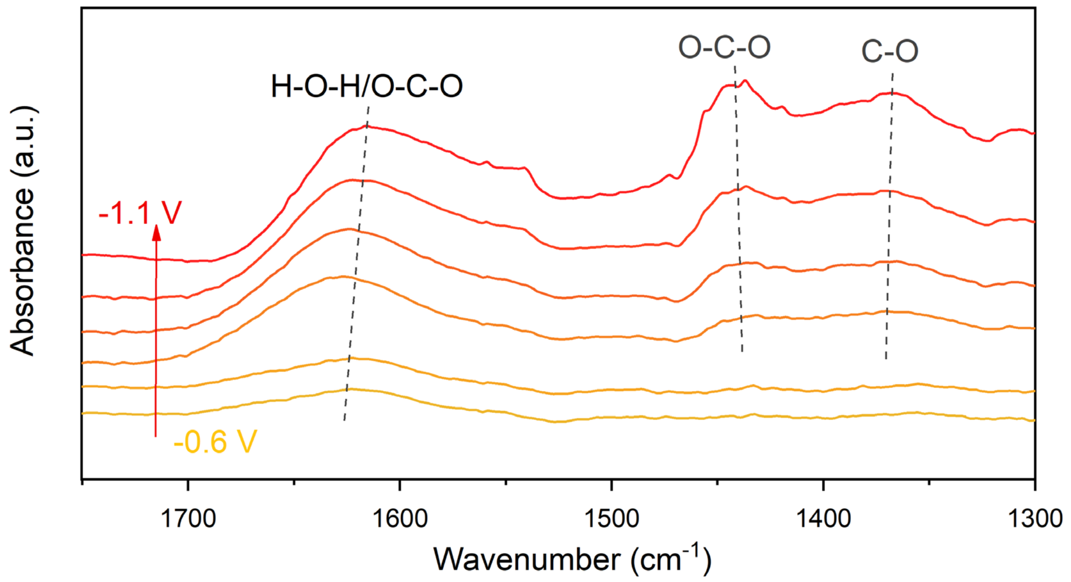

Supplementary Fig. 67. Operando ATR-SEIRAS spectra PSB-CuN_3 recorded at various applied potentials vs. RHE in 0.5 M $KHCO_3$ aqueous solution.

Supplementary Table 1. Cu *K*-edge EXAFS curve fitting parameters.[a]

sample	shell	*N*	*R* (Å)	σ^2 (Å^2)	ΔE_0 (eV)	*R*, %
Cu bulk[b]	Cu−Cu	**12**	2.54	0.008	1.3	0.01
Cu_2O[c]	Cu−O<br>Cu−Cu<br>Cu−O	**2**<br>**12**<br>**6**	1.86<br>3.04<br>3.62	0.003<br>0.022<br>0.013	4.6	0.02
$Cu(NO_3)_2$[d]	Cu−O<br>Cu−N/O	4.8<br>5.2	1.98<br>2.70	0.004<br>0.023	-3.1	0.3
PS-CuN_4[d]	Cu−N<br>Cu−C	4.0<br>4.1	2.00<br>2.67	0.003<br>0.014	5.9	0.2
PS-CuN_4-OCP[e]	Cu−N/O/C<br>Cu−C/O	4.3<br>4.5	2.03<br>2.79	0.004<br>0.013	8.3	0.3
PS-CuN_4 at -0.5 V[e]	Cu−N/O/C<br>Cu−Cu	5.2<br>0.6	1.98<br>2.60	0.004<br>0.008	5.9<br>-5.3	0.3
PS-CuN_4 at -0.8 V[e]	Cu−N/O/C<br>Cu−Cu	5.0<br>0.7	2.00<br>2.60	0.004<br>0.008	7.5<br>-8.7	0.2
PS-CuN_4-OCP-return[e]	Cu−N/O/C<br>Cu−Cu	4.9<br>0.4	1.98<br>2.60	0.004<br>0.008	6.1<br>-4.6	0.4
PS-CuN_4-after[e]	Cu−N<br>Cu−Cu	4.9<br>0.4	1.98<br>2.60	0.004<br>0.008	5.9<br>-2.0	0.3
PSB-CuN_3[d]	Cu−N<br>Cu−C	2.8<br>2.9	1.98<br>2.65	0.004<br>0.014	6.8	0.3
PSB-CuN_3-OCP[e]	Cu−N/O/C<br>Cu−C/O	3.6<br>4.2	1.98<br>2.78	0.006<br>0.012	6.6	0.3
PSB-CuN_3 at -0.5 V[e]	Cu−N/O/C<br>Cu−C/O	3.8<br>4.1	1.93<br>2.66	0.003<br>0.013	6.2	0.4
PSB-CuN_3 at -0.8 V[e]	Cu−N/O/C<br>Cu−C/O	4.0<br>4.2	1.90<br>2.61	0.003<br>0.013	3.6	0.3
PSB-CuN_3-OCP-return[e]	Cu−N/O/C<br>Cu−C/O	3.7<br>3.9	1.96<br>2.70	0.003<br>0.013	6.9	0.3
PSB-CuN_3-after[d]	Cu−N<br>Cu−C	3.1<br>3.3	1.96<br>2.71	0.003<br>0.013	6.5	0.4

[a]*N*, coordination number; *R*, distance between absorber and backscatter atoms; σ^2, Debye–Waller factor to account for both thermal and structural disorders; ΔE_0, inner potential correction; *R* factor (%) indicates the goodness of the fit. Error bounds (accuracies) that characterize the structural parameters obtained by EXAFS spectroscopy were estimated as $N \pm 20\%$; $R \pm 1\%$; $\sigma^2 \pm 20\%$; $\Delta E_0 \pm 20\%$. S_0^2 were determined from $Cu(NO_3)_2$ reference fitting and fixed. Bold numbers indicate fixed coordination number (*N*) according to the crystal structure. [b]Fitting range: $2.9 \leq k$ (/Å) ≤ 12.5 and $1.0 \leq R$ (Å) ≤ 2.8. [c]Fitting range: $2.9 \leq k$ (/Å) ≤ 12.5 and $1.0 \leq R$ (Å) ≤ 3.6. [d]Fitting range: $2.5 \leq k$ (/Å) ≤ 10.2 and $1.0 \leq R$ (Å) ≤ 2.8. [e]Fitting range: $2.8 \leq k$ (/Å) ≤ 9.1 and $1.0 \leq R$ (Å) ≤ 2.8.

Supplementary Table 2. The formation energy of various CuN_xC_y model structures.

	ΔE_F	E_{sys}	n_C	μ_C	n_N	μ_N	n_H	μ_H	μ_{Cu}
CuN_4C_4	1.211	-12475.168	66		4		0		
$Cu(N_3C)C_4$	2.408	-12359.439	67		3		0		
$Cu(N_2C_2)C_4$-1	3.300	-12244.015	68		2		0		
$Cu(N_2C_2)C_4$-2	3.555	-12243.760	68	-154.846	2	-269.378	0	-15.851	-1179.031
$Cu(NC_3)C_4$	4.191	-12128.592	69		1		0		
CuN_3C_3	2.063	-12097.645	65		3		3		
CuN_2C_2	2.289	-11689.046	64		2		4		

Supplementary Table 3. The FEs of all products in the whole potential interval.

Potential (V vs. RHE)	$FE_{formate}$ (%)			FE_{CH4} (%)			FE_{CO} (%)			FE_{H2} (%)		
	PSB-CuN_3	PS-CuN_4	AG Ns	PSB-CuN_3	PS-CuN_4	AG Ns	PSB-CuN_3	PS-CuN_4	AG Ns	PSB-CuN_3	PS-CuN_4	AG Ns
-0.431	26.8	-	-	12.7	0	-	8.4	-	-	30.1	-	-
-0.531	43.3	7.5	-	27.2	9.8	-	14.6	10.2	-	10.2	49.8	-
-0.631	73.5	25.9	3.3	16.2	15.1	-	4.5	5.1	16.9	6.4	42.7	60.4
-0.731	94.3	33.6	3.9	3.2	16.0	-	1.1	2.8	28.4	2.1	41.9	58.3
-0.831	93.6	62.5	4.9	-	6.4	-	-	2.0	30.6	3.6	30.2	58.2
-0.931	92.7	72.4	9.5	-	-	-	-	-	26.8	5.9	24.8	62.6
-1.031	90.2	69.2	6.3	-	-	-	-	-	15.3	8.7	29.5	79.4
-1.131	87.8	51.3	5.4	-	-	-	-	-	7.6	11.2	30.1	86.4

Note: "-" represents that the product is lower than the minimum detection limit of ^{1}H NMR spectra and on-line GC.

Supplementary Table 4. The FEs of different products in a flow-cell setup under different potentials on PSB-CuN_3 catalyst.

Potential (V vs. RHE)	$FE_{formate}$ (%)	FE_{CH4} (%)	FE_{CO} (%)	FE_{H2} (%)
-0.37	74.5	-	-	9.8
-0.47	85.3	2.3	2.2	6.7
-0.57	89.2	4.7	3.5	2.8
-0.67	94.5	3.5	1.4	2.6
-0.77	97.1	-	1.2	1.8
-0.87	97.9	-	-	2.2
-0.97	96.4	-	-	5.2
-1.07	84.0	-	-	10.9

Note: "-" represents that the product is lower than the minimum detection limit of on-line GC.

Supplementary Table 5. Comparison of catalytic performance between PSB-CuN_3 and recently reported catalysts for electrochemical reduction CO_2 to formate.

Catalyst	Electrolyte	Operating potential (V vs. RHE)	$j_{formate}$ (mA cm^{-2})	Formate FE (%)	Reference
PSB-CuN_3	0.5 M $KHCO_3$	-0.65	20	93.6	This work
		-0.85	60	97.2	
		-0.97	100	95.6	
H-InO_x NRs	0.5 M $NaHCO_3$	-0.7	5	91.7	1
Bi-dendrite	0.5 M $KHCO_3$	-0.74	10	89	2
Ultrathin Bi NSs	0.5 M $NaHCO_3$	-0.8	3.95	88.7	3
Sb SAs/NC	0.5 M $KHCO_3$	-0.8	5	94	4
Bi_2O_3/BiO_2	0.5 M $KHCO_3$	-0.8	22.4	90.76	5
Pb_1Cu SAAs	0.5M $KHCO_3$	-1.0	1000	92	6
Mn-doped In_2S_3 NSs	0.1 M $KHCO_3$	-0.9	17.2	86	7
Sn quantum sheets	0.1 M $NaHCO_3$	-1.07	18.78	89	8
Bi_2O_3@C800	1 M KOH	-0.9	186	93	9
nBuLi-Bi	1 M $KHCO_3$	-1.05	460	92	10
$ZnInO_x$/NCF	0.5 M $KHCO_3$	-1.1	14	90.5	11
SnO_2 QWs	0.1 M $KHCO_3$	-1.156	13.1	87.3	12
MC Bi_2O_3 NPs	0.5 M $NaHCO_3$	-1.2	22	91	13
Bi_2O_3 NSs	0.1 M $KHCO_3$	-1.256	16.6	93.8	14
S_2-In	0.5 M $KHCO_3$	-0.98	55.8	93	15
Bi_2O_3 double-walled nanotubes	1M KOH	-0.58	205.8	98	16
	1M $KHCO_3$	-0.86	133	95	
InP CQDs	3M KOH	-2.75	930	93	17
SPy immobilised on Cu	5M KOH	-0.43	217±5	72±2	18
SnO_2 NP	0.4 M K_2SO_4	Cell voltage: 5.9 V	450	90	19
SnO_2 on GDE	0.5 M Na_2CO_3 + 0.5 M Na_2SO_4	Cell voltage: 2.47 V	385 ± 19	72	20

Supplementary Table 6. Adsorption energies, zero-point energy and entropy corrections of $*COO^-$, $*OCO^-$, *COOH, *OCHO, *HCOOH, *CO and *H on single Cu atom supported on CuN_4C_4, CuN_3C_3 and CuN_2C_2 structures.

	ΔE_{*H} (eV)	ΔE_{*COO-} (eV)	ΔE_{*COOH} (eV)	ΔE_{*CO} (eV)	ΔE_{*OCO-} (eV)	ΔE_{*OCHO} (eV)	ΔE_{*HCOOH} (eV)
CuN_4C_4	1.423	0.933	2.081	-0.054	0.747	0.464	0.888
CuN_3C_3	0.829	0.346	1.476	-0.613	-0.065	-0.064	0.406
CuN_2C_2	-0.177	-0.318	0.540	-1.028	-0.171	-0.908	-0.073
	ΔZPE_{*H} (kcal/mol)	ΔZPE_{*COO-} (kcal/mol)	ΔZPE_{*COOH} (kcal/mol)	ΔZPE_{*CO} (kcal/mol)	ΔZPE_{*OCO-} (kcal/mol)	ΔZPE_{*OCHO} (kcal/mol)	ΔZPE_{*HCOOH} (kcal/mol)
CuN_4C_4	3.681	6.593	7.011	-1.322	5.741	5.433	-2.215
CuN_3C_3	3.025	6.634	-6.614	-0.301	6.211	4.984	-0.542
CuN_2C_2	2.117	6.806	-5.909	-1.635	6.162	5.651	-1.613
	ΔS_{*H} (cal/mol.K)	ΔS_{*COO-} (cal/mol.K)	ΔS_{*COOH} (cal/mol.K)	ΔS_{*CO} (cal/mol.K)	ΔS_{*OCO-} (cal/mol.K)	ΔS_{*OCHO} (cal/mol.K)	ΔS_{*HCOOH} (cal/mol.K)
CuN_4C_4	1.531	-2.457	0.818	-9.115	-2.800	-5.628	-4.266
CuN_3C_3	-3.539	-3.238	2.421	-5.325	-1.635	-7.035	1.544
CuN_2C_2	4.141	-3.727	1.577	-0.650	-0.338	-6.609	4.255

Supplementary Table 7. The Gibbs free energies of *COO$^-$, *OCO$^-$, *COOH, *OCHO, *HCOOH, *CO and *H and the theoretical limiting free energy differences (ΔG_L) of H_2, CO, and HCOOH productions on CuN_4C_4, CuN_3C_3 and CuN_2C_2 structures under applied potentials of U_{RHE} = 0, -0.4 and -0.8 V, pH = 1 and 7.3.

	ΔG_{H*} (eV)	$\Delta G_{COO\text{-}*}$ (eV)	ΔG_{COOH*} (eV)	ΔG_{CO*} (eV)	$\Delta G_{OCO\text{-}*}$ (eV)	ΔG_{OCHO*} (eV)	ΔG_{HCOOH*} (eV)	$E_{barrier}$ H_2 (eV)	$E_{barrier}$CO (eV)	$E_{barrier}$ HCOOH (eV)
CuN_4C_4 U=0, pH=1	1.563	1.251	1.766	0.007	1.032	0.773	0.847	1.563	1.251	1.032
CuN_4C_4 U=-0.4, pH=7.3	1.242	1.004	1.441	-0.752	0.768	0.417	0.092	1.242	1.004	0.768
CuN_4C_4 U=-0.8, pH=7.3	0.961	0.889	1.123	-1.527	0.618	0.013	-0.692	0.961	0.889	0.618
CuN_3C_3 U=0, pH=1	1.006	0.676	1.158	-0.557	0.226	0.244	0.362	1.006	0.676	0.226
CuN_3C_3 U=-0.4, pH=7.3	0.717	0.403	0.846	-1.297	-0.104	-0.084	-0.386	0.717	0.662	0.020
CuN_3C_3 U=-0.8, pH=7.3	0.508	-0.216	0.548	-2.059	-0.373	-0.399	-1.157	0.508	0.624	-0.026
CuN_2C_2 U=0, pH=1	-0.139	0.026	0.263	-1.090	0.101	-0.577	-0.198	0.139	1.255	0.491
CuN_2C_2 U=-0.4, pH=7.3	-0.440	-0.201	-0.024	-1.866	-0.168	-0.827	-0.901	-0.440	1.231	0.394
CuN_2C_2 U=-0.8, pH=7.3	-0.683	-0.395	-0.297	-2.689	-0.346	-0.978	-1.616	-0.683	1.254	0.309

Supplementary Table 8. The *d*-band centre, Bader charge transfer, work-function, modified descriptor (φ) for CuN_4C_4, CuN_3C_3 and CuN_2C_2 coordinations.

	d-band centre (eV)	Bader charge transfer of Cu site (e)	Work-function (eV)	φ
CuN_4C_4	-3.668	1.57	4.106	3.92
CuN_3C_3	-2.522	1.17	3.977	2.98
CuN_2C_2	-2.712	1.05	3.802	1.89

Supplementary Table 9. Solution resistance (R_s). charge transfer resistance (R_{ct}), and the error of PS-CuN_4 and PSB-CuN_3.

Samples	R_s	Error	R_{ct}	Error
PS-CuN_4	9.061	0.92%	11.56	0.84%
PSB-CuN_3	8.374	0.75%	9.03	0.78%

Supplementary Notes

Supplementary Note 1.

Various $Cu(N_{4-x}C_x)C_4$ structures with N atoms in the first coordination sphere substituted with C atoms that include $Cu(N_3C_1)C_4$, $Cu(N_2C_2)C_4$ and $Cu(N_1C_3)C_4$ are constructed and compared in the Cu K-edge and $L_{3,2}$-edge XANES simulations (see Supplementary Fig. 24). When compared to the theoretical Cu K-edge spectra of pure CuN_4C_4, the substitution of N with C atom, i.e., $Cu(N_3C_1)C_4$, leads to an emergence of a pre-edge peak A at ~ 8979 eV and this peak intensity continues to show a gradual increase with the increased amount of C substitution (Supplementary Fig. 24b). By contrast, other peaks (e.g., B, C and D) only show a slight change. Similar phenomenon is shown for the theoretical Cu $L_{3,2}$-edge XANES spectra (Supplementary Fig. 24c). While the substitution of N with C atoms gives rise to an emergence of two additional pre-edge peaks at ~ 930.4 eV and 950.6 eV (as highlighted by the arrows), no post-edge peaks are revealed at the energy positions of F' and G'. Those tendencies are in marked contrast to the experimental spectra. Specifically, the experimental spectrum of PS-CuN_4 only exhibits a pre-edge shoulder peak B at ~ 8987 eV at K-edge and no pre-edge peak at $L_{3,2}$-edge, which is well reproduced by the pure CuN_4C_4 structure. Besides, the experimental Cu $L_{3,2}$-edge XANES spectrum of PSB-CuN_3 shows no pre-edge peak but two post-edge resonant peaks (F' and G'), which is properly reproduced by the CuN_3C_3 structure (Supplementary Fig. 23). Thus, the probability for the presence of C atoms in the first N coordination sphere in both PS-CuN_4 and PSB-CuN_3 samples can be precluded.

In addition, the formation energies of different structures are examined (Supplementary Fig. 25, Supplementary Table 2). The CuN_4C_4 and CuN_3C_3 structures show a much lower formation energy than that of CuN_2C_2, $Cu(N_3C_1)C_4$, $Cu(N_2C_2)C_4$-1, $Cu(N_2C_2)C_4$-2 and $Cu(N_1C_3)C_4$. It confirms that the substitution of the N atoms in the first coordination sphere by C atoms is not thermodynamically preferred in present graphene-supported single-Cu-atom CuN_xC_y catalysts.

Supplementary Note 2.

According to the dipole selection rules, the *K*- (1*s* core electron) and L_3/L_2- ($2p_{3/2}/2p_{1/2}$ core electron) edges can probe directly the unoccupied electronic states of the *p* and *d* orbitals, respectively. For the planar CuN_4C_4 structure with local D_{4h} symmetry (Supplementary Fig. 21), analysis of the partial density of states in the conduction band was performed on the Cu site (Supplementary Fig. 26). It reveals that the p_{total} and d_{total} are dominated by p_z and $d_{x^2-y^2}$ orbitals, respectively. However, the *s*, p_z and $d_{x^2-y^2}$ orbitals exhibit negligible intermixing (hybridization) between each other in the conduction band. More specifically, at ~ 0.4 eV above the Fermi level (Note: this energy corresponds to the positions of peak A in *K*-edge and peak F/G in L_3/L_2-edge as shown in Supplementary Fig. 23), the p_z orbital has few electronic states, whereas the $d_{x^2-y^2}$ orbital shows a great amount of electronic states. Similarly, at ~ 1.8 eV above the Fermi level, the p_z orbital shows a noticeable amount of electronic states, but the $d_{x^2-y^2}$ orbital shows little electronic states. In addition, both p_z and $d_{x^2-y^2}$ orbitals show almost no electronic states at ~ 3.6 eV, which corresponds to the energy position for peak F'/G' in the L_3/L_2-edge (Supplementary Fig. 23b). Thus, in CuN_4C_4 structure, those characteristic profiles for the electronic states on the *p* and *d* orbitals in the conduction band result in a Cu *K*-edge XANES without obvious pre-edge peak A and a Cu L_3/L_2-edge XANES with an intense absorption peak F/G but no post-edge peak F'/G' (Supplementary Fig. 26b), which is in good agreement with the experimental spectra for PS-CuN_4 sample.

For the CuN_3C_3 structure with broken D_{4h} symmetry (i.e., C_{2v}, Supplementary Fig.

21), analysis of the partial density of states in the conduction band on the Cu site is shown in Supplementary Fig. 27. It reveals that the introduction of N vacancy along the y axis results in an obvious upshift of both p_x and p_y valence bands, with a large portion of p_y orbital moved to above the Fermi level. Therefore, in the conduction band, while d_{total} is still mainly composed of $d_{x^2-y^2}$ orbital, p_{total} is dominated by p_y orbital instead of p_z as in the case of CuN_4C_4. Furthermore, the s, p_y and $d_{x^2-y^2}$ orbitals exhibit a strong intermixing (hybridization) between each other in the conduction band. Specifically, the s, p_y and $d_{x^2-y^2}$ orbitals simultaneously demonstrate a great portion of electronic states at ~ 0.4 eV (Note: this energy corresponds to the positions for peak A in K-edge and for peak F/G in L_3/L_2-edge as shown Supplementary Fig. 23) as well as appreciable electronic states at ~ 3.6 eV (Note: this energy corresponds to the position for the peak F'/G' in L_3/L_2-edge as shown Supplementary Fig. 23b). Thus, in the defective CuN_3C_3 structure with local C_{2v} symmetry, those peculiar resonant features for the electronic states on the s, p_y and $d_{x^2-y^2}$ orbitals in the conduction band lead to a Cu K-edge XANES spectrum with prominent pre-edge peak A and a Cu L_3/L_2-edge XANES spectrum with an intense absorption peak F/G and a remarkable post-edge peak F'/G' (Supplementary Fig. 27b), which is in excellent agreement with the experimental spectra for PSB-CuN_3 sample.

For the CuN_2C_2 structure with broken D_{4h} symmetry (i.e., C_{2v}, Supplementary Fig. 21), analysis of the partial density of states in the conduction band on the Cu site is shown in Supplementary Fig. 28. It shows that the simultaneous introduction of N vacancies along the x and y axes results in an obvious upshift of both p_x and p_y valence

bands, with a large portion of p_x and p_y orbitals move to around the Fermi level. Therefore, in the conduction band, while d_{total} is still mainly composed of $d_{x^2-y^2}$ orbital, p_{total} is dominated by both p_x and p_y orbitals instead of p_z as in the case of CuN_4C_4. Furthermore, the s, p_x and p_y and $d_{x^2-y^2}$ orbitals exhibit a strong intermixing (hybridization) between each other in the conduction band. More specifically, the p_x and p_y orbitals simultaneously demonstrate a great portion of electronic states at ~ -0.1 V (Note: this energy corresponds to the positions of peak A in K-edge and for peak F/G in L_3/L_2-edge as shown Supplementary Fig. 23) as well as appreciable electronic states at ~ 2.1 eV (Note: this energy corresponds to the position of peak A' in K-edge as shown Supplementary Fig. 23b). By contrast, the $d_{x^2-y^2}$ orbital only shows a great amount of electronic states at ~ -0.1 V and the s orbital only shows an appreciable electronic states at ~ 2.1 eV, which is different to the results for CuN_4C_4 and CuN_3C_3. Thus, in the more defective CuN_2C_2 structure with local C_{2v} symmetry, those partial resonant features for the electronic states on the s, p_x, p_y and $d_{x^2-y^2}$ orbitals in the conduction band lead to a Cu K-edge XANES spectrum with prominent pre-edge peaks A and A' and a Cu L_3/L_2-edge XANES spectrum with an intense absorption peak F/G but no post-edge peak F'/G' (Supplementary Fig. 28b).

Supplementary Note 3.

To understand the contributions of different orbitals around the metal Cu centre to the binding affinity of CO_2 and their relation to the local geometric symmetry breaking, we investigated the projected density of states (PDOS) of the 4*s*, 4*p* and 3*d* orbitals for the single Cu centre and the 2*s* and 2*p* orbitals for the bound O atom in the adsorbed state of *OCHO intermediate supported on different CuN_xC_y model structures. For the symmetrical CuN_4C_4 structure (Supplementary Fig. 56), the *OCHO intermediate is adsorbed on the top site of the Cu centre, i.e., the Cu-O bond is perpendicular to the CuN_4C_4 plane. In this case, while the in-plane orbitals of Cu $3d_{xy}$ and $3d_{x^2-y^2}$ participate in forming the bonding and antibonding with the N 2*p* orbitals, the Cu $3d_{z^2}$ orbital along with the Cu $4p_z$ orbital has the maximum overlap with the O $2p_z$ orbital of *OCHO intermediate. However, when we examined the local symmetry-broken CuN_3C_3 structure due to N vacancy (Supplementary Fig. 57), it is noteworthy that the *OCHO intermediate is slantly adsorbed on the Cu centre instead of on the conventional top-site as in the case of CuN_4C_4, i.e., the Cu-O bond is not perpendicular to the CuN_3C_3 plane any more. Consequently, besides the contribution from the Cu $3d_{z^2}$ and $4p_z$ orbitals, the Cu $3d_{x^2-y^2}$, $3d_{xz}$, $3d_{xy}$, 4*s* and $4p_y$ orbitals show a drastically enhanced overlap with the O $2p_x$ and $2p_z$ orbitals. This result indicates that the stronger binding affinity of *OCHO intermediate is closely correlated to the local geometric symmetry breaking in CuN_3C_3. Furthermore, with the introduction of additional N vacancy in the asymmetrical CuN_2C_2 structure (Supplementary Fig. 58), the *OCHO intermediate is more slantly adsorbed on the Cu centre, which is associated with greater coupling

between 3*d*-, 4*s*- and 4*p*-orbitals of Cu atom and 2*s*- and 2*p*-orbitals of bonded O atom of *OCHO intermediate. Therefore, the chemical binding between the Cu centre and the intermediates is greatly influenced by the local geometric symmetry breaking of the surrounding N atoms for the metal centre.

Supplementary Note 4.

The impact on the catalytic stability from N coordination number variation is investigated. We first compared the stability of PSB-CuN_3 and PS-CuN_4 catalysts in H-cell (Supplementary Fig. 36) and flow cell (Fig. 3g and Supplementary Fig. 39). The PSB-CuN_3 shows better stability than the PS-CuN_4 in both H-cell and flow cell under the same applied potential. Particularly, after 100 h testing in flow cell at a potential of -0.95 V vs. RHE, while the PSB-CuN_3 electrode at an initial reduction current of 101 mA cm^{-2} still maintains about 94 %, the PS-CuN_4 electrode just has ~ 60 % of an initial reduction current of 25 mA cm^{-2}. It suggests that the structure of CuN_3C_3 is more electrochemically stable than that of CuN_4C_4.

In addition, it is well established that the stability issue is generally concerned about the electronic and/or the geometric structure change of the catalysts during electrocatalysis. Thus, we further monitored the dynamic structural evolution of the Cu-N site in both PSB-CuN_3 and PS-CuN_4 during the entire CO_2RR process by using operando and postmortem X-ray absorption fine structure (XAFS) spectroscopy. X-ray absorption near-edge structure (XANES) and extended-XAFS (EXAFS) spectra under open-circuit potential (OCP, stage I), catalytic state (-0.5 V and -0.8 V, state II) and the post-reacted state (OCP-return, stage III) are collected and shown in Supplementary Figs. 59 and 60.

In stage I, the Cu K-edge XANES peak of PSB-CuN_3 obtained under OCP shifts to higher energy while the intensity of the shoulder peak D is increased compared with that of pristine PSB-CuN_3 (Supplementary Fig. 59a). On the other hand, the EXAFS Fourier transform (EXAFS-FT) under OCP show a slight shift of the first major peak from 1.60 Å to 1.58 Å whereas the second satellite peak at 2.20 Å shifts to 2.26 Å (Supplementary Fig. 59b). As reported, the spectral difference of PSB-CuN_3 between pristine and OCP states could be attributed to the dynamic adsorption of CO_2 molecular on the Cu sites to form $CO_2^{\delta+}$ species[21].

In stage II, a redshift of Cu K-edge (~0.3 eV) is shown under an applied potential of -0.5 V (Inset in Supplementary Fig. 59a). And, the whole profile of the spectrum has no obvious change, suggesting the structure of Cu-N_3 well maintained during the catalytic state. Besides, the first two peaks in EXAFS-FTs exhibit a continuous shift to shorter length and an increase in intensity (Supplementary Fig. 59b), corresponding to a contraction of the Cu–N/O/C bonds with decreased bond disorder. The XANES and EXAFS spectra of PSB-CuN_3 under a more negative applied potential (-0.8 V) were obtained. It is obvious that both the XANES and EXAFS profiles of PSB-CuN_3 still remain well even under the applied potential of -0.8 V. Similar spectral evolution behaviours have also been observed in single-Ni-atom catalyst for CO_2 reduction to CO[21]. To provide the full landscape of the structural evolution, the XAFS spectrum of PSB-CuN_3 after the CO_2 electrocatalysis is collected. When the applied potential returns to OCP (stage III), both XANES and EXAFS-FTs reverse to the initial OCP state (Supplementary Fig. 59a,b). By the above discussion and analysis, it can be concluded that the applied potential and the adsorption of the intermediates (e.g., $*OCO^-$, *OCHO) could cause a reversible structural evolution/reconstruction with chemical valence and bond length change.

However, it is noteworthy that the second peak at ~ 2.20 Å in EXAFS-FTs shows a considerable overlap with the Cu–Cu peak at 2.25 Å in Cu bulk (Supplementary Fig. 59b), emphasizing the necessity to examine its components. Thus, the EXAFS wavelet-transform (EXAFS-WT) analysis is performed to clearly discriminate the coordination atoms due to its combined resolution capability in both k- and R-spaces. It is well established that the location of the intensity maximum on the k-axis is approximately proportional to the atomic number Z of the coordination atom. As shown in Supplementary Fig. 59c, only one intensity maximum at ~ 4.2 $Å^{-1}$ that matches well with the Cu-O bonding in $Cu(NO_3)_2$ reference is detected during the entire CO_2RR process, which is drastically distinct from the intensity maximum at ~ 7.0 $Å^{-1}$ for the Cu–Cu bonding in bulk Cu. It suggests that the Cu sites exist as mononuclear centers without the presence of metal-derived crystalline structures, consistent to the AC-

HAADF-STEM and XAFS results of the post-reacted PSB-CuN_3. As shown in Supplementary Figs. 61 and 62a,c, no atom aggregates can be observed on the surface of post-reacted PSB-Cu-N_3. Subsequently, quantitative EXAFS curve-fitting analysis is carried out to confirm the above-mentioned coordination assignment (Supplementary Figs. 63 and 64a, and Supplementary Table 1). The high-quality of the curve-fitting obtained by the usage of only Cu–N/O/C paths for the first two coordination shells not only rules out the formation of Cu-Cu nanocluster but also reveals a decrease in Cu-N/O and Cu-C/O interatomic distances and an increase in coordination numbers at the catalytic state (stage II), consolidating a tighter binding of the reaction intermediates (e.g., $*OCO^-$, *OCHO) under the operating conditions.

Taken together, we suggest that the PSB-Cu-N_3 electrocatalyst undergoes a reversibly dynamic structure evolution with chemical valence and bond length change during CO_2 reduction reaction. However, there is no atom aggregation or structure destroy on the Cu-N_3 moieties during the reconstruction process.

As a comparison, the operando XAFS spectra of PS-CuN_4 were collected and investigated in a parallel way (Supplementary Fig. 60). The XANES spectrum that obtained under OCP (stage I) shows slight change compared with that of the pristine PS-CuN_4, suggesting the high-valent Cu^{2+} sites are retained. Meanwhile, the EXAFS-FTs reveal a concurrent shift of the first two peaks to larger length as well. The changes under OCP are similar to that of PSB-CuN_3, which is attributed to the dynamic adsorption of CO_2 molecular on the Cu sites. With an applied potential lower than -0.5 V (stage II), there is a redshift of ~0.6 eV in the Cu K-edge (Inset in Supplementary Fig. 60a), indicating an obvious electrochemical reduction. And, as shown in Supplementary Fig. 60b, the first two peaks in EXAFS-FTs of PS-CuN_4 show a continuous shift to shorter length with increased intensity. When a potential of -0.8 V was applied on PS-CuN_4, the first major peak and the second satellite peak in EXAFS-FTs show a significant shift from 1.64 Å to 1.50 Å and from 2.25 Å to 2.29 Å, respectively. The EXAFS-WT reveals a large shift of the intensity maximum from ~ 4.2 $Å^{-1}$ to ~ 5.2 $Å^{-1}$ during the catalytic stage, suggesting the formation of fractional

Cu-Cu bonding. When the applied potential returns to OCP (stage III), the EXAFS-FTs cannot go back to the initial state and the Cu-Cu signal in EXAFS-WT is still retained, indicating that the dynamic formation of fractional Cu-Cu nanocluster is irreversible. Finally, the AC-HAADF-STEM and XAFS measurement were employed to investigate the post-reacted PS-CuN_4. The partial aggregates can be observed in the AC-HAADF-STEM image (Supplementary Fig. 65) and EXAFS spectra (Supplementary Fig. 62b,c) of the post-reacted PS-CuN_4. Further quantitative EXAFS curve-fitting analysis by the usage of both Cu–N/O and Cu–Cu paths also confirm the presence of fractional Cu-Cu bonding (Supplementary Figs. 64b and 66, Supplementary Table 1). Therefore, it can be concluded that PS-CuN_4 with high-valent Cu^{2+} could cause a dramatically irreversible structure evolution, leading to the aggregation of single atoms.

All in all, by combining operando and postmortem XAFS with AC-HAADF-STEM characterization, it can be confirmed that the surface reconstruction/evolution occurs on both PSB-CuN_3 and PS-CuN_4 during CO_2 electrocatalysis. However, the changes in the electronic and geometric structure caused by the surface reconstruction are highly related to the physiochemical properties of the samples and the applied potentials. Compared with the PS-CuN_4 with high valence Cu^{2+}, the structure change of low-valent Cu^{1+} PSB-CuN_3 is slight. And, the surface evolution process of PSB-CuN_3 is reversible and no atoms aggregation can be observed after CO_2 electrocatalysis. On contrary, the dramatic surface evolution for PS-CuN_4 would lead to the aggregation of fractional Cu atoms and it is irreversible. Therefore, the electrochemical stability of PSB-CuN_3 is better than that of PS-CuN_4. Those results are consistent with the published results that the low-valent Ni^{1+} single-atom catalysts would not reconstruct into nanoparticles for CO_2RR to CO[21], but the high-valent Cu^{2+} single-atom catalysts would reconstruct to nanoparticles in the electrochemical reduction of nitrate to ammonia and electrocatalytic oxygen reduction[22,23].

Supplementary References

1. Zhang, J., Yin, R., Shao, Q., Zhu, T. & Huang, X. Oxygen vacancies in amorphous InO_x nanoribbons enhance CO_2 adsorption and activation for CO_2 electroreduction. *Angew Chem. Int. Ed.* **58**, 5609-5613 (2019).

2. Koh, J. H. *et al.* Facile CO_2 electro-reduction to formate via oxygen bidentate intermediate stabilized by high-index planes of Bi dendrite catalyst. *ACS Catal.* **7**, 5071-5077 (2017).

3. Su, P. *et al.* Ultrathin bismuth nanosheets as a highly efficient CO_2 reduction electrocatalyst. *ChemSusChem* **11**, 848-853 (2018).

4. Jiang, Z. *et al.* Discovery of main group single Sb–N_4 active sites for CO_2 electroreduction to formate with high efficiency. *Energy Environ. Sci.* **13**, 2856-2863 (2020).

5. Feng, X. *et al.* Bi_2O_3/BiO_2 nanoheterojunction for highly efficient electrocatalytic CO_2 reduction to formate. *Nano Lett.* **22**, 1656–1664 (2022).

6. Zheng, T. *et al.* Copper-catalysed exclusive CO_2 to pure formic acid conversion via single-atom alloying. *Nat. Nanotechnol.* **16**, 1386-1393 (2021).

7. Zhang, A. *et al.* Harmonizing the electronic structures of the adsorbate and catalysts for efficient CO_2 reduction. *Nano Lett.* **19**, 6547-6553 (2019).

8. Lei, F. *et al.* Metallic tin quantum sheets confined in graphene toward high-efficiency carbon dioxide electroreduction. *Nat. Commun.* **7**, 12697 (2016).

9. Deng, P. *et al.* Metal–organic framework-derived carbon nanorods encapsulating bismuth oxides for rapid and selective CO_2 electroreduction to formate. *Angew*

Chem. Int. Ed. **59**, 10807-10813 (2020).

10. Fan, L., Xia, C., Zhu, P., Lu, Y. & Wang, H. Electrochemical CO_2 reduction to high-concentration pure formic acid solutions in an all-solid-state reactor. *Nat. Commun.* **11**, 3633 (2020).

11. Zhang, X. *et al.* Defects and conductive nitrogen-carbon framework regulated $ZnInO_x$ nanosheets for boosting CO_2 electrocatalytic reduction. *Appl. Catal. B* **279**, 119383 (2020).

12. Liu, S. *et al.* Efficient electrochemical reduction of CO_2 to HCOOH over sub-2 nm SnO_2 quantum wires with exposed grain boundaries. *Angew Chem. Int. Ed.* **58**, 8499-8503 (2019).

13. Miao, C.-C. & Yuan, G.-Q. Morphology-controlled Bi_2O_3 nanoparticles as catalysts for selective electrochemical reduction of CO_2 to formate. *ChemElectroChem* **5**, 3741-3747 (2018).

14. Liu, S., Lu, X. F., Xiao, J., Wang, X. & Lou, X. W. Bi_2O_3 nanosheets grown on multi-channel carbon matrix to catalyze efficient CO_2 electroreduction to HCOOH. *Angew Chem. Int. Ed.* **58**, 13828-13833 (2019).

15. Ma, W. *et al.* Promoting electrocatalytic CO_2 reduction to formate via sulfur-boosting water activation on indium surfaces. *Nat. Commun.* **10**, 892 (2019).

16. Gong Q*, et al.* Structural defects on converted bismuth oxide nanotubes enable highly active electrocatalysis of carbon dioxide reduction. *Nat. Commun.* **10**, 2807 (2019).

17. Grigioni I*, et al.* CO_2 electroreduction to formate at a partial current density of 930

mA cm^{-2} with InP colloidal quantum dot derived catalysts. *ACS Energy Lett.* **6**, 79-84 (2021).

18. Creissen CE, Rivera de la Cruz JG, Karapinar D, Taverna D, Schreiber MW, Fontecave M. Molecular inhibition for selective CO_2 conversion. *Angew. Chem. Int. Ed.* **61**, e202206279 (2022).

19. Chen Y*, et al.* A robust, scalable platform for the electrochemical conversion of CO_2 to formate: identifying pathways to higher energy efficiencies. *ACS Energy Lett.* **5**, 1825-1833 (2020).

20. Sen S, Brown SM, Leonard M, Brushett FR. Electroreduction of carbon dioxide to formate at high current densities using tin and tin oxide gas diffusion electrodes. *J. Appl. Electrochem.* **49**, 917-928 (2019).

21. Yang HB*, et al.* Atomically dispersed Ni(i) as the active site for electrochemical CO_2 reduction. *Nat. Energy* **3**, 140-147 (2018).

22. Yang J*, et al.* Potential-driven restructuring of Cu single atoms to nanoparticles for boosting the electrochemical reduction of nitrate to ammonia. *J. Am. Chem. Soc.* **144**, 12062-12071 (2022).

23. Xing G*, et al.* Reconstruction of highly dense Cu−N_4 active sites in electrocatalytic oxygen reduction characterized by operando synchrotron radiation. *Angew. Chem. Int. Ed.* **61**, e202211098 (2022).
